# The impact of cysteine on lifespan in three model organisms: A systematic review and meta‐analysis

**DOI:** 10.1111/acel.14392

**Published:** 2024-10-30

**Authors:** Yue Ma, Mengqi Chen, Kaiyao Huang, Wakam Chang

**Affiliations:** ^1^ Faculty of Health Sciences University of Macau Taipa Macau China; ^2^ MOE Frontier Science Centre for Precision Oncology University of Macau Taipa Macau China; ^3^ Key Laboratory of Algal Biology Institute of Hydrobiology, Chinese Academy of Sciences Wuhan Hubei China

**Keywords:** aging, anti‐aging, cysteine, lifespan, NAC, survival

## Abstract

Cysteine is an amino acid present in thiol proteins and often dictates their secondary structures. Although considered nonessential, cysteine may be essential for patients with certain metabolic diseases and can reduce the requirement for dietary methionine. Cysteine and some of its derivatives, such as N‐acetylcysteine, are considered antioxidants and widely used in animal aging studies. To provide insights into the potential anti‐aging effects of cysteine, we systematically reviewed and performed a meta‐analysis to investigate the impact of cysteine supplementation on lifespan using three model organisms: mice, nematodes, and fruit flies. A total of 13 mouse studies, 13 *C. elegans* studies, and 5 *Drosophila* studies were included in the analysis. The findings revealed that cysteine supplementation significantly reduced the risk of mortality in mice and *C. elegans*. Subgroup analysis showed consistent results across different starting times and administration methods and revealed adverse effects of high doses on worms and a lack of effect in nondisease mouse models. Similar to mice, the effects of cysteine supplementation on *Drosophila* were not statistically significant, except in transgenic flies. The study identified certain limitations, including the quality of the included studies and the potential for publication bias. We also discussed uncertainties in the underlying molecular mechanisms and the clinical application of dietary cysteine.

## Graphical abstract

We conducted a meta‐analysis to investigate the impact of cysteine supplementation on lifespan in mice, nematodes, and fruit flies. Positive effects were observed in *C*. *elegans*, transgenic flies, and disease model mice, but not in wild‐type flies and wild‐type mice. Subgroup analysis revealed adverse effects of high doses on nematodes and fruit flies.

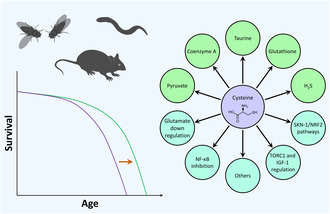

**Abbreviations**
AFT: accelerated failure time
ASSNAC: S‐allylmercapto‐N‐acetylcysteine
FDR: false discovery rate
FUdR: 5'‐fluorodeoxyuridine
HR: Hazard Ratio
IQR: interquartile range
MR: meta‐regression
NAC: N‐acetylcysteine
NF‐κB: nuclear factor‐κB
NRF2: nuclear factor erythroid 2‐related factor 2
ROS: reactive oxygen species
SAC: S‐allylcysteine
SAMC: S‐allylmercaptocysteine

## 1 INTRODUCTION

Cysteine, a nonessential amino acid, plays a pivotal role in diverse biological processes. It is categorized as nonessential because it can be synthesized by the body from other amino acids, such as methionine. Dietary cysteine greatly reduces the methionine requirement (Dong et al., 2018). Cysteine contributes to a wide range of cellular processes. In addition to being a fundamental building block for proteins, it participates in the synthesis of glutathione, a critical antioxidant that safeguards cells against oxidative damage (Bonifácio et al., 2021). The thiol group of cysteine is highly reactive, so cysteine itself can reduce harmful substances like reactive oxygen species (ROS) and heavy metal ions from the body (Tenório et al., 2021). Additionally, cysteine acts as a substrate for the production of essential molecules like coenzyme A and hydrogen sulfide (H_2_S), thus contributing to various physiological functions, such as metabolism, ATP production, and epigenetic regulation (Bonifácio et al., 2021; Jouandin et al., 2022). Another metabolite synthesized from cysteine, taurine, has been demonstrated to profoundly impact multiple physiological functions and slow down the aging process (Singh et al., 2023).

**FIGURE 1:**
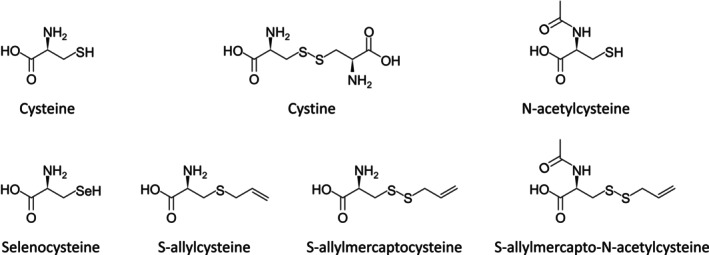
Chemical structures of cysteine and cysteine‐derived molecules.

Several supplemental forms of cysteine have been used to boost cysteine levels (Figure 1). N‐acetylcysteine (NAC) is a synthetic derivative of cysteine that has gained approval from the U.S. Food and Drug Administration and recognition as an essential medicine by the World Health Organization. NAC is widely available as an over‐the‐counter nutritional supplement in countries such as the United States, Canada, and Australia, and its affordability and commercial appeal make it a popular nutraceutical (Ooi et al., 2018). NAC is regarded as a promising anti‐aging drug due to its demonstrated potential in improving age‐related hearing loss, memory decline, spatial memory deficits, and oocyte aging in mouse models (Costa et al., 2016; Kulkarni et al., 2022; Liu et al., 2012; Marie et al., 2018; More et al., 2018). Moreover, NAC has been found to effectively reduce hallmarks of aging, including oxidative stress levels and neurodegeneration, in the rat brain (Garg et al., 2018). In recent years, multiple clinical trials have demonstrated the efficacy of NAC in improving various aging‐related diseases, such as Alzheimer's disease, type 2 diabetes, cardiovascular disease, and breast cancer (Deepmala et al., 2015; Kwon, 2021; Lasram et al., 2015; Sochman, 2002). Additionally, clinical trials have revealed that the supplementation of glycine and NAC in elderly individuals can improve aging‐related biochemical markers and phenotypes, including glutathione deficiency, oxidative stress, mitochondrial dysfunction, inflammation, insulin resistance, endothelial dysfunction, body fat, muscle strength, gait speed, and cognitive function (Kumar et al., 2023; Sekhar et al., 2011). However, it should be noted that direct evidence supporting the extension of human lifespan by NAC remains lacking.

Besides NAC, several other cysteine derivatives and natural extracts are antioxidants and their effects on aging have been demonstrated. Selenocysteine is a proteinogenic amino acid and is present in 25 human selenoproteins (Zhang et al., 2020). Most of these proteins function as oxidoreductases and the selenocysteine residues are critical for their catalytic activities. Selenocysteine was shown to delay aging in *C. elegans* (Kim et al., 2017, 2018). Both S‐allylcysteine (SAC) and S‐allylmercaptocysteine (SAMC) are garlic‐derived amino acids and antioxidants; they are beneficial to diseases like neurodegenerative disorders (Colín‐González et al., 2012). The influence of SAC on the lifespan of *C. elegans* was inconsistent but it appeared to reduce ROS levels and improve the locomotive activity of the aged worms (Kim & Park, 2016; Ogawa et al., 2016). SAMC was shown to increase the lifespan of worms (Ogawa et al., 2016). S‐allylmercapto‐N‐acetylcysteine (ASSNAC), an NAC derivative containing an S‐allylmercaptan group that increases its cell and tissue permeability, can activate the nuclear factor erythroid 2‐related factor 2 (NRF2) anti‐oxidative pathways (Savion et al., 2014). Its lifespan‐extending effects were shown in worms (Savion et al., 2018).

The anti‐aging capacity of these compounds is not without question. First, the outcomes of the related studies are not always consistent. In fact, conflicting results were reported even with the same species and experimental designs. Second, the gender‐ and dose‐dependent effects of these compounds were not always considered. This is concerning because NAC, for example, does exhibit detrimental impacts at higher doses (Liou et al., 2021; Shaposhnikov et al., 2018). Third, the benefits of these compounds may depend on specific contexts. For example, NAC treatment increases exercise performance but only in people with low glutathione (Paschalis et al., 2018). It may hinder early adaptive responses in human skeletal muscle (Petersen et al., 2012). Fourth, the use of specific species/strains increases the possibility of overgeneralizing the strain‐specific conclusions (Flurkey et al., 2010). Many of the published studies, especially those in mice, utilized disease models, and their interpretation in normal aging is unclear. Fifth, the modes of action of these compounds are not well defined. Although many studies emphasized their antioxidative properties and suggested that they function by regulating the redox conditions, the antioxidative activities of these compounds are not all high under physiological conditions and may function indirectly, such as through the synthesis of glutathione or the activation of the NRF2 pathway (Tenório et al., 2021). Lastly, although ROS are generally considered harmful, they are essential signaling molecules, and excessive antioxidants can in fact accelerate aging (Gusarov et al., 2021; Wei & Kenyon, 2016; Yang & Hekimi, 2010).

Due to the difficulties in studying these compounds in humans, it is more practical to study them using model organisms, such as mice, nematodes, and fruit flies. However, even though the experiments are generally performed in controlled laboratory settings, the outcomes can vary considerably. The general interest in the lifespan‐extending effects of cysteine‐related compounds and the lack of a systematic review and quantitative meta‐analysis of the existing literature prompt us to evaluate the current evidence regarding cysteine and its derivatives on lifespan in mice, *C. elegans*, and *Drosophila melanogaster*, the three most studied preclinical models of aging. The objective of the present study is to address this gap by estimating the overall effect of cysteine on natural lifespan, examining potential heterogeneity in this effect among these organisms, and identifying sources of heterogeneity.

## 2 METHODS

### 2.1 Overall design of the systematic review

This review followed the PRISMA 2020 checklist for systematic reviews and meta‐analyses (Table S1, Page et al., 2021) and recommendations for systematic reviews of preclinical data studies (Sena et al., 2014). Study quality was assessed as previously described (Macleod et al., 2004; Vesterinen et al., 2014). This study was registered with the PROSPERO database of prospective systematic reviews (Registration number: CRD42024507152). All data can be obtained from the corresponding author upon request.

### 2.2 Literature search

We conducted a systematic search of PubMed, Web of Science, and Google Scholar to identify articles investigating the impact of cysteine derivatives on lifespan in mice, *C. elegans*, and *Drosophila*. The search strategies used are provided in Table S2 and the final search was conducted on July 1, 2024. We also searched the references in the included articles and included any additional relevant studies. Two authors (Y.M. and M.C.) independently screened the abstracts, and articles without survival data (such as hazard ratios (*HRs*) and raw survival curves) were excluded. Duplicate studies were excluded. We also excluded studies that administered cysteine derivatives to mice via injection instead of oral intake.

Study characteristics, including the year of publication, species and strain, the type of cysteine derivatives, the starting age of treatment, the method of delivery, and any special experimental characteristics were extracted. For mouse studies, we included the gender, whether the strain was inbred, hybrid, or outbred, whether the mouse was transgenic, and whether it was a disease model. For nematode studies, we included the strain of *E. coli* in diets and whether it was dead or alive, and whether 5′‐fluorodeoxyuridine (FUdR), which affects lifespan in *C. elegans* (Wang et al., 2019), was used. For studies in fruit files, we recorded the gender of the files.

### 2.3 Data extraction

For each study, we contacted the authors for raw survival data. If such data was not available, we extracted reported Cox Proportional *HRs* and raw survival data. Reported Kaplan–Meier survival curves were converted into x‐y data using Engauge Digitizer (https://github.com/markummitchell/engauge‐digitizer/releases) and the results were transformed into survival data using IPDfromKM, which reconstructs individual survival data using an iterative algorithm adapted from the K‐M estimation method (Guyot et al., 2012). The root mean square error, which measures the difference in survival probabilities calculated using reconstructed data and original data, along with the Kolmogorov–Smirnov test statistics and p‐values, was used to compare the distributions of the read‐in and estimated survival curves to assess the accuracy of the individual survival data reconstruction (Liu et al., 2021).

We performed the survival calculations using the survival package in R by Therneau et al. (https://cran.r‐project.org/web/packages/survival/index.html, version 3.5–8). The outcomes were the *HRs* under the Cox proportional model (Bender et al., 2005) and the deceleration factors (percent changes in survival) under the accelerated failure time models (Parish & Swindell, 2022; Swindell, 2009). Proportional hazards tests and log–log plots were examined to ensure that the Cox proportional model was adequate (Grambsch & Therneau, 1994).

### 2.4 Meta‐analysis

All calculations were conducted using the R programming language. (version 4.2.3). To account for variation across experiments, we employed a random‐effects meta‐analysis using the Hartung‐Knapp‐Sidik‐Jonkman estimator because of its low Type I error rate (IntHout et al., 2014; Parish & Swindell, 2022). Using the meta package in R (Balduzzi et al., 2019), extracted *HRs* and deceleration factors were log‐transformed to improve the homogeneity of data variance and to make the data closer to a normal distribution. Their heterogeneity was assessed using Higgins and Thompson's *I* ^2^ statistic and Cochran's *Q* test (Higgins & Thompson, 2002; von Hippel, 2015).

To evaluate the possibility of publication bias or the influence of small studies, contour‐enhanced funnel plots and Egger's test of funnel plot asymmetry were employed (Sterne et al., 2011). If there is evident publication bias, the trim and fill method was employed to identify and rectify the asymmetry in the funnel plot caused by such bias. For studies that had multiple experiments, we chose the single experiment with the largest sample size as a representative of the study. Funnel plot analysis was conducted using the metafor package in R (Viechtbauer, 2010).

The nominal Type I error rate for all statistical tests was set at *α* = 0.05. The Benjamini‐Hochberg procedure was used to control the false discovery rate (FDR) for multiple hypothesis testing across meta‐analysis subgroups (Benjamini & Hochberg, 1995). Galbraith radial and Baujat plots were used to identify the sources of heterogeneity (Baujat et al., 2002; Galbraith, 1988). Sensitivity analyses were conducted by sequentially excluding one study at a time to assess overall homogeneity.

## 3 RESULTS

### 3.1 The literature search identified 31 lifespan studies

Our search (see Table S1 for the PRISMA 2020 checklist and Table S2 for the search strategies) has identified a total of 1471 studies (see supplemental file) and, after screening the titles and abstracts, 495 (33.7%) cancer studies, 383 (26.0%) human studies, 184 (12.5%) cell studies, 164 (11.1%) plant studies, and 17 (1.2%) studies in other organisms were excluded. Additionally, 104 (7.1%) reviews without new data and 98 (6.7%) studies without aging‐related survival data were excluded. Five studies from other sources were ultimately included. This resulted in a final selection of 13 mouse studies, 13 nematode studies, and 5 fruit fly studies (Table S3). The PRISMA flow chart illustrating this process is presented in Figure S1.

Two of the mouse studies were from the Harman group (Harman, 1957, 1961) and three using transgenic mice were from the Miao group (Chen et al., 2019, 2020; Jin et al., 2014). Two worm studies were from the Hekimi group (Desjardins et al., 2017; Yang & Hekimi, 2010) and five were from the Park group (Kim et al., 2017, 2018; Kim & Park, 2016; Oh et al., 2015; Oh & Park, 2017). All the other studies, including all *Drosophila* studies, were independent.

The included studies exhibited varying quality, as indicated by the median CAMARADES quality scores. For mouse studies, the median quality score was 4 (interquartile range [IQR]: 2–4), for nematode studies it was 4 (IQR: 4–4), and for *Drosophila* studies, it was 3 (IQR: 3–3). Notably, none of the studies reported blinding and allocation concealment. Regarding sample size calculations, three out of thirteen mouse studies (23%) described them (Table S3).

We extracted data from 25 independent lifespan experiments from the mouse studies, 98 from the *C. elegans* studies, and 214 from the *Drosophila* studies. Detailed information on the baseline characteristics of the studies and lifespan experiments can be found in Tables S4 (mouse), S5 (*C. elegans*), and S6 (*Drosophila*).

### 3.2 Overall and subgroup analyses for studies in mice

**FIGURE 2:**
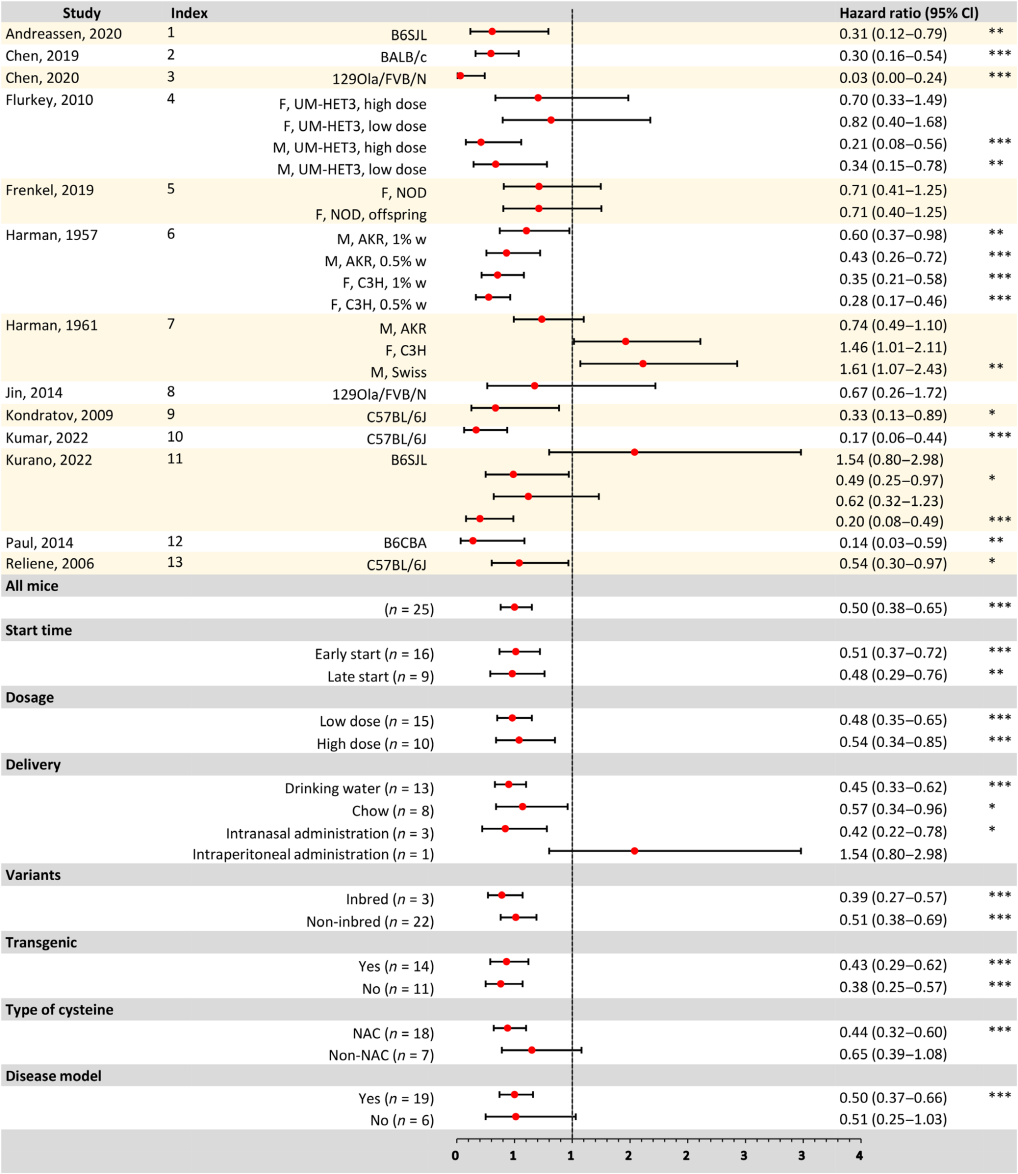
A Forest plot illustrating hazard ratios for all mouse experiments, including specific subgroups. The left side of the plot presents comprehensive details, including the primary author, publication year, study index (see Table S7a), and specific experiment characteristics such as sex, genotype, and cysteine dose administration. On the right side, *HRs* and their corresponding 95% confidence intervals (CIs) are listed. The bottom section of the plot presents a summary of the meta‐analysis effects for all experiments (*n* = 25) as well as specific subgroups. Asterisks indicate levels of statistical significance (*for *p* < 0.05, **for *p* < 0.01, ***for *p* < 0.001 after the Benjamini‐Hochberg correction for multiple comparisons).

Among the 25 experiments conducted on mice, cysteine was found to significantly reduce the risk of death in 15 (60%) experiments (*p* < 0.05). There was significant heterogeneity observed (*I* ^2^ = 78%, 68%–85%, *p* < 0.001). A random effects inverse variance weighted meta‐analytic model was used to estimate a pooled *HR* of 0.50 (0.38–0.65, *p* < 0.0001, *n* = 25) (Figure 2).

Based on the distribution of parameters in the available experiments, we defined a “high dose” of cysteine as an effective body weight‐based dose of 600 mg/kg or higher; and “early” dosing as when mice started dosing before 12 weeks (84 days). High doses were used in 10 of 25 experiments (40%) and early dosing was used in 16 (64%) experiments. Mice received cysteine daily orally (*n* = 21, 84%), through drinking water (*n* = 13, 52%) or mixed with food (*n* = 8, 32%), or through intraperitoneal and intranasal routes (*n* = 4, 16%). NAC was used in 18 (72%) experiments and cysteine hydrochloride was used in 7 (28%) experiments. And 6 experiments (24%) used nondisease mouse models, 19 experiments (76%) used mice with disease models. In addition, 14 (56%) experiments used transgenic mice, and 11 (44%) used nontransgenic mice.

Subgroup analysis revealed that regardless of early start (*HR* = 0.51, 0.37–0.72, *FDR* <0.001, *n* = 16) or late start (*HR* = 0.48, 0.29–0.76, *FDR* = 0.0021, *n* = 9), low dose (*HR* = 0.48, 0.35–0.65, *FDR* <0.001, *n* = 15) or high dose (*HR* = 0.54, 0.34–0.85, *FDR* <0.001, *n* = 10), oral administration (*HR* = 0.49, 0.36–0.65, *FDR* <0.001, *n* = 21) including administration through drinking water (*HR* = 0.45, 0.33–0.60, *FDR* <0.001, *n* = 13) or chow (*HR* = 0.57, 0.34–0.96, *FDR* = 0.036, *n* = 8) or intranasal administration (*HR* = 0.42, 0.22–0.78, *FDR* = 0.0116, *n* = 3), the use of inbred (*HR* = 0.39, 0.27–0.57, *FDR* <0.001, *n* = 3) or noninbred mice (*HR* = 0.51, 0.38–0.69, *FDR* <0.001, *n* = 22), the use of nontransgenic (*HR* = 0.38, 0.25–0.57, *FDR* <0.001, *n* = 11) or transgenic mice (*HR* = 0.43, 0.29–0.62, *FDR* <0.001, *n* = 14), the use of NAC (*HR* = 0.44, 0.32–0.60, *FDR* <0.001, *n* = 18), the use of disease model (*HR* = 0.50, 0.37–0.66, *FDR* <0.001, *n* = 19), cysteine was found to significantly reduce the risk of death in experimental mice. However, in experiments using cysteine hydrochloride (*HR* = 0.65, 0.39–1.08, *FDR* = 0.096, *n* = 7), though intraperitoneal administration (*HR* = 1.54, 0.80–2.98, *FDR* = 0.195, *n* = 1), and in nondisease model (*HR* = 0.51, 0.25–1.03, *FDR* = 0.0616, *n* = 6), no significant effect on the risk of death was observed (Figure 2).

We then conducted a meta‐regression (MR) analysis on the original doses of each study, without classifying them into high and low doses. Our findings indicate a significant positive correlation between dose and the increase in *HR* (regression coefficient = 0.0001, 0–0.0001, MR *p* = 0.0336). At the same time, no significant differences were observed based on start time (*HR* = 0.51 vs. 0.48, MR *p* = 0.7631), dose (*HR* = 0.48 vs. 0.54, MR *p* = 0.5529), genotype differences (*HR* = 0.39 vs. 0.50, MR *p* = 0.4712), disease model (*HR* = 0.39 vs. 0.51, MR *p* = 0.7202) and use of transgenic mice (*HR* = 0.38 vs. 0.43, MR *p* = 0.2286). However, the administration of NAC or cysteine hydrochloride had slightly different effects on mouse survival (*HR* = 0.44 vs. 0.65, MR *p* = 0.1562), and intraperitoneal administration had a different effect on mouse survival compared with other drug administration methods (*HR* = 1.54 vs. 0.60, MR *p* = 0.0739), although the difference was not statistically significant (Figure S2).

Consistent with the hazard ratio findings, cysteine exhibited a significant association with the accelerated failure time (AFT) deceleration factor in mice across all combined experiments (*D* = 1.21, 1.11–1.32, *p* < 0.0001, *n* = 25), indicating a substantial prolongation of mouse survival through cysteine administration. Subgroup analysis revealed that regardless of early initiation (*D* = 1.27, 1.11–1.45, *FDR* = 0.0006, *n* = 16) or delayed initiation (*D* = 1.13, 1.03–1.24, *FDR* = 0.0093, *n* = 9), low dose (*D* = 1.23, 1.07–1.41, *FDR* = 0.0034, *n* = 15) or high dosage (*D* = 1.19, 1.07–1.34, *FDR* = 0.0018, *n* = 10), administration via drinking water (*D* = 1.32, 1.14–1.53, *FDR* = 0.0016, *n* = 13), food mixture (*D* = 1.18, 1.04–1.35, *FDR* = 0.0173, *n* = 8), or intranasal administration (*D* = 1.03, 1.02–1.05, *FDR* = 0.0172, *n* = 3), the use of inbred strains (*D* = 1.30, 1.19–1.43, *FDR* <0.0001, *n* = 3) or noninbred mice (*D* = 1.20, 1.09–1.33, *FDR* = 0.0004, *n* = 22), the use of nontransgenic mice (*D* = 1.19, 1.07–1.32, *FDR* = 0.0018, *n* = 11) or transgenic mice (*D* = 1.24, 1.07–1.43, *FDR* = 0.0039, *n* = 14), the use of NAC (*D* = 1.24, 1.10–1.38, *FDR* = 0.0006, *n* = 18) or cysteine hydrochloride (*D* = 1.16, 1.00–1.35, *FDR* = 0.045, *n* = 7), the use of disease model (*D* = 1.23, 1.10–1.37, *FDR* = 0.0006, *n* = 19) or nondisease model (*D* = 1.17, 1.00–1.37, *FDR* = 0.0452, *n* = 6), cysteine consistently demonstrated a significant extension of the survival time in experimental mice. However, intraperitoneal administration (*D* = 0.97, 0.95–1.00, *FDR* = 0.05, *n* = 1) had no significant effect on mouse survival.

### 3.3 Overall and subgroup analyses for studies in *C. Elegans*

**FIGURE 3:**
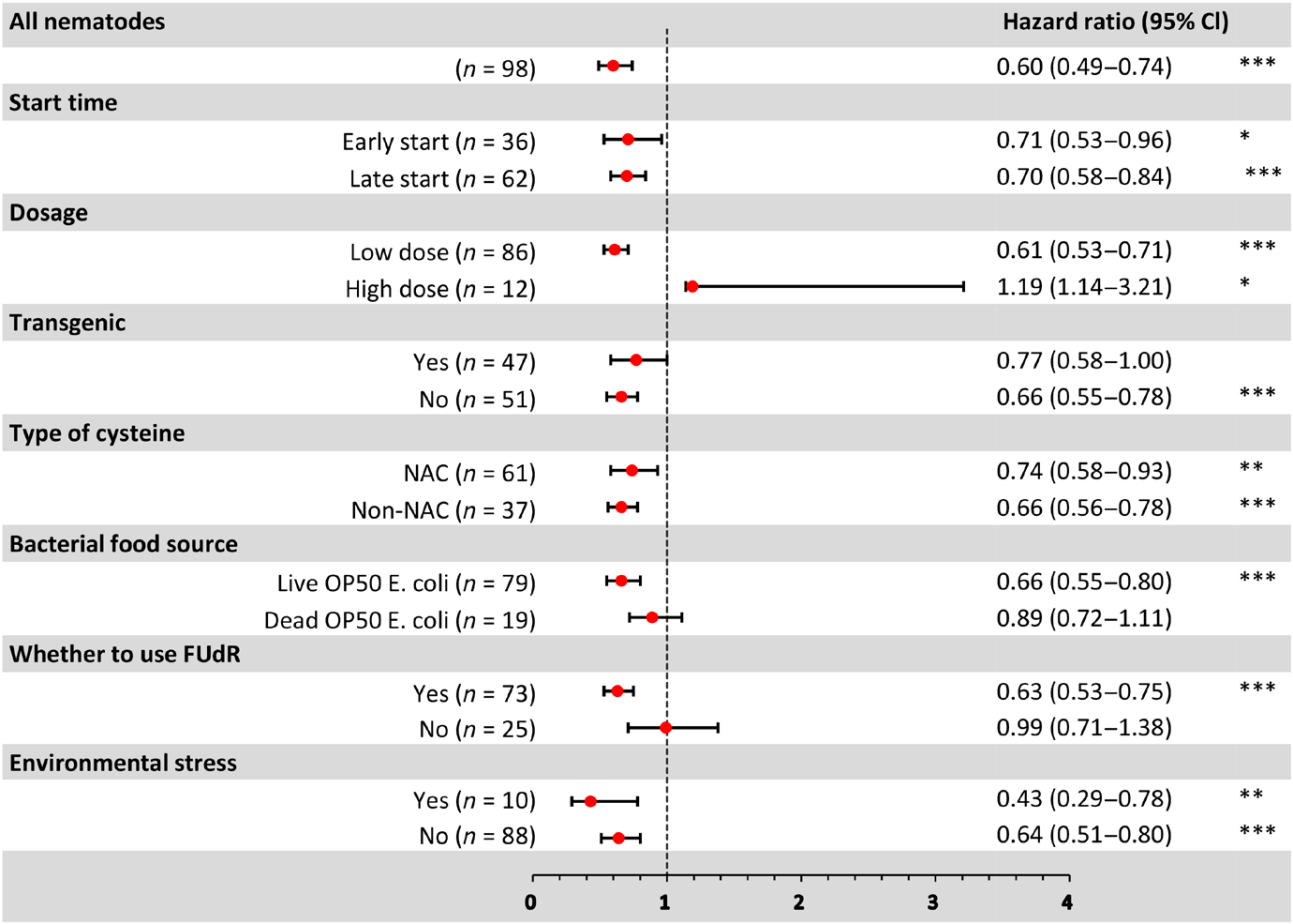
A Forest plot summarizing the meta‐analytic effects for all *C. elegans* experiments and specific subgroups. The left side provides a description including the primary author, publication year, experiment details, strain, food source, environmental stress, and cysteine dose. On the right side, *HRs* and their corresponding 95% CIs are listed, with asterisks indicating levels of statistical significance (*for *p* < 0.05, **for *p* < 0.01, ***for *p* < 0.001 after the Benjamini‐Hochberg correction for multiple comparisons).

Among 98 experiments in *C. elegans*, 56 (57.1%) reported a significant reduction in the risk of mortality with cysteine administration (*p* < 0.05). A high degree of heterogeneity was observed (*I* ^2^ = 97%, 96%–97%, *p* < 0.0001), and the pooled *HR* was estimated to be 0.71 (0.60–0.83, *p* < 0.0001, *n* = 98) (Figure 3 and S3).

All these experiments used the N2 strains. Experiments we classified as “early” started dosing at the larval stage and included 32 (32.6%) experiments. We defined a “high dose” of cysteine as concentrations equal to or greater than 10 mM, which were used in 12 (6.5%) experiments. Live OP50 *E. coli* served as the food source in 79 (80.6%) experiments, while the remaining 19 (19.4%) experiments utilized dead OP50 *E. coli*. NAC administration was employed in 61 (62.2%) experiments, while the remaining experiments employed other cysteine derivatives, including selenocysteine, SAC, SAMC, and ASSNAC. In 73 (74.5%) experiments, FUdR was employed to inhibit nematode reproduction. Environmental stressors, such as oxidative stress, were induced in 10 (10.2%) experiments using H_2_O_2_, heat, UV, paraquat, or high glucose. Transgenic nematodes were used in 47 experiments (48.0%).

Subgroup analysis revealed that regardless of the use of NAC (*HR* = 0.74, 0.58–0.93, *FDR* = 0.01, *n* = 61) or other cysteine derivatives (*HR* = 0.66, 0.56–0.78, *FDR* <0.0001, *n* = 37), induced environmental stress (*HR* = 0.47, 0.29–0.78, *FDR* = 0.0032, *n* = 10) or no induced environmental stress (*HR* = 0.74, 0.63–0.87, *FDR* = 0.0006, *n* = 88), nontransgenic nematodes (*HR* = 0.66, 0.55–0.78, *FDR* = 0.0002, *n* = 51), food source being Live OP50 *E. coli* (*HR* = 0.66, 0.55–0.80, FDR = 0.0002, *n* = 79), the use of FUdR (*HR* = 0.63, 0.53–0.75, *FDR* = 0.0002, *n* = 73), early start (*HR* = 0.71, 0.53–0.96, *FDR* = 0.0252, *n* = 36), late start (*HR* = 0.70, 0.58–0.84, *FDR* = 0.002, *n* = 62), or low doses (*HR* = 0.61, 0.53–0.71, *FDR* = 0.0002, *n* = 86), cysteine consistently demonstrated a significant reduction in the risk of mortality in experimental *C. elegans*. However, transgenic nematodes (*HR* = 0.77, 0.58–1.00, *FDR* = 0.052, *n* = 47), food source being dead OP50 *E. coli* (*HR* = 0.89, 0.72–1.11, *FDR* = 0.3193, *n* = 19), and no use of FudR (*HR* = 0.99, 0.71–1.38, *FDR* = 0.9426, *n* = 25) did not have a significant effect on the risk of *C. elegans* mortality. Additionally, high doses (*HR* = 1.19, 1.14–3.21, *FDR* = 0.0135, *n* = 12) increased the risk of *C. elegans* mortality (Figure 3).

In the MR analysis, no significant differences were found in the survival effect of *C. elegans* based on the difference in start time (*HR* = 0.71 vs. 0.70, MR *p* = 0.9021), the type of cysteine (*HR* = 0.74 vs. 0.66, MR *p* = 0.4661), whether transgenic nematodes were used (*HR* = 0.77 vs. 0.66, MR *p* = 0.324), whether environmental stress was induced (*HR* = 0.47 vs. 0.74, MR *p* = 0.0981) and food sources (*HR* = 0.66 vs. 0.89, MR *p* = 0.1386). However, there were significant differences in *C. elegans* survival based on whether FudR was used (*HR* = 0.63 vs. 0.99, MR *p* = 0.0119). Doses (regression coefficient = 1.70, 1.06–2.34, MR *p* < 0.0001) were significantly positively correlated with increases in *HR* (Figure S4).

Consistent with the hazard ratio findings, the analysis of the AFT deceleration factor in *C. elegans* revealed a significant association with cysteine across all experiments combined (*D* = 1.08, 1.04–1.12, *p* < 0.0001, *n* = 98). This indicates that administering cysteine significantly prolonged the survival time of *C. elegans*. Subgroup analysis indicated that regardless of the use of NAC (*D* = 1.06, 1.05–1.07, *FDR* <0.0001, *n* = 61) or other cysteine derivatives (*D* = 1.07, 1.02–1.13, *FDR* = 0.0119, *n* = 37), induced environmental stress (*D* = 1.18, 1.07–1.31, *FDR* = 0.0008, *n* = 10) or no induced environmental stress (*D* = 1.07, 1.03–1.11, *FDR* = 0.0006, *n* = 88), nontransgenic nematodes (*D* = 1.11, 1.07–1.16, *p* < 0.0001, *n* = 47), when the food source was live OP50 *E. coli* (*D* = 1.10, 1.05–1.14, *FDR* <0.0001, *n* = 79), the use of FUdR (*D* = 1.10, 1.06–1.15, *FDR* <0.0001, *n* = 73), and in cases of early onset (*D* = 1.08, 1.00–1.16, *FDR* = 0.0421, *n* = 36), late onset (*D* = 1.08, 1.04–1.13, *FDR* = 0.0002, *n* = 62), or low doses (*D* = 1.12, 1.08–1.15, *FDR* <0.0001, *n* = 86), cysteine consistently demonstrated a significant prolongation of *C. elegans* survival. However, transgenic nematodes (*D* = 1.04, 0.99–1.11, *FDR* = 0.1499, *n* = 47), the use of dead OP50 *E. coli* as the food source (*D* = 1.01, 0.95–1.07, *FDR* = 0.7082, *n* = 19), and the absence of FUdR (*D* = 1.02, 0.93–1.10, *FDR* = 0.7263, *n* = 25) did not significantly affect nematode survival time. Additionally, high doses (*D* = 0.86, 0.77–0.96, *FDR* = 0.0071, *n* = 12) were found to reduce nematode survival time.

### 3.4 Overall and subgroup analyses for studies in *Drosophila*

Out of 214 *Drosophila* experiments, 86 (40.2%) used *D. melanogaster*, 64 (29.9%) used *D. virilis*, and 64 (29.9%) used *D. kikkawai*. Among these experiments, 114 (53.3%) specifically focused on male *Drosophila*. The “high dose” group for cysteine was defined as concentrations not less than 10 mM, which were used in 53 (24.8%) experiments. Only 2 (0.9%) experiments employed cysteine hydrochloride, while the remaining experiments utilized NAC. To induce oxidative stress, 51 (23.8%) experiments used paraquat, 49 (22.9%) experiments employed starvation, and 50 (23.4%) experiments utilized hyperoxia as environmental stressors. Among all 214 experiments, only 9 (4.2%) involved genetically modified flies.

**FIGURE 4:**
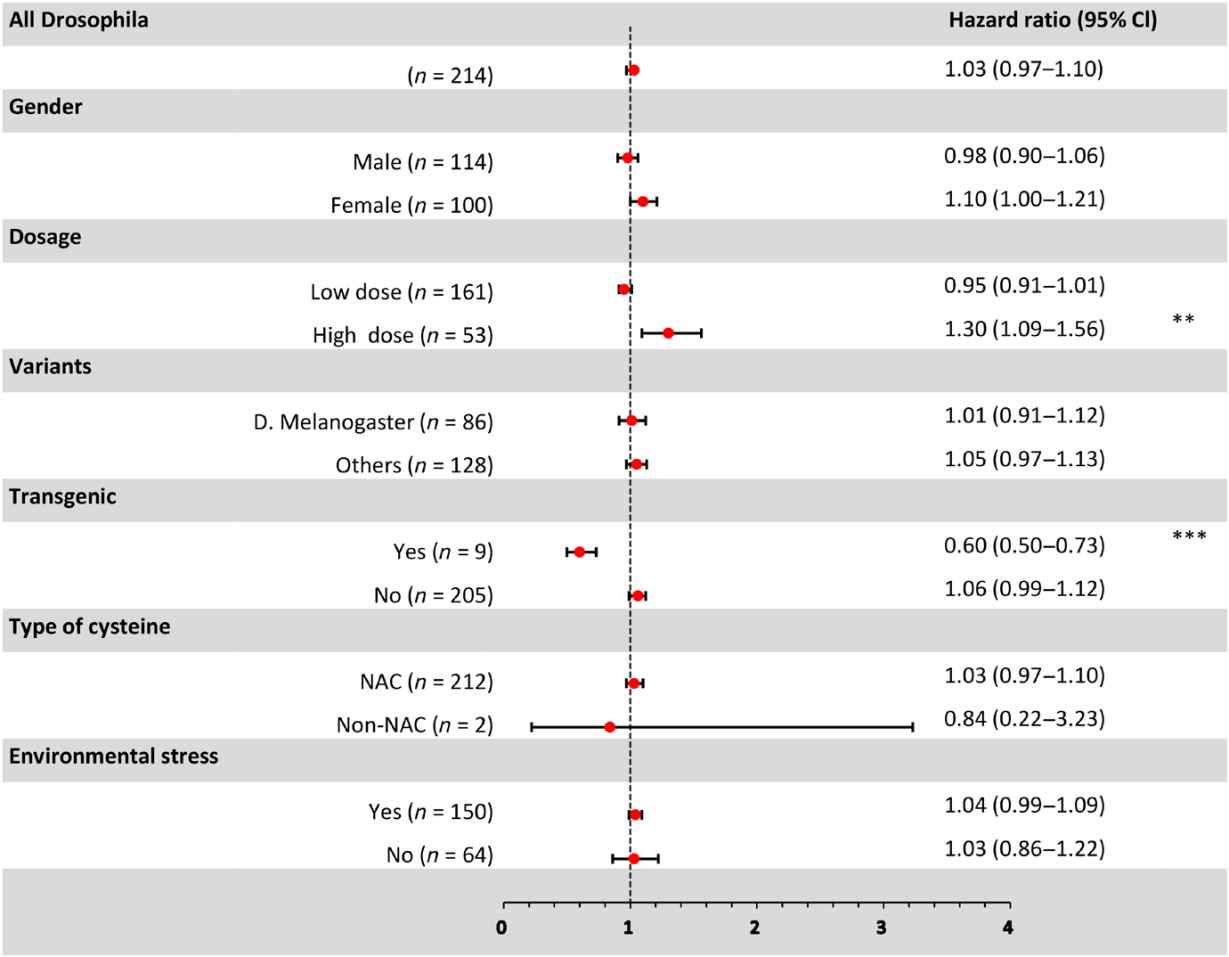
A Forest plot summarizing the meta‐analytic effects for all *Drosophila* experiments and specific subgroups. The left side provides group information, while the right side lists *HRs* and their corresponding 95% CIs. Asterisks indicate levels of statistical significance (**for *p* < 0.01, ***for *p* < 0.001 after the Benjamini‐Hochberg correction for multiple comparisons).

In the combined analysis of all *Drosophila* experiments, cysteine did not show a statistically significant reduction in mortality risk (*HR* = 1.03, 0.97–1.10, *p* = 0.2104, *n* = 214). However, there was significant heterogeneity observed (*I* ^2^ = 95%, 94%–95%, *p* < 0.0001) in the effect (Figure 4 and S5).

Subgroup analysis revealed that cysteine supplementation had a significant positive impact on the survival of transgenic *Drosophila* (*HR* = 0.60, 0.50–0.73, *FDR* = 0.0002, *n* = 9). However, no significant effects were observed in *D. melanogaster* (*HR* = 1.01, 0.91–1.12, *FDR* = 0.8747, *n* = 86), other *Drosophila strains* (*HR* = 1.05, 0.97–1.13, *FDR* = 0.4536, *n* = 128), male (*HR* = 0.98, 0.90–1.06, *FDR* = 0.5407, *n* = 114), female (*HR* = 1.10, 1.00–1.21, *FDR* = 0.0848, *n* = 100), or nontransgenic *Drosophila* (*HR* = 1.06, 0.99–1.12, *FDR* = 0.0828, *n* = 205). Similarly, no significant effects were observed with low dose (*HR* = 0.95, 0.91–1.01, *FDR* = 0.0778, *n* = 161), the use of NAC (*HR* = 1.03, 0.97–1.10, *FDR* = 0.5864, *n* = 212), the use of cysteine hydrochloride (*HR* = 0.84, 0.22–3.23, *FDR* = 0.81, *n* = 2), the presence of induced environmental stress (*HR* = 1.04, 0.99–1.09, *FDR* = 0.2836, *n* = 150), or the absence of environmental stress (*HR* = 1.03, 0.86–1.22, *FDR* = 0.7760, *n* = 64). Interestingly, high‐dose cysteine supplementation increased the risk of death in *Drosophila* (*HR* = 1.30, 1.09–1.56, *FDR* = 0.0082, *n* = 53) (Figure 4).

Meta‐regression analysis revealed that the supplementation of cysteine significantly improved the survival of transgenic flies compared to nontransgenic flies (*HR* = 0.60 vs. 1.06, MR *p* = 0.002) and doses were significantly positively correlated with increases in *HR* (regression coefficient = 0.0032, 0.0014–0.005, MR *p* = 0.0006). There were no significant differences observed based on *Drosophila* strains (*HR* = 1.01 vs. 1.05, MR *p* = 0.5475), gender (*HR* = 0.98 vs. 1.10, MR *p* = 0.0537), type of cysteine used (*HR* = 1.03 vs. 0.84, MR *p* = 0.5485), or whether environmental stress was induced (*HR* = 1.04 vs. 1.03, MR *p* = 0.8301) in their effects on the survival of *Drosophila* (Figure S6).

Combining all experimental results, the analysis of AFT revealed that cysteine supplementation did not have a significant effect on the survival time of fruit flies (*D* = 1.00, 0.97–1.01, *p* = 0.3955, *n* = 214). Cysteine supplementation exhibited a significant positive effect on the survival of transgenic flies (*D* = 1.16, 1.09–1.25, *FDR* = 0.0002, *n* = 9) and high doses significantly reduced survival time (*D* = 0.93, 0.87–0.99, *FDR* = 0.0396, *n* = 53). However, no significant effects were observed in *D. melanogaster* (*D* = 1.00, 0.96–1.03, *FDR* = 0.918, *n* = 86), other *Drosophila* strains (*D* = 0.99, 0.96–1.01, *FDR* = 0.5796, *n* = 128), male flies (*D* = 1.01, 0.98–1.04, *FDR* = 0.4171, *n* = 114), female flies (*D* = 0.97, 0.94–1.00, *FDR* = 0.1166, *n* = 100), and nontransgenic flies (*D* = 0.98, 0.96–1.01, *FDR* = 0.1339, *n* = 205). A lack of effect was also observed when low dose (*D* = 1.01, 0.99–1.03, *FDR* = 0.1977, *n* = 161), NAC (*D* = 0.99, 0.97–1.01, *FDR* = 0.7484, *n* = 212), or cysteine hydrochloride (*D* = 1.04, 0.80–1.35, *FDR* = 0.7597, *n* = 2) was used, as well as in both the presence (*D* = 0.99, 0.97–1.01, *FDR* = 0.5742, *n* = 150) or absence of environmental stress (*D* = 0.99, 0.94–1.05, *FDR* = 0.7576, *n* = 64).

### 3.5 Publication bias and sensitivity analysis

**FIGURE 5:**
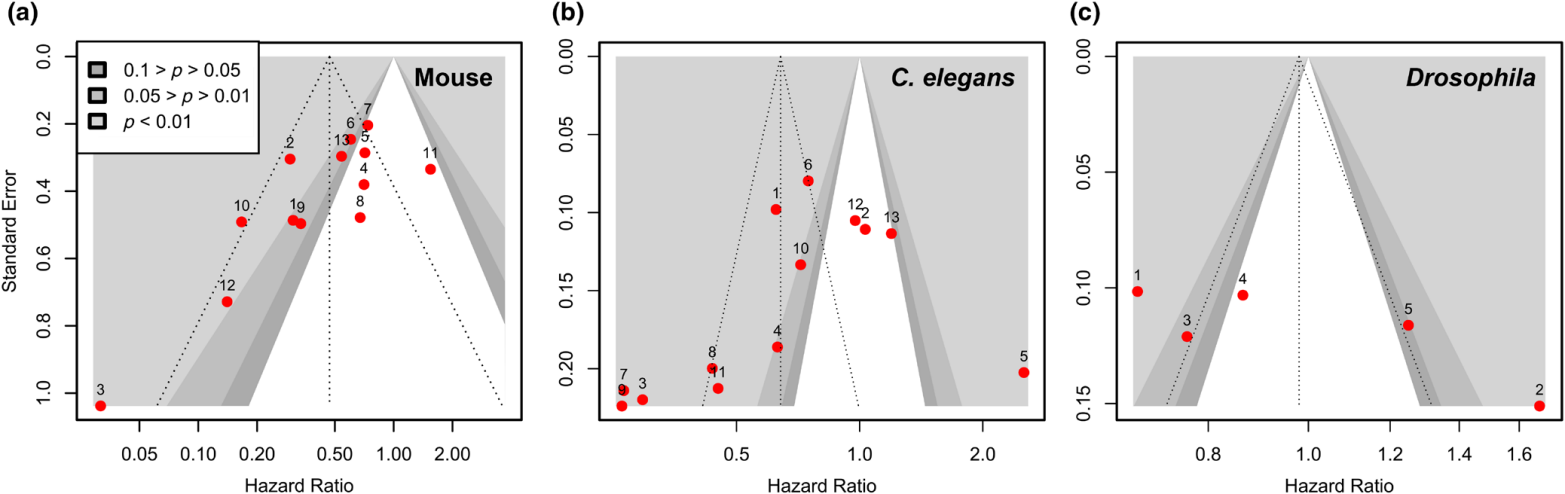
Contour‐enhanced funnel plots for studies in three model organisms. Left, 13 mouse studies; middle, 9 *Caenorhabditis elegans* studies; and right: 5 *Drosophila* studies. Each plot depicts the standard error versus log(*HR*) for the largest experiment within each study. The numbers are the “Study Index” of the associated studies, as listed in Tables S7a,b,c. The shaded region corresponds to indicated *p*‐values and the unshaded region corresponds to *p*‐values greater than 0.1. The vertical lines represent the pooled random effects estimate for each set of experiments, with *HRs* of 0.47 for mice, 0.64 for nematodes, and 0.98 for *Drosophila*. The other dashed lines represent the pseudo 95% confidence limits. Publication bias is evaluated by the statistical significance of the plots determined using Egger's test, with *p*‐values of 0.0181 for mice, 0.14 for nematodes, and 0.12 for *Drosophila*.

We used Galbraith radial plots and Baujat plots to identify the sources of heterogeneity (Baujat et al., 2002; Galbraith, 1988). In mice, it was observed that the six experiments conducted by Harman in 1957 on the C3H mice, Harman in 1961 on the C3H mice and Swiss mice (the above experiments all used high doses of cysteine hydrochloride), Chen et al. in 2020, Kumar et al. in 2022, and Kurano et al. in 2022 had a substantial influence on the overall heterogeneity (Figure S7). When these six experiments were excluded, the overall heterogeneity was significantly reduced (*I* ^*2*^ = 37%, 0%–64%, *p* = 0.0548). In addition, the results of meta‐regression also indicate that differences in dose (regression coefficient = 0.0001, 0–0.0001, MR *p* = 0.0336) may be one of the sources of heterogeneity. Sensitivity analysis indicated that no individual study significantly altered the combined results, and the *HR* ranged from 0.48 to 0.52, suggesting that our meta‐analysis findings are robust and reliable (Figure S8). The funnel plot and Egger's test (*p* = 0.0181) revealed significant publication bias (Figure 5a). Using the trim and fill method, 3 studies were identified as outliers to the right of the mean. And the results from the random effects model still demonstrated an improvement in mice survival with cysteine treatment (*HR* = 0.53, 0.35–0.81, *p* < 0.0001, *n* = 15) (Figure S9).

For nematodes, the two high‐dose groups in Gusarov et al.'s, 2021 study and two in Yang & Hekimi's, 2010 study may contribute to the observed heterogeneity (Figure S10). The results of the meta‐regression analysis examining the effect of dose (*HR* = 0.61 vs. 1.19, MR *p* = 0.0004) and the utilization of FUdR (*HR* = 0.63 vs. 0.99, MR *p* = 0.0119) suggest that varying doses and the decision to administer FUdR may contribute to the observed heterogeneity. Sensitivity analyses demonstrated that none of the individual experiments substantially altered the overall result, and the *HR* ranged from 0.69 to 0.71 (Figure S11). Findings from the funnel plot and Egger's test (*p* = 0.1353) showed no significant publication bias present (Figure 5b).

For the *Drosophila studies*, it is evident that heterogeneity is not solely attributed to a few specific experiments (Figure S12). When considering the results of the meta‐regression analysis, it indicates that the presence of the transgene (*HR* = 0.60 vs. 1.05, MR *p* < 0.0001) and dose (regression coefficient = 0.0032, 0.0014–0.005, MR *p* = 0.0006) could potentially account for the observed variations, as qualitative influencing factors. Sensitivity analysis demonstrated that none of the experiments significantly influenced the overall results, with the *HR* range falling between 1.02 and 1.04 (Figure S13). Additionally, no apparent publication bias was observed based on the funnel plot and Egger's test (*p* = 0.1246) (Figure 5c).

## 4 DISCUSSION

Numerous preclinical studies conducted on model animals have demonstrated that cysteine levels decrease with age and exert significant impacts on health and lifespan (Kumar et al., 2023; Lapenna, 2023; Martinez‐Banaclocha, 2022; Nakata et al., 1996; Pei et al., 2018). However, conflicting results were also reported and the disparities need to be clarified. While the anti‐aging mechanisms of cysteine are still being unraveled, it is likely that they involve multiple pathways that have organism‐specific effects. The present study aimed to investigate the effects of cysteine supplementation on natural lifespan in model organisms, including mice, *C. elegans*, and *Drosophila*. Our analysis revealed heterogeneous results across the studies, indicating that the effects of cysteine on lifespan can vary and depend on the organism and experimental conditions.

### 4.1 On the mouse studies

In mice, supplementation with cysteine was found to significantly reduce the risk of death and extend survival time. Subgroup analysis revealed that these effects were independent of the dose, administration method, and genetic background (inbred vs. noninbred, transgenic vs. nontransgenic). It should be noted that, however, the AKR and C3H mice used in two studies develop spontaneous cancers and have shorter lifespans (Harman, 1957, 1961). Indeed, most included mouse studies utilized disease models, like amyotrophic lateral sclerosis (Andreassen et al., 2000; Kurano et al., 2022), lymphoma (Reliene & Schiestl, 2006), premature aging (Kondratov et al., 2009), Huntington's disease (Paul et al., 2014), renal tubulointerstitial injury (Jin et al., 2014), senescence‐associated vitamin D deficiency (Chen et al., 2019), diabetes (Frenkel et al., 2019), and pulmonary fibrosis (Chen et al., 2020). All these studies used NAC and found positive effects. In studies using wild‐type animals, no consistently significant improvement was found. Cysteine hydrochloride did not increase the lifespan of male Swiss mice (Harman, 1961) but co‐administration of glycine and NAC prolonged the lifespan of C57BL/6J mice (Kumar et al., 2022). To reduce the risk of inbreeding depression and overgeneralizing strain‐specific findings, Flurkey et al. used the genetically heterogeneous HET3 mice and found that NAC did not affect the lifespan of female mice. The lifespan of male mice was extended but this might be due to diet restriction because reduced food uptake was observed (Flurkey et al., 2010). Using genetically heterogeneous animal models is important in aging studies.

The form of cysteine does affect the outcomes in mice. Cysteine hydrochloride and NAC are both commonly encountered derivatives of cysteine. Despite sharing certain chemical structural similarities, they diverge in terms of usage, as well as human absorption and utilization. Specifically, NAC is an acetylated form of cysteine. Upon oral administration, it is rapidly absorbed in the gut, delivered to the liver, and hydrolyzed to cysteine. This sequence of events ultimately results in a higher rate of oral absorption and utilization for NAC (Dilger & Baker, 2007; Pei et al., 2018) which may partly explain why only the administration of NAC, but not cysteine hydrochloride, impacted lifespan.

### 4.2 On the worm studies

In the case of *C. elegans*, cysteine administration resulted in a significant reduction in mortality risk and an extension of lifespan. Subgroup analysis showed consistent effects regardless of the form of cysteine derivatives or induced environmental stress. However, high doses of cysteine increased the risk of mortality. These results suggest that cysteine can have both beneficial and detrimental effects on the lifespan of *C. elegans*, depending on the dose. This finding is in line with previous research indicating that administering high doses of NAC can potentially disrupt the levels of glutathione in the liver and cause damage to both the liver and kidneys in healthy animals (Liou et al., 2021; Niraula & Kim, 2019). The dose effect also depended on genotypes because NAC reduced the lifespan of the long‐lived *glp‐1* and *clk‐1* mutants (Desjardins et al., 2017; Wei & Kenyon, 2016). In both mutants, levels of ROS are elevated and contribute to their prolonged lifespan. Consistently, Yang & Hekimi showed that elevated superoxide is necessary and sufficient to increase longevity, while Gusarov et al. showed that worms feeding on *E. coli* are overdosed with glutathione and administration of NAC or glutathione inhibited the *skn‐1* pathways and shortened lifespan (Gusarov et al., 2021; Yang & Hekimi, 2010). Therefore, instead of scavenging ROS, modulating a balanced redox state is critical to lifespan extension.

All cysteine derivatives were tested in *C. elegans* and they all extended lifespan. NAC was the most used but both positive (Desjardins et al., 2017; Oh et al., 2015; Oh & Park, 2017; Polyak et al., 2018; Shibamura et al., 2009) and negative (Desjardins et al., 2017; Gusarov et al., 2021; Wei & Kenyon, 2016; Yang & Hekimi, 2010) effects were reported and the discrepancy could not be explained by the dose difference. NAC's impact on lifespan depends on *daf‐16* but whether it depends on *skn‐1* is contradictory (Gusarov et al., 2021; Oh & Park, 2017; Wei & Kenyon, 2016). Selencysteine, SAC, and SAMC were reported to extend lifespan (Kim et al., 2017; Ogawa et al., 2016) but a lack of effect was also reported for a higher dose of SAC (Kim & Park, 2016). They all depend on *skn‐1* but not *daf‐16* (Kim et al., 2018; Ogawa et al., 2016). The differential dependencies on the anti‐aging pathways suggested that these cysteine derivatives may function through different mechanisms.

### 4.3 On the *Drosophila* studies

In *Drosophila*, the overall impact of cysteine supplementation on lifespan was found to be statistically nonsignificant. Subgroup analysis revealed a significant positive effect on the survival of short‐lived transgenic flies, including the oxidative stress‐sensitive frataxin‐deficient flies and flies lacking the lysosomal cystine transporter cystinosin (Jouandin et al., 2022; Russi et al., 2020). All studies using wildtype and white mutant flies reported negative effects with high doses (10 mM or higher) of NAC (Brack et al., 1997; Niraula & Kim, 2019; Shaposhnikov et al., 2018). At lower concentrations, NAC exhibited effects in a species‐ and gender‐dependent manner. Such a dose effect was also observed in mice and worms, highlighting the importance of dose optimization. Shaposhnikov et al. comprehensively studied the impacts of NAC using three *Drosophila* species and 8 NAC concentrations. They found that 1 mM or less NAC improved the lifespan of all male flies and female *D. virilis*, but reduced the lifespan of female *D. melanogaster* (Shaposhnikov et al., 2018). For a *D. melanogaster* white mutant, however, improved lifespan was only observed in the female (Niraula & Kim, 2019). A lack of general life extension effect of NAC is consistent with previous findings that NAC has limited in vivo antioxidant effects in flies (Albrecht et al., 2011).

### 4.4 Uncertainties on the underlying molecular mechanism

The mechanisms underlying the positive effects of cysteine supplementation on lifespan remain incompletely understood. The functions of cysteine are mainly due to its roles as an antioxidant, a metabolic precursor, and a regulator of various biological pathways (summarized in Figure S14). For mechanistic details and further discussion on biological activity and application of cysteine (Bonifácio et al., 2021; Pei et al., 2018), NAC (Tenório et al., 2021), and glutathione (Lapenna, 2023), please refer to the respective reviews.

Because of the free radical theory of aging (López‐Otín et al., 2023; Ristow & Zarse, 2010), many attribute the effects of NAC to its antioxidant properties. However, its direct antioxidant capacity is low under physiological conditions (Tenório et al., 2021). NAC may be converted to cysteine and serve as an essential precursor to glutathione, a major antioxidant that safeguards cells against oxidative stress. However, increased longevity was observed in worms treated with 1 μM SAC and male *Drosophila* treated with 10 nM NAC (Ogawa et al., 2016; Shaposhnikov et al., 2018). Such low concentrations of cysteine derivatives are unlikely to significantly change the cysteine concentration and the redox state in the body, suggesting that they may function through additional pathways.

Cysteine is involved in metabolism and many cellular pathways (Bonifácio et al., 2021; Dong et al., 2018). It regulates its own metabolism, the tricarboxylic acid cycle, TORC1 activation, and autophagy (Jouandin et al., 2022). Cysteine is a proteinogenic amino acid, and oxidative stress can lead to the irreversible trioxidation of cysteine residues in proteins. This modification accumulates in the aging skin and lung proteomes, as well as in the platelet fractions of elderly plasma (Sánchez Milán, Fernández‐Rhodes, et al., 2024; Sánchez Milán, Mulet, et al., 2024). Trioxidized cysteine residues may contribute to aging by mimicking phosphoserine and interfering with signaling pathways (Sánchez Milán, Fernández‐Rhodes, et al., 2024). Cysteine is a precursor to multiple metabolites. Notably, it is a critical source of H_2_S, a signaling molecule involved in life extension (Wei & Kenyon, 2016), and taurine, whose levels decrease with age. Taurine extends lifespan and improves multiple health markers and age‐related characteristics in mice (Singh et al., 2023). However, because low concentrations of NAC may not significantly alter cysteine levels, its effects cannot be solely attributed to cysteine's role as a metabolic precursor. This is especially true for taurine, which is present in high levels in human plasma. (~100 μM, Wright et al., 1986).

Cysteine derivatives exhibit broad regulatory functions. NAC has anti‐inflammatory activities and can inhibit the activation of nuclear factor‐κB (NF‐κB), a protein complex that plays a key role in regulating the immune response. It was also reported to modulate other cellular processes, such as altering the microtubule cytoskeleton (Ouyang et al., 2019; Piermarini et al., 2016), regulating the neurotransmitter systems, and participating in signal pathways (Devrim‐Lanpir et al., 2021; Pei et al., 2018). SAC binds to and suppresses calpain to protect cells against endoplasmic reticulum stress (Kosuge, 2020). Dietary cysteine may also affect food uptake (Flurkey et al., 2010; Song et al., 2023).

Therefore, cysteine supplementation has a large range and sometimes opposing effects that may be dependent on or independent of its antioxidative properties. Elucidating the major aging pathways affected by cysteine and the interactions between these pathways are essential for our understanding of the molecular mechanism of cysteine and the clinical application of cysteine derivatives.

### 4.5 Uncertainties on the clinical use of cysteine derivatives

In preclinical and clinical studies, NAC was found to improve sports performance and benefit patients with multiple diseases (Devrim‐Lanpir et al., 2021; Tenório et al., 2021). However, unfavorable effects, like renal and hepatic necrosis and pulmonary congestion, were reported and NAC constantly exhibited toxic effects at high doses in various model animals (Dong et al., 2018; Pei et al., 2018). In humans, oral NAC supplementation is known to cause side effects, such as nausea, vomiting, and diarrhea, especially with chronic intakes (Braakhuis & Hopkins, 2015; Devrim‐Lanpir et al., 2021). Therefore, further studies are necessary to optimize the dosage of NAC across different disease/genetic backgrounds.

Dietary cysteine was shown to eliminate many of the beneficial effects of exercise and methionine restriction in mammals (Dong et al., 2018; Petersen et al., 2012). Similar to diet restriction, methionine restriction extends healthspan and lifespan in various organisms. Cysteine metabolism and methionine metabolism are closely linked and dietary cysteine reduces the requirement of dietary methionine (Dong et al., 2018). Cysteine supplements may therefore exert adverse effects on healthspan through altering methionine metabolism or other metabolism pathways (Jouandin et al., 2022), and should be avoided in this context.

ROS are fundamentally important signaling molecules and play an important role in lifespan extension. In *C. elegans*, ROS is necessary to extend the lifespan of some mutants. NAC shortened the lifespan and paraquat and other pro‐oxidant molecules extended the lifespan of worms (Desjardins et al., 2017; Gusarov et al., 2021; Wei & Kenyon, 2016; Yang & Hekimi, 2010). Paradoxically, antioxidants like NAC can induce oxidative stress (Childs et al., 2001; Mlejnek et al., 2021). In most clinical trials, long‐term antioxidant supplements failed to show health benefits (Schmidt et al., 2015). To date, there is only one open‐label clinical trial that has studied the effect of NAC from the aspect of aging. The study was conducted in eight aged adults and found that co‐administration of glycine and NAC improved glutathione levels, muscle strength, cognition, and other parameters (Kumar et al., 2021). Large‐scale studies with subjects of heterogeneous genetic backgrounds are needed.

### 4.6 Limitations

While our study provides valuable insights into the effects of cysteine supplementation on lifespan in these animal models, there are certain limitations in our research.

First, our search process was limited to three databases and excluded unpublished preprints. Despite our efforts to include additional studies from the references and gray literature on Google Scholar, it is possible that some literature, especially those published in nonEnglish languages were inadvertently excluded. Second, the CAMARADES quality scores of the included studies are generally low (1–5, out of a maximum of 9, Table S3). Notably, none of the experiments implemented blinding or allocation concealment. This lack of methodological rigor could potentially undermine the reliability of our conclusions. Third, despite our efforts to acquire raw data from the authors, we were unable to obtain the requested information. Consequently, we were compelled to rely solely on reconstructed survival data. This methodology inevitably introduces some degree of noise. However, the procedure was independently performed by two authors, and their results showed close agreement. Lastly, although no significant publication bias was observed in the studies conducted on *Drosophila* (Figure 5), this absence of bias could be attributed to the limited number of included studies.

## 5 CONCLUSION AND FUTURE PROSPECTIVE

Our study represents the first meta‐analysis conducted to quantify the impact of cysteine supplementation on lifespan in three distinct experimental animal models: mice, nematodes, and fruit flies. Our analysis demonstrates that cysteine supplementation has a significant impact on lifespan in disease mouse models and *C. elegans*, but not in nontransgenic fruit flies. These findings suggest that cysteine supplementation has potential anti‐aging effects in certain model organisms, especially in the context of diseases. Further research is needed to fully understand the mechanisms underlying cysteine's impact on lifespan and to determine optimal dosages and administration methods for potential anti‐aging interventions using cysteine.

## AUTHOR CONTRIBUTIONS

Yue Ma developed and designed the analysis, conducted data collection, performed the analysis, and wrote and edited the manuscript. Mengqi Chen gathered data and performed the analysis. Wakam Chang supervised the project, verified the search and analytical results, and wrote and edited the manuscript. Kaiyao Huang discussed the results and contributed to the final manuscript.

## CONFLICT OF INTEREST STATEMENT

None declared.

## Supporting information


Data S1.

## Data Availability

All the data used in this study will be made publicly available through the Open Science Framework (osf.io) upon publication. I confirm that I have included a citation for available data in my references section.
