# Supplementary material for: The impact of cysteine on lifespan in three model organisms: A systematic review and meta‐analysis
Source: Aging Cell. 2024 Oct 30;24(2):e14392. doi: 10.1111/acel.14392 (PMC11822635; doi:10.1111/acel.14392)
Supplement: Supplementary file 1 — Data S1. [file ACEL-24-e14392-s001.zip › Supplementary.pdf]

List of supplementary materials

Table S1. PRISMA 2020 Checklist 2
Table S2. Search strategies. 4
Table S3. CAMARADES quality scores for each included study. 5
Table S4. Characteristics of 25 mouse lifespan experiments included in this review, from 13 published studies. 6
Table S5. Characteristics of 98 *C. elegans* lifespan experiments included in this review, from 13 published studies. 8
Table S6. Characteristics of 214 *Drosophila* lifespan experiments included in this review, from 5 published studies. 12
Table S7a. Thirteen mouse lifespan studies included in the funnel plot. 17
Table S7b. Thirteen *C. elegans* lifespan studies included in the funnel plot. 18
Table S7c. Five *Drosophila* lifespan studies included in the funnel plot. 19
Figure S1. PRISMA flow diagram for the review. 20
Figure S2. Random-effects meta-regression plots of the association between experimental conditions and *HR* for all mice experiments. 21
Figure S3. Forest plot of hazard ratios for all *C. elegans* experiments. 23
Figure S4. Random-effects meta-regression plots of the association between experimental conditions and *HR* for all *C. elegans* experiment 24
Figure S5. Forest plot of hazard ratios for all *Drosophila* experiments. 27
Figure S6. Random-effects meta-regression plots of the association between experimental conditions and HR for all *Drosophila* experiments. 28
Figure S7. Galbraith plot (A) and Baujat plot (B) for all mice experiments. 29
Figure S8. Sensitivity analysis using leave-one-out procedure for all mice experiments. 30
Figure S9. Trim and fill methods to identify and correct asymmetries in funnel plots. 31
Figure S10. Galbraith plot (A) and Baujat plot (B) for all *C. elegans* experiments. 32
Figure S11. Sensitivity analysis using leave-one-out procedure for all *C. elegans* experiments. 34
Figure S12. Galbraith plot (A) and Baujat plot (B) for all *Drosophila* experiments. 35
Figure S13. Sensitivity analysis using leave-one-out procedure for all *Drosophila* experiments. 39
Figure S14. Mechanisms of action of cysteine. 40

Table S1. PRISMA 2020 Checklist

Section and Topic	Item #	Checklist item	Location where item is reported
**TITLE**			
Title	1	Identify the report as a systematic review.	1
**ABSTRACT**			
Abstract	2	See the PRISMA 2020 for Abstracts checklist.	2
**INTRODUCTION**			
Rationale	3	Describe the rationale for the review in the context of existing knowledge.	3-5
Objectives	4	Provide an explicit statement of the objective(s) or question(s) the review addresses.	3-5
**METHODS**			
Eligibility criteria	5	Specify the inclusion and exclusion criteria for the review and how studies were grouped for the syntheses.	6
Information sources	6	Specify all databases, registers, websites, organisations, reference lists and other sources searched or consulted to identify studies. Specify the date when each source was last searched or consulted.	6
Search strategy	7	Present the full search strategies for all databases, registers and websites, including any filters and limits used.	6
Selection process	8	Specify the methods used to decide whether a study met the inclusion criteria of the review, including how many reviewers screened each record and each report retrieved, whether they worked independently, and if applicable, details of automation tools used in the process.	6
Data collection process	9	Specify the methods used to collect data from reports, including how many reviewers collected data from each report, whether they worked independently, any processes for obtaining or confirming data from study investigators, and if applicable, details of automation tools used in the process.	6-7
Data items	10a	List and define all outcomes for which data were sought. Specify whether all results that were compatible with each outcome domain in each study were sought (e.g. for all measures, time points, analyses), and if not, the methods used to decide which results to collect.	6
	10b	List and define all other variables for which data were sought (e.g. participant and intervention characteristics, funding sources). Describe any assumptions made about any missing or unclear information.	6
Study risk of bias assessment	11	Specify the methods used to assess risk of bias in the included studies, including details of the tool(s) used, how many reviewers assessed each study and whether they worked independently, and if applicable, details of automation tools used in the process.	7
Effect measures	12	Specify for each outcome the effect measure(s) (e.g. risk ratio, mean difference) used in the synthesis or presentation of results.	6-7
Synthesis methods	13a	Describe the processes used to decide which studies were eligible for each synthesis (e.g. tabulating the study intervention characteristics and comparing against the planned groups for each synthesis (item #5)).	6
	13b	Describe any methods required to prepare the data for presentation or synthesis, such as handling of missing summary statistics, or data conversions.	6-7
	13c	Describe any methods used to tabulate or visually display results of individual studies and syntheses.	6-7
	13d	Describe any methods used to synthesize results and provide a rationale for the choice(s). If meta-analysis was performed, describe the model(s), method(s) to identify the presence and extent of statistical heterogeneity, and software package(s) used.	7
	13e	Describe any methods used to explore possible causes of heterogeneity among study results (e.g. subgroup analysis, meta-regression).	7
	13f	Describe any sensitivity analyses conducted to assess robustness of the synthesized results.	7
Reporting bias	14	Describe any methods used to assess risk of bias due to missing results in a synthesis (arising from reporting	7

Section and Topic	Item #	Checklist item	Location where item is reported
assessment		biases).	
Certainty assessment	15	Describe any methods used to assess certainty (or confidence) in the body of evidence for an outcome.	6
**RESULTS**			
Study selection	16a	Describe the results of the search and selection process, from the number of records identified in the search to the number of studies included in the review, ideally using a flow diagram.	8, Figure S1
	16b	Cite studies that might appear to meet the inclusion criteria, but which were excluded, and explain why they were excluded.	8, Figure S1
Study characteristics	17	Cite each included study and present its characteristics.	Table S7
Risk of bias in studies	18	Present assessments of risk of bias for each included study.	13-14, Figure 5
Results of individual studies	19	For all outcomes, present, for each study: (a) summary statistics for each group (where appropriate) and (b) an effect estimate and its precision (e.g. confidence/credible interval), ideally using structured tables or plots.	8-13, Figure 2-4
Results of syntheses	20a	For each synthesis, briefly summarise the characteristics and risk of bias among contributing studies.	8-13, Figure 2-4
	20b	Present results of all statistical syntheses conducted. If meta-analysis was done, present for each the summary estimate and its precision (e.g. confidence/credible interval) and measures of statistical heterogeneity. If comparing groups, describe the direction of the effect.	8-13, Figure 2-4
	20c	Present results of all investigations of possible causes of heterogeneity among study results.	9-12, Figure S7,10,12
	20d	Present results of all sensitivity analyses conducted to assess the robustness of the synthesized results.	13, Figure S8,11,13
Reporting biases	21	Present assessments of risk of bias due to missing results (arising from reporting biases) for each synthesis assessed.	13, Figure S9
Certainty of evidence	22	Present assessments of certainty (or confidence) in the body of evidence for each outcome assessed.	7-13
**DISCUSSION**			
Discussion	23a	Provide a general interpretation of the results in the context of other evidence.	14-17
	23b	Discuss any limitations of the evidence included in the review.	17-18
	23c	Discuss any limitations of the review processes used.	17-18
	23d	Discuss implications of the results for practice, policy, and future research.	17-18
**OTHER INFORMATION**			
Registration and protocol	24a	Provide registration information for the review, including register name and registration number, or state that the review was not registered.	6
	24b	Indicate where the review protocol can be accessed, or state that a protocol was not prepared.	6
	24c	Describe and explain any amendments to information provided at registration or in the protocol.	6
Support	25	Describe sources of financial or non-financial support for the review, and the role of the funders or sponsors in the review.	19
Competing interests	26	Declare any competing interests of review authors.	19
Availability of data, code and other materials	27	Report which of the following are publicly available and where they can be found: template data collection forms; data extracted from included studies; data used for all analyses; analytic code; any other materials used in the review.	19

From: Page MJ, McKenzie JE, Bossuyt PM, Boutron I, Hoffmann TC, Mulrow CD, et al. The PRISMA 2020 statement: an updated guideline for reporting systematic reviews. BMJ 2021;372:n71. doi: 10.1136/bmj.n71

For more information, visit: http://www.prisma-statement.org/

Table S2. Search strategies.

Database	Search keyword
PubMed	(cysteine*[Title] OR "N--acetyl cysteine"[Title] OR "N-acetyl-L-cysteine"[Title] OR NAC[Title]) AND ((lifespan[Title] OR "life span"[Title] OR "life-span"[Title] OR survival[Title] OR healthspan[Title/Abstract] OR "health-span"[Title/Abstract] OR longevity[Title/Abstract] OR aging[Title/Abstract] OR ageing[Title/Abstract]) OR aging[majr])
Web of Science	TI = ((cysteine* OR "N--acetyl cysteine" OR "N-acetyl-L-cysteine" OR NAC*) AND (lifespan OR "life span" OR "life-span" OR survival OR healthspan OR "health-span" OR longevity OR aging OR ageing OR senescen*)).
Google Scholar	allintitle: (cysteine* OR "N--acetyl cysteine" OR "N-acetyl-L-cysteine" OR NAC*) AND (lifespan OR "life span" OR "life-span" OR longevity OR aging OR ageing OR healthspan OR "health span" OR "healthspan" OR "survival")

Table S3. CAMARADES quality scores for each included study.

The total quality scores have a possible maximum of 9.

Study	PubMed PMID	Organism	Total quality score (out of 9)	Peer-reviewed publication	Statement of control of temperature	Randomization of treatment	Allocation concealment	Blinded assessment of outcomes	Blinded application of treatment	Sample size calculation	Compliance statement	Statement of conflict of interest
Andreassen, 2000	10943709	Mice	2	Y	N	N	N	N	N	N	Y	N
Chen, 2019	30907059	Mice	4	Y	N	Y	N	N	N	N	Y	Y
Chen, 2020	31959867	Mice	4	Y	N	Y	N	N	N	N	Y	Y
Flurkey, 2010	20819793	Mice	4	Y	Y	N	N	N	N	Y	Y	N
Frenkel, 2019	30993256	Mice	5	Y	Y	N	N	N	N	Y	Y	Y
Harman, 1957	13463294	Mice	1	N	N	Y	N	N	N	N	N	N
Harman, 1961	13711616	Mice	1	N	N	Y	N	N	N	N	N	N
Jin, 2014	24915841	Mice	4	Y	N	Y	N	N	N	N	Y	Y
Kondratov, 2009	20157581	Mice	3	Y	N	N	N	N	N	N	Y	Y
Kurano, 2022	36559085	Mice	5	Y	Y	Y	N	N	N	N	Y	Y
Kumar, 2022	35268089	Mice	3	Y	N	N	N	N	N	N	Y	Y
Paul, 2014	24670645	Mice	5	Y	N	Y	N	N	N	Y	Y	Y
Reliene, 2006	16781197	Mice	2	Y	N	N	N	N	N	N	Y	N
Desjardins, 2017	27683245	*C. elegans*	5	Y	Y	Y	N	N	N	N	Y	Y
Gusarov, 2021	34267196	*C. elegans*	4	Y	Y	N	N	N	N	N	Y	Y
Kim, 2016	28954009	*C. elegans*	3	Y	Y	Y	N	N	N	N	N	N
Kim, 2017	*	*C. elegans*	4	Y	Y	Y	N	N	N	N	N	Y
Kim, 2018	30365103	*C. elegans*	4	Y	Y	Y	N	N	N	N	N	Y
Ogawa, 2016	26899496	*C. elegans*	5	Y	Y	Y	N	N	N	N	Y	Y
Oh, 2015	26039957	*C. elegans*	4	Y	Y	Y	N	N	N	N	N	Y
Oh, 2017	30263604	*C. elegans*	4	Y	Y	Y	N	N	N	N	N	Y
Polyak, 2018	29526616	*C. elegans*	4	Y	Y	Y	N	N	N	N	N	Y
Savion, 2018	29579097	*C. elegans*	4	Y	Y	Y	N	N	N	N	N	Y
Shibamura, 2009	19580823	*C. elegans*	2	Y	N	Y	N	N	N	N	N	N
Wei, 2016	27140632	*C. elegans*	4	Y	Y	Y	N	N	N	N	N	Y
Yang, 2010	21151885	*C. elegans*	4	Y	Y	Y	N	N	N	N	N	Y
Brack, 1997	9447249	*D. melanogaster*	3	Y	Y	Y	N	N	N	N	N	N
Jouandin, 2022	35175796	*D. melanogaster*	5	Y	Y	Y	N	N	N	N	Y	Y
Niraula, 2019	31115735	*D. melanogaster*	3	Y	Y	Y	N	N	N	N	N	N
Russi, 2020	32744307	*D. melanogaster*	3	Y	N	Y	N	N	N	N	N	Y
Shaposhnikov, 2018	30243020	*D. melanogaster*<br>*D. virilis*<br>*D. kikkawai*	5	Y	Y	Y	N	N	N	N	Y	Y

* PubMed ID not available. doi: 10.14393/BJ-v33n2-32846

Table S4. Characteristics of 25 mouse lifespan experiments included in this review, from 13 published studies.

Study	Experiment index	Animal variant (inbred or non-inbred)	Number of mice in the control group	Number of mice in the experiment group	Gender of mice	Age that cysteine was started at (weeks)	Delivery	Type of cysteine	Cysteine dose	Other experimental characteristics
Andreassen, 2020	1	B6SJL (Outbred)	18	12	Male	4-5	Drinking water	NAC	2.0 mg/kg/d	G93A transgenic
Chen, 2019	2	BALB/c (Inbred)	25	25	Unspecified	4	Drinking water	NAC	1 mg/mL	Vitamin D receptor (Vdr)$^{-/-}$ mice
Chen, 2020	3	129Ola/FVB/N (Hybrid)	46	8	Unspecified	4-5	Drinking water	NAC	1 mg/mL	Bmi-1$^{-/-}$
Flurkey, 2010	4	UM-HET3 (Outbred)	16	16	Female	28	Drinking water	NAC	1.2 g/kg/d	
	5	UM-HET3 (Outbred)	16	16	Female	28	Drinking water	NAC	0.6 g/kg/d	
	6	UM-HET3 (Outbred)	16	16	Male	28	Drinking water	NAC	1.2 g/kg/d	
	7	UM-HET3 (Outbred)	16	16	Male	28	Drinking water	NAC	0.6 g/kg/d	
Frenkel, 2019	8	NOD (Outbred)	40	41	Female	6	Drinking water	NAC	0.6 g/kg/d	Overexpression of catalase, sensitized nonobese diabetic
	9	NOD (Outbred)	40	41	Female	6	Drinking water	NAC	0.6 g/kg/d	Offspring of NAC-treated mice
Harman, 1957	10	AKR (Outbred)	56	30	Male	After weaning	Chow	Cys-HCl	1% weight	
	11	AKR (Outbred)	56	30	Male	After weaning	Chow	Cys-HCl	0.5% weight	
	12	C3H (Outbred)	247	30	Female	After weaning	Chow	Cys-HCl	1% weight	
	13	C3H (Outbred)	247	30	Female	After weaning	Chow	Cys-HCl	0.5% weight	
Harman, 1961	14	AKR (Outbred)	60	47	Male	After weaning	Chow	Cys-HCl	1% weight	
	15	C3H (Outbred)	92	49	Female	After weaning	Chow	Cys-HCl	1% weight	
	16	Swiss (Outbred)	60	46	Male	After weaning	Chow	Cys-HCl	1% weight	
Jin, 2014	17	129Ola/FVB/N (Hybrid)	19	6	Unspecified	4-5	Drinking water	NAC	1 mg/ mL	Bmi-1$^{-/-}$
Kondratov, 2009	18	C57BL/6J (Inbred)	10	10	Unspecified	4-5	Drinking water	NAC	40 mM (6.5 mg/mL)	Bmi-1$^{-/-}$
Kumar, 2022	19	C57BL/6J (Outbrid)	16	16	Unspecified	65	Chow	GlyNAC	1.6 g/kg/d	
kurano, 2022	20	B6SJL (Outbred)	20	20	Male	15	intraperitoneal	NAC	1 mg	G93A transgenic
	21	B6SJL (Outbred)	20	16	Male	15	intranasal	NAC	1 mg	G93A transgenic
	22	B6SJL (Outbred)	19	17	Male	15	intranasal	NAC	0.2 mg	G93A transgenic, intranasal administration combined

										with PEG-PCL-Tat (a nanocarrier)
	23	B6SJL (Outbred)	19	16	Male	15	intranasal	NAC	1 mg	G93A transgenic, intranasal administration combined with PEG-PCL-Tat (a nanocarrier)
Paul, 2014	24	B6CBA(Outbred)	7	7	Unspecified	4	Drinking water	NAC	20 mM (3.3 mg/mL)	R6/2 (B6CBA-Tg(HDexon1) 62Gpb/1J) transgenic
Reliene, 2006	25	C57BL/6J (Inbred)	34	22	Unspecified	After weaning	Drinking water	NAC	1 g/kg/d	$ATM^{-/-}$

NAC: N-acetyl cysteine. GlyNAC: glycine and N-acetyl cysteine. Cys-HCl: Cysteine hydrochloride.

Table S5. Characteristics of 98 *C. elegans* lifespan experiments included in this review, from 13 published studies.

Study	Experiment index	*C. elegans* variant	*E. coli* food source	Number of worms in control group	Number of worms in experimental group	Type of cysteine	Cysteine dosage	Starting age/Stage	FUdR used for progeny prevention	Other experimental characteristics
Desjardins, 2017	1	N2 WT	OP50 (live)	255	222	NAC	3 mM	L1	Yes	
	2	N2 WT	OP50 (live)	255	226	NAC	6 mM	L1	Yes	
	3	N2 WT	OP50 (live)	255	226	NAC	9 mM	L1	Yes	
	4	N2 *bus-8(e2698)* mutant	OP50 (live)	121	124	NAC	3 mM	L1	Yes	*bus-8(e2698)* mutant
	5	N2 *bus-8(e2698)* mutant	OP50 (live)	121	127	NAC	6 mM	L1	Yes	*bus-8(e2698)* mutant
	6	N2 *bus-8(e2698)* mutant	OP50 (live)	121	122	NAC	9 mM	L1	Yes	*bus-8(e2698)* mutant
	7	N2 *clk-1(qm30)* mutant	OP50 (live)	137	135	NAC	3 mM	L1	Yes	*clk-1(qm30)* mutant
	8	N2 *clk-1(qm30)* mutant	OP50 (live)	137	130	NAC	6 mM	L1	Yes	*clk-1(qm30)* mutant
	9	N2 *clk-1(qm30)* mutant	OP50 (live)	137	132	NAC	9 mM	L1	Yes	*clk-1(qm30)* mutant
	10	N2 *bus-8; clk-1* mutant	OP50 (live)	136	135	NAC	3 mM	L1	Yes	*bus-8; clk-1* mutant
	11	N2 *bus-8; clk-1* mutant	OP50 (live)	136	131	NAC	6 mM	L1	Yes	*bus-8; clk-1* mutant
	12	N2 *bus-8; clk-1* mutant	OP50 (live)	136	133	NAC	9 mM	L1	Yes	*bus-8; clk-1* mutant
	13	N2 *isp-1(qm150)* mutant	OP50 (live)	127	132	NAC	3 mM	L1	Yes	*isp-1(qm150)* mutant
	14	N2 *isp-1(qm150)* mutant	OP50 (live)	127	132	NAC	6 mM	L1	Yes	*isp-1(qm150)* mutant
	15	N2 *isp-1(qm150)* mutant	OP50 (live)	127	128	NAC	9 mM	L1	Yes	*isp-1(qm150)* mutant
	16	N2 *sod-12345* mutant	OP50 (live)	100	100	NAC	3 mM	L1	Yes	*sod-1(tm783),sod-2(ok1030), sod-3(tm760), sod-4(gk101), and sod-5(tm1246)* mutant
	17	N2 *sod-12345* mutant	OP50 (live)	100	100	NAC	6 mM	L1	Yes	*sod-1(tm783),sod-2(ok1030), sod-3(tm760), sod-4(gk101), and sod-5(tm1246)* mutant
	18	N2 *sod-12345* mutant	OP50 (live)	100	100	NAC	9 mM	L1	Yes	*sod-1(tm783),sod-2(ok1030), sod-3(tm760), sod-4(gk101), and sod-5(tm1246)* mutant
Gusarov, 2021	19	N2 Bristol WT	OP50 (live)	234	159	NAC	5 mM	L4	No	
	20	N2 Bristol WT	OP50 (live)	234	223	NAC	15 mM	L4	No	
	21	N2 Bristol WT	OP50 (dead)	525	263	NAC	5 mM	L4	No	
	22	N2 Bristol WT	OP50 (dead)	525	211	NAC	15 mM	L4	No	
	23	N2 Bristol WT	OP50 (dead)	261	289	NAC	5 mM	13 days	No	
Kim, 2016	24	N2 CGCb WT	OP50 (live)	60	60	SAC	5 µM	3 days	No	H_2O_2 oxidative stress

	25	N2 CGCb WT	OP50 (live)	60	60	SAC	5 µM	3 days	No	Heat stress
	26	N2 CGCb WT	OP50 (live)	60	60	SAC	5 µM	3 days	Yes	
Kim, 2017	27	N2 CGCb WT	OP50 (live)	60	60	selenocysteine	1 mM	3 days	No	UV irradiation
	28	N2 CGCb WT	OP50 (live)	60	60	selenocysteine	2.5 mM	3 days	No	UV irradiation
	29	N2 CGCb WT	OP50 (live)	60	60	selenocysteine	5 mM	3 days	No	UV irradiation
	30	N2 CGCb WT	OP50 (live)	60	60	selenocysteine	1 mM	3 days	No	
	31	N2 CGCb WT	OP50 (live)	60	60	selenocysteine	2.5 mM	3 days	No	
	32	N2 CGCb WT	OP50 (live)	60	60	selenocysteine	5 mM	3 days	Yes	
Kim, 2018	33	N2 CGCb WT	OP50 (live)	60	60	selenocysteine	5 mM	3 days	Yes	
	34	N2 *age-1(hx546)* mutant	OP50 (live)	60	60	selenocysteine	5 mM	3 days	Yes	*age-1(hx546)* mutant
	35	N2 *clk-1(e2519)* mutant	OP50 (live)	60	60	selenocysteine	5 mM	3 days	Yes	*clk-1(e2519)* mutant
	36	N2 *eat-2(ad465)* mutant	OP50 (live)	60	60	selenocysteine	5 mM	3 days	Yes	*eat-2(ad465)* mutant
	37	N2 CGCb WT	OP50(live)	60	60	selenocysteine	5 mM	3 days	Yes	
	38	N2 CGCb WT	Diluted OP50(live)	60	60	selenocysteine	5 mM	3 days	Yes	Dietary restriction
	39	N2 CGCb WT	OP50(live)	60	60	selenocysteine	5 mM	3 days	Yes	Empty vector
	40	N2 *skn-1* RNAi strain	OP50(live)	60	60	selenocysteine	5 mM	3 days	Yes	*skn-1* RNAi strain
	41	N2 CGCb WT	OP50(live)	60	60	selenocysteine	5 mM	3 days	Yes	High glucose
Ogawa, 2016	42	N2 WT	OP50 (dead)	343	295	SAC	1 µM	1 day	Yes	
	43	N2 WT	OP50 (dead)	343	284	SAC	10 µM	1 day	Yes	
	44	N2 WT	OP50 (dead)	343	279	SAC	100 µM	1 day	Yes	
	45	N2 WT	OP50 (dead)	343	272	SAMC	1 µM	1 day	Yes	
	46	N2 WT	OP50 (dead)	343	274	SAMC	10 µM	1 day	Yes	
	47	N2 WT	OP50 (dead)	343	268	SAMC	100 µM	1 day	Yes	
	48	N2 *daf-16(mgDf47)* mutant	OP50 (dead)	190	186	SAC	1 µM	1 day	Yes	*daf-16(mgDf47)* mutant
	49	N2 *daf-16(mgDf47)* mutant	OP50 (dead)	190	185	SAC	10 µM	1 day	Yes	*daf-16(mgDf47)* mutant
	50	N2 *daf-16(mgDf47)* mutant	OP50 (dead)	190	191	SAC	100 µM	1 day	Yes	*daf-16(mgDf47)* mutant
	51	N2 *daf-16(mgDf47)* mutant	OP50 (dead)	190	202	SAMC	1 µM	1 day	Yes	*daf-16(mgDf47)* mutant
	52	N2 *daf-16(mgDf47)* mutant	OP50 (dead)	190	208	SAMC	10 µM	1 day	Yes	*daf-16(mgDf47)* mutant
	53	N2 *daf-16(mgDf47)* mutant	OP50 (dead)	190	202	SAMC	100 µM	1 day	Yes	*daf-16(mgDf47)* mutant
	54	N2 *skn-1(zu67)* mutant	OP50 (dead)	140	135	SAC	10 µM	1 day	Yes	*skn-1(zu67)* mutant
	55	N2 *skn-1(zu67)* mutant	OP50 (dead)	140	142	SAC	100 µM	1 day	Yes	*skn-1(zu67)* mutant
	56	N2 *skn-1(zu67)* mutant	OP50 (dead)	140	137	SAMC	10 µM	1 day	Yes	*skn-1(zu67)* mutant

	57	N2 *skn-1(zu67)* mutant	OP50 (dead)	140	140	SAMC	100 µM	1 day	Yes	*skn-1(zu67)* mutant
Oh, 2015	58	N2 CGCb WT	OP50 (live)	60	60	NAC	1 mM	3 days	Yes	Oxidative stress
	59	N2 CGCb WT	OP50 (live)	60	60	NAC	5 mM	3 days	Yes	Oxidative stress
	60	N2 CGCb WT	OP50 (live)	60	60	NAC	10 mM	3 days	Yes	Oxidative stress
	61	N2 CGCb WT	OP50 (live)	60	60	NAC	50 mM	3 days	Yes	Oxidative stress
	62	N2 CGCb WT	OP50 (live)	60	60	NAC	1 mM	3 days	Yes	
	63	N2 CGCb WT	OP50 (live)	60	60	NAC	2 mM	3 days	Yes	
	64	N2 CGCb WT	OP50 (live)	60	60	NAC	5 mM	3 days	Yes	
Oh, 2017	65	N2 CGCb WT	OP50 (live)	60	60	NAC	5 mM	3 days	Yes	
	66	N2 *age-1(hx546)* mutant	OP50 (live)	60	60	NAC	5 mM	3 days	Yes	*age-1(hx546)* mutant
	67	N2 *clk-1(e2519)* mutant	OP50 (live)	60	60	NAC	5 mM	3 days	Yes	*clk-1(e2519)* mutant
	68	N2 *eat-2(ad465)* mutant	OP50 (live)	60	60	NAC	5 mM	3 days	Yes	*eat-2(ad465)* mutant
	69	N2 CGCb WT	OP50 (live)	60	60	NAC	5 mM	3 days	Yes	Dietary restriction
	70	N2 *skn-1* RNAi strain	OP50(live)	60	60	NAC	5 mM	3 days	Yes	*skn-1* RNAi strain
	71	N2 *daf-16* RNAi strain	OP50(live)	60	60	NAC	5 mM	3 days	Yes	*daf-16* RNAi strain
Polyak, 2018	72	N2 Bristol *gas(fc210)* mutant	OP50 (live)	60	60	NAC	2.5 mM	L1 larval stage	Yes	*gas(fc210)* mutant
	73	N2 Bristol *gas(fc210)* mutant	OP50 (live)	60	60	NAC	2.5 mM	young adult stage	Yes	*gas(fc210)* mutant
Savion, 2018	74	N2 CF512 strain	OP50 (live)	120	120	ASSNAC	0.5 mM	L4 Larva stage	No	
	75	N2 CF512 strain	OP50 (live)	120	120	ASSNAC	2 mM	L4 Larva stage	No	
	76	N2 CF512 strain	OP50 (live)	120	120	ASSNAC	5 mM	L4 Larva stage	No	
Shibamura, 2009	77	N2 Bristol WT	OP50 (live)	50	53	NAC	2.5 µg / 25 µL	3 days	No	Liposome administration
	78	N2 Bristol WT	OP50 (live)	50	53	NAC	5 µg / 25 µL	3 days	No	Liposome administration
	79	N2 Bristol WT	OP50 (live)	50	54	NAC	10 µg / 25 µL	3 days	No	Liposome administration
	80	N2 Bristol WT	OP50 (live)	50	47	NAC	5 µg / 25 µL	3 days	No	
Wei, 2016	81	N2 Bristol WT	OP50 (live)	193	194	NAC	2 mM	L1 larval stage	Yes	
	82	N2 Bristol WT	OP50 (live)	193	191	NAC	4 mM	L1 larval stage	Yes	
	83	N2 Bristol WT	OP50 (live)	193	193	NAC	6 mM	L1 larval stage	Yes	
	84	N2 Bristol WT	OP50 (live)	193	191	NAC	8 mM	L1 larval stage	Yes	
	85	N2 Bristol WT	OP50 (live)	193	177	NAC	10 mM	L1 larval stage	Yes	
	86	N2 *glp-1(ts)* mutant	OP50 (live)	166	184	NAC	2 mM	L1 larval stage	Yes	*glp-1(ts)* mutant
	87	N2 *glp-1(ts)* mutant	OP50 (live)	166	167	NAC	4 mM	L1 larval stage	Yes	*glp-1(ts)* mutant
	88	N2 *glp-1(ts)* mutant	OP50 (live)	166	163	NAC	6 mM	L1 larval stage	Yes	*glp-1(ts)* mutant
	89	N2 *glp-1(ts)* mutant	OP50 (live)	166	174	NAC	8 mM	L1 larval stage	Yes	*glp-1(ts)* mutant
	90	N2 *glp-1(ts)* mutant	OP50 (live)	166	174	NAC	10 mM	L1 larval stage	Yes	*glp-1(ts)* mutant
	91	N2 Bristol WT	OP50 (live)	94	90	NAC	8 mM	1 day	Yes	
	92	N2 *glp-1(ts)* mutant	OP50 (live)	87	91	NAC	8 mM	1 day	Yes	*glp-1(ts)* mutant

Yang, 2010	93	N2 WT	OP50 (live)	400	100	NAC	10 mM	1 day	No	
	94	N2 *nuo-6(qm200)* mutant	OP50 (live)	200	73	NAC	10 mM	1 day	No	*nuo-6(qm200)* mutant
	95	N2 *isp-1(qm150)* mutant	OP50 (live)	200	200	NAC	10 mM	1 day	No	*isp-1(qm150)* mutant
	96	N2 *clk-1(qm30)* mutant	OP50 (live)	150	100	NAC	10 mM	1 day	No	*clk-1(qm30)* mutant
	97	N2 *daf-2(e1370)* mutant	OP50 (live)	150	100	NAC	10 mM	1 day	No	*daf-2(e1370)* mutant
	98	N2 *sod-2(ok1030)* mutant	OP50 (live)	100	100	NAC	10 mM	1 day	No	*sod-2(ok1030)* mutant

NAC: N-acetyl cysteine; SAC: S-allyl cysteine; SAMC: S-allylmercaptocysteine; ASSNAC: S-allylmercapto-N-acetylcysteine.

Table S6. Characteristics of 214 *Drosophila* lifespan experiments included in this review, from 5 published studies.

Study	Experiment index	Drosophila strains	Number of flies in the control group	Number of flies in the experiment group	Gender of flies	Type of cysteine	Cysteine dose	Other experimental characteristics
Brack, 1997	1	*D. melanogaster*	100	100	Male	NAC	1 mg/mL (6 mM)	
	2	*D. melanogaster*	100	100	Male	NAC	10 mg/mL (60 mM)	
	3	*D. melanogaster*	200	200	Male	NAC	0.1 mg/mL (0.6 mM)	
	4	*D. melanogaster*	200	200	Male	NAC	1 mg/mL (6 mM)	
	5	*D. melanogaster*	200	200	Male	NAC	10 mg/mL (60 mM)	
	6	*D. melanogaster*	200	200	Male	NAC	20 mg/ml (120 mM)	
Jouandin, 2022	7	*D. melanogaster*	90	90	Male	Cys-HCl	1 mM	
	8	*D. melanogaster*	90	90	Male	Cys-HCl	1 mM	dCTNS depletion
Niraula, 2019	9	*D. melanogaster*	140	140	Female	NAC	1 mg/mL (6 mM)	
	10	*D. melanogaster*	140	140	Female	NAC	10 mg/mL (60 mM)	
	11	*D. melanogaster*	140	140	Male	NAC	1 mg/mL (6 mM)	
	12	*D. melanogaster*	140	140	Male	NAC	10 mg/ml (60 mM)	
	13	*D. melanogaster*	138	138	Female	NAC	1 mg/mL (6 mM)	Starvation
	14	*D. melanogaster*	140	140	Female	NAC	1 mg/mL (6 mM)	Paraquat
Russi, 2020	15	*D. melanogaster*	143	282	Male	NAC	0.2 mg/mL (1 mM)	fh-200GAA; daGS>UAS-fh
	16	*D. melanogaster*	143	242	Male	NAC	0.4 mg/mL (2 mM)	fh-200GAA; daGS>UAS-fh
	17	*D. melanogaster*	143	149	Male	NAC	0.8 mg/mL (5 mM)	fh-200GAA; daGS>UAS-fh
	18	*D. melanogaster*	154	151	Male	NAC	0.8 mg/mL (5 mM)	fh-200GAA; daGS>UAS-fh
	19	*D. melanogaster*	89	116	Male	NAC	0.8 mg/mL (5 mM)	Hyperoxia; daGS>UAS-fh
	20	*D. melanogaster*	115	120	Male	NAC	0.8 mg/mL (5 mM)	Hyperoxia; fh-200GAA; daGS>UAS-fh
	21	*D. melanogaster*	118	118	Male	NAC	0.8 mg/mL (5 mM)	paraquat; daGS>UAS-fh

	22	*D. melanogaster*	107	120	Male	NAC	0.8 mg/mL (5 mM)	paraquat; fh-200GAA; daGS>UAS-fh
Shaposhnikov, 2018	23	*D. melanogaster*	535	306	Male	NAC	10 nM	
	24	*D. melanogaster*	535	296	Male	NAC	100 nM	
	25	*D. melanogaster*	535	297	Male	NAC	1 µM	
	26	*D. melanogaster*	535	299	Male	NAC	10 µM	
	27	*D. melanogaster*	535	299	Male	NAC	100 µM	
	28	*D. melanogaster*	535	304	Male	NAC	1 mM	
	29	*D. melanogaster*	535	271	Male	NAC	10 mM	
	30	*D. melanogaster*	535	269	Male	NAC	100 mM	
	31	*D. melanogaster*	583	312	Female	NAC	10 nM	
	32	*D. melanogaster*	583	306	Female	NAC	100 nM	
	33	*D. melanogaster*	583	276	Female	NAC	1 µM	
	34	*D. melanogaster*	583	307	Female	NAC	10 µM	
	35	*D. melanogaster*	583	295	Female	NAC	100 µM	
	36	*D. melanogaster*	583	295	Female	NAC	1 mM	
	37	*D. melanogaster*	583	289	Female	NAC	10 mM	
	38	*D. melanogaster*	583	277	Female	NAC	100 mM	
	39	*D. virilis*	588	287	Male	NAC	10 nM	
	40	*D. virilis*	588	285	Male	NAC	100 nM	
	41	*D. virilis*	588	287	Male	NAC	1 µM	
	42	*D. virilis*	588	288	Male	NAC	10 µM	
	43	*D. virilis*	588	282	Male	NAC	100 µM	
	44	*D. virilis*	588	293	Male	NAC	1 mM	
	45	*D. virilis*	588	328	Male	NAC	10 mM	
	46	*D. virilis*	588	276	Male	NAC	100 mM	
	47	*D. virilis*	640	314	Female	NAC	10 nM	
	48	*D. virilis*	640	293	Female	NAC	100 nM	
	49	*D. virilis*	640	330	Female	NAC	1 µM	
	50	*D. virilis*	640	299	Female	NAC	10 µM	
	51	*D. virilis*	640	320	Female	NAC	100 µM	
	52	*D. virilis*	640	287	Female	NAC	1 mM	
	53	*D. virilis*	640	445	Female	NAC	10 mM	
	54	*D. virilis*	640	438	Female	NAC	100 mM	
	55	*D. kikkawai*	538	298	Male	NAC	10 nM	
	56	*D. kikkawai*	538	292	Male	NAC	100 nM	
	57	*D. kikkawai*	538	285	Male	NAC	1 µM	
	58	*D. kikkawai*	538	299	Male	NAC	10 µM	
	59	*D. kikkawai*	538	312	Male	NAC	100 µM	
	60	*D. kikkawai*	538	292	Male	NAC	1 mM	
	61	*D. kikkawai*	538	293	Male	NAC	10 mM	
	62	*D. kikkawai*	538	296	Male	NAC	100 mM	
	63	*D. kikkawai*	641	293	Female	NAC	10 nM	
	64	*D. kikkawai*	641	295	Female	NAC	100 nM	
	65	*D. kikkawai*	641	297	Female	NAC	1 µM	
	66	*D. kikkawai*	641	299	Female	NAC	10 µM	
	67	*D. kikkawai*	641	290	Female	NAC	100 µM	
	68	*D. kikkawai*	641	311	Female	NAC	1 mM	
	69	*D. kikkawai*	641	296	Female	NAC	10 mM	
	70	*D. kikkawai*	641	288	Female	NAC	100 mM	
	71	*D. melanogaster*	150	150	Male	NAC	10 nM	Paraquat
	72	*D. melanogaster*	150	150	Male	NAC	100 nM	Paraquat

	73	*D. melanogaster*	150	150	Male	NAC	1 µM	Paraquat
	74	*D. melanogaster*	150	150	Male	NAC	10 µM	Paraquat
	75	*D. melanogaster*	150	150	Male	NAC	100 µM	Paraquat
	76	*D. melanogaster*	150	150	Male	NAC	1 mM	Paraquat
	77	*D. melanogaster*	150	150	Male	NAC	10 mM	Paraquat
	78	*D. melanogaster*	150	150	Male	NAC	100 mM	Paraquat
	79	*D. melanogaster*	150	150	Female	NAC	10 nM	Paraquat
	80	*D. melanogaster*	150	150	Female	NAC	100 nM	Paraquat
	81	*D. melanogaster*	150	150	Female	NAC	1 µM	Paraquat
	82	*D. melanogaster*	150	150	Female	NAC	10 µM	Paraquat
	83	*D. melanogaster*	150	150	Female	NAC	100 µM	Paraquat
	84	*D. melanogaster*	150	150	Female	NAC	1 mM	Paraquat
	85	*D. melanogaster*	150	150	Female	NAC	10 mM	Paraquat
	86	*D. melanogaster*	150	150	Female	NAC	100 mM	Paraquat
	87	*D. melanogaster*	150	150	Male	NAC	10 nM	Starvation
	88	*D. melanogaster*	150	150	Male	NAC	100 nM	Starvation
	89	*D. melanogaster*	150	150	Male	NAC	1 µM	Starvation
	90	*D. melanogaster*	150	150	Male	NAC	10 µM	Starvation
	91	*D. melanogaster*	150	150	Male	NAC	100 µM	Starvation
	92	*D. melanogaster*	150	150	Male	NAC	1 mM	Starvation
	93	*D. melanogaster*	150	150	Male	NAC	10 mM	Starvation
	94	*D. melanogaster*	150	150	Male	NAC	100 mM	Starvation
	95	*D. melanogaster*	150	150	Female	NAC	10 nM	Starvation
	96	*D. melanogaster*	150	150	Female	NAC	100 nM	Starvation
	97	*D. melanogaster*	150	150	Female	NAC	1 µM	Starvation
	98	*D. melanogaster*	150	150	Female	NAC	10 µM	Starvation
	99	*D. melanogaster*	150	150	Female	NAC	100 µM	Starvation
	100	*D. melanogaster*	150	150	Female	NAC	1 mM	Starvation
	101	*D. melanogaster*	150	150	Female	NAC	10 mM	Starvation
	102	*D. melanogaster*	150	150	Female	NAC	100 mM	Starvation
	103	*D. melanogaster*	150	150	Male	NAC	10 nM	Hyperthermia
	104	*D. melanogaster*	150	150	Male	NAC	100 nM	Hyperthermia
	105	*D. melanogaster*	150	150	Male	NAC	1 µM	Hyperthermia
	106	*D. melanogaster*	150	150	Male	NAC	10 µM	Hyperthermia
	107	*D. melanogaster*	150	150	Male	NAC	100 µM	Hyperthermia
	108	*D. melanogaster*	150	150	Male	NAC	1 mM	Hyperthermia
	109	*D. melanogaster*	150	150	Male	NAC	10 mM	Hyperthermia
	110	*D. melanogaster*	150	150	Male	NAC	100 mM	Hyperthermia
	111	*D. melanogaster*	150	150	Female	NAC	10 nM	Hyperthermia
	112	*D. melanogaster*	150	150	Female	NAC	100 nM	Hyperthermia
	113	*D. melanogaster*	150	150	Female	NAC	1 µM	Hyperthermia
	114	*D. melanogaster*	150	150	Female	NAC	10 µM	Hyperthermia
	115	*D. melanogaster*	150	150	Female	NAC	100 µM	Hyperthermia
	116	*D. melanogaster*	150	150	Female	NAC	1 mM	Hyperthermia
	117	*D. melanogaster*	150	150	Female	NAC	10 mM	Hyperthermia
	118	*D. melanogaster*	150	150	Female	NAC	100 mM	Hyperthermia
	119	*D. virilis*	150	150	Male	NAC	10 nM	Paraquat
	120	*D. virilis*	150	150	Male	NAC	100 nM	Paraquat
	121	*D. virilis*	150	150	Male	NAC	1 µM	Paraquat
	122	*D. virilis*	150	150	Male	NAC	10 µM	Paraquat
	123	*D. virilis*	150	150	Male	NAC	100 µM	Paraquat
	124	*D. virilis*	150	150	Male	NAC	1 mM	Paraquat
	125	*D. virilis*	150	150	Male	NAC	10 mM	Paraquat

	126	*D. virilis*	150	150	Male	NAC	100 mM	Paraquat
	127	*D. virilis*	150	150	Female	NAC	10 nM	Paraquat
	128	*D. virilis*	150	150	Female	NAC	100 nM	Paraquat
	129	*D. virilis*	150	150	Female	NAC	1 µM	Paraquat
	130	*D. virilis*	150	150	Female	NAC	10 µM	Paraquat
	131	*D. virilis*	150	150	Female	NAC	100 µM	Paraquat
	132	*D. virilis*	150	150	Female	NAC	1 mM	Paraquat
	133	*D. virilis*	150	150	Female	NAC	10 mM	Paraquat
	134	*D. virilis*	150	150	Female	NAC	100 mM	Paraquat
	135	*D. virilis*	150	150	Male	NAC	10 nM	Starvation
	136	*D. virilis*	150	150	Male	NAC	100 nM	Starvation
	137	*D. virilis*	150	150	Male	NAC	1 µM	Starvation
	138	*D. virilis*	150	150	Male	NAC	10 µM	Starvation
	139	*D. virilis*	150	150	Male	NAC	100 µM	Starvation
	140	*D. virilis*	150	150	Male	NAC	1 mM	Starvation
	141	*D. virilis*	150	150	Male	NAC	10 mM	Starvation
	142	*D. virilis*	150	150	Male	NAC	100 mM	Starvation
	143	*D. virilis*	150	150	Female	NAC	10 nM	Starvation
	144	*D. virilis*	150	150	Female	NAC	100 nM	Starvation
	145	*D. virilis*	150	150	Female	NAC	1 µM	Starvation
	146	*D. virilis*	150	150	Female	NAC	10 µM	Starvation
	147	*D. virilis*	150	150	Female	NAC	100 µM	Starvation
	148	*D. virilis*	150	150	Female	NAC	1 mM	Starvation
	149	*D. virilis*	150	150	Female	NAC	10 mM	Starvation
	150	*D. virilis*	150	150	Female	NAC	100 mM	Starvation
	151	*D. virilis*	150	150	Male	NAC	10 nM	Hyperthermia
	152	*D. virilis*	150	150	Male	NAC	100 nM	Hyperthermia
	153	*D. virilis*	150	150	Male	NAC	1 µM	Hyperthermia
	154	*D. virilis*	150	150	Male	NAC	10 µM	Hyperthermia
	155	*D. virilis*	150	150	Male	NAC	100 µM	Hyperthermia
	156	*D. virilis*	150	150	Male	NAC	1 mM	Hyperthermia
	157	*D. virilis*	150	150	Male	NAC	10 mM	Hyperthermia
	158	*D. virilis*	150	150	Male	NAC	100 mM	Hyperthermia
	159	*D. virilis*	150	150	Female	NAC	10 nM	Hyperthermia
	160	*D. virilis*	150	150	Female	NAC	100 nM	Hyperthermia
	161	*D. virilis*	150	150	Female	NAC	1 µM	Hyperthermia
	162	*D. virilis*	150	150	Female	NAC	10 µM	Hyperthermia
	163	*D. virilis*	150	150	Female	NAC	100 µM	Hyperthermia
	164	*D. virilis*	150	150	Female	NAC	1 mM	Hyperthermia
	165	*D. virilis*	150	150	Female	NAC	10 mM	Hyperthermia
	166	*D. virilis*	150	150	Female	NAC	100 mM	Hyperthermia
	167	*D. kikkawai*	150	150	Male	NAC	10 nM	Paraquat
	168	*D. kikkawai*	150	150	Male	NAC	100 nM	Paraquat
	169	*D. kikkawai*	150	150	Male	NAC	1 µM	Paraquat
	170	*D. kikkawai*	150	150	Male	NAC	10 µM	Paraquat
	171	*D. kikkawai*	150	150	Male	NAC	100 µM	Paraquat
	172	*D. kikkawai*	150	150	Male	NAC	1 mM	Paraquat
	173	*D. kikkawai*	150	150	Male	NAC	10 mM	Paraquat
	174	*D. kikkawai*	150	150	Male	NAC	100 mM	Paraquat
	175	*D. kikkawai*	150	150	Female	NAC	10 nM	Paraquat
	176	*D. kikkawai*	150	150	Female	NAC	100 nM	Paraquat
	177	*D. kikkawai*	150	150	Female	NAC	1 µM	Paraquat
	178	*D. kikkawai*	150	150	Female	NAC	10 µM	Paraquat

	179	*D. kikkawai*	150	150	Female	NAC	100 µM	Paraquat
	180	*D. kikkawai*	150	150	Female	NAC	1 mM	Paraquat
	181	*D. kikkawai*	150	150	Female	NAC	10 mM	Paraquat
	182	*D. kikkawai*	150	150	Female	NAC	100 mM	Paraquat
	183	*D. kikkawai*	150	150	Male	NAC	10 nM	Starvation
	184	*D. kikkawai*	150	150	Male	NAC	100 nM	Starvation
	185	*D. kikkawai*	150	150	Male	NAC	1 µM	Starvation
	186	*D. kikkawai*	150	150	Male	NAC	10 µM	Starvation
	187	*D. kikkawai*	150	150	Male	NAC	100 µM	Starvation
	188	*D. kikkawai*	150	150	Male	NAC	1 mM	Starvation
	189	*D. kikkawai*	150	150	Male	NAC	10 mM	Starvation
	190	*D. kikkawai*	150	150	Male	NAC	100 mM	Starvation
	191	*D. kikkawai*	150	150	Female	NAC	10 nM	Starvation
	192	*D. kikkawai*	150	150	Female	NAC	100 nM	Starvation
	193	*D. kikkawai*	150	150	Female	NAC	1 µM	Starvation
	194	*D. kikkawai*	150	150	Female	NAC	10 µM	Starvation
	195	*D. kikkawai*	150	150	Female	NAC	100 µM	Starvation
	196	*D. kikkawai*	150	150	Female	NAC	1 mM	Starvation
	197	*D. kikkawai*	150	150	Female	NAC	10 mM	Starvation
	198	*D. kikkawai*	150	150	Female	NAC	100 mM	Starvation
	199	*D. kikkawai*	150	150	Male	NAC	10 nM	Hyperthermia
	200	*D. kikkawai*	150	150	Male	NAC	100 nM	Hyperthermia
	201	*D. kikkawai*	150	150	Male	NAC	1 µM	Hyperthermia
	202	*D. kikkawai*	150	150	Male	NAC	10 µM	Hyperthermia
	203	*D. kikkawai*	150	150	Male	NAC	100 µM	Hyperthermia
	204	*D. kikkawai*	150	150	Male	NAC	1 mM	Hyperthermia
	205	*D. kikkawai*	150	150	Male	NAC	10 mM	Hyperthermia
	206	*D. kikkawai*	150	150	Male	NAC	100 mM	Hyperthermia
	207	*D. kikkawai*	150	150	Female	NAC	10 nM	Hyperthermia
	208	*D. kikkawai*	150	150	Female	NAC	100 nM	Hyperthermia
	209	*D. kikkawai*	150	150	Female	NAC	1 µM	Hyperthermia
	210	*D. kikkawai*	150	150	Female	NAC	10 µM	Hyperthermia
	211	*D. kikkawai*	150	150	Female	NAC	100 µM	Hyperthermia
	212	*D. kikkawai*	150	150	Female	NAC	1 mM	Hyperthermia
	213	*D. kikkawai*	150	150	Female	NAC	10 mM	Hyperthermia
	214	*D. kikkawai*	150	150	Female	NAC	100 mM	Hyperthermia

NAC: N-acetyl cysteine. Cys-HCl: Cysteine hydrochloride.

Table S7a. Thirteen mouse lifespan studies included in the funnel plot.

Study Index	First author, year	Study name	PubMed ID
1	Andreassen, 2020	N-acetyl-L-cysteine improves survival and preserves motor performance in an animal model of familial amyotrophic lateral sclerosis	10943709
2	Chen, 2019	N-acetyl-L-cysteine supplement in early life or adulthood reduces progression of diabetes in nonobese diabetic mice	30907059
3	Chen, 2020	TGF-β1/IL-11/MEK/ERK signaling mediates senescence-associated pulmonary fibrosis in a stress-induced premature senescence model of Bmi-1 deficiency	31959867
4	Flurkey, 2010	Life extension by diet restriction and N-acetyl-L-cysteine in genetically heterogeneous mice	20819793
5	Frenkel, 2019	N-acetyl-L-cysteine supplement in early life or adulthood reduces progression of diabetes in nonobese diabetic mice	30993256
6	Harman, 1957	Prolongation of the normal life span by radiation protection chemicals	13463294
7	Harman, 1961	Prolongation of the normal lifespan and inhibition of spontaneous cancer by antioxidants	13711616
8	Jin, 2014	Bmi-1 plays a critical role in protection from renal tubulointerstitial injury by maintaining redox balance	24915841
9	Kondratov, 2009	Antioxidant N-acetyl-L-cysteine ameliorates symptoms of premature aging associated with the deficiency of the circadian protein BMAL1	20157581
10	Kumar, 2022	GlyNAC (Glycine and N-Acetylcysteine) supplementation in mice increases length of life by correcting glutathione deficiency, oxidative stress, mitochondrial dysfunction, abnormalities in mitophagy and nutrient sensing, and genomic damage	35268089
11	kurano, 2022	Intranasal Administration of N-acetyl-L-cysteine Combined with Cell-Penetrating Peptide-Modified Polymer Nanomicelles as a Potential Therapeutic Approach for Amyotrophic Lateral Sclerosis	36559085
12	Paul, 2014	Cystathionine γ-lyase deficiency mediates neurodegeneration in Huntington's disease	24670645
13	Reliene, 2006	Antioxidant N-acetyl cysteine reduces incidence and multiplicity of lymphoma in Atm deficient mice	16781197

Table S7b. Thirteen *C. elegans* lifespan studies included in the funnel plot.

Study Index	First author, year	Study name	PubMed ID
1	Desjardins, 2017	Antioxidants reveal an inverted U-shaped dose-response relationship between reactive oxygen species levels and the rate of aging in *Caenorhabditis elegans*	27683245
2	Gusarov, 2021	Dietary thiols accelerate aging of *C. elegans*	34267196
3	Kim, 2016	Selenocysteine modulates resistance to environmental stress and confers anti-aging effects in *C. elegans*	28954009
4	Kim, 2017	Supplementation of S-allyl cysteine improves health span in *Caenorhabditis elegans*	*
5	Kim, 2018	Selenocysteine mimics the effect of dietary restriction on lifespan via SKN-1 and retards age-associated pathophysiological changes in *Caenorhabditis elegans*	30365103
6	Ogawa, 2016	Natural thioallyl compounds increase oxidative stress resistance and lifespan in Caenorhabditis elegans by modulating SKN-1/Nrf	26899496
7	Oh, 2015	Lifespan extension and increased resistance to environmental stressors by N-acetyl-L-cysteine in *Caenorhabditis elegans*	26039957
8	Oh, 2017	N-acetyl-L-cysteine mimics the effect of dietary restriction on lifespan and reduces amyloid beta-induced toxicity in *Caenorhabditis elegans*	30263604
9	Polyak, 2018	N-acetylcysteine and vitamin E rescue animal longevity and cellular oxidative stress in pre-clinical models of mitochondrial complex I disease	29526616
10	Savion, 2018	S-allylmercapto-N-acetylcysteine protects against oxidative stress and extends lifespan in *Caenorhabditis elegans*	29579097
11	Shibamura, 2009	A method for oral administration of hydrophilic substances to *Caenorhabditis elegans*: Effects of oral supplementation with antioxidants on the nematode lifespan	19580823
12	Wei, 2016	Roles for ROS and hydrogen sulfide in the longevity response to germline loss in Caenorhabditis elegans	27140632
13	Yang, 2010	A Mitochondrial Superoxide Signal Triggers Increased Longevity in Caenorhabditis elegans	21151885

* PubMed ID not available. doi: 10.14393/BJ-v33n2-32846

Table S7c. Five *Drosophila* lifespan studies included in the funnel plot.

Study Index	First author, year	Study name	PubMed ID
1	Brack, 1997	N-Acetylcysteine slows down ageing and increases the life span of *Drosophila melanogaster*	9447249
2	Jouandin, 2022	Lysosomal cystine mobilization shapes the response of TORC1 and tissue growth to fasting	35175796
3	Niraula, 2019	N-Acetylcysteine extends lifespan of *Drosophila* via modulating ROS scavenger gene expression	31115735
4	Russi, 2020	A *Drosophila* model of Friedreich ataxia with CRISPR/Cas9 insertion of GAA repeats in the frataxin gene reveals in vivo protection by N-acetyl cysteine	32744307
5	Shaposhnikov, 2018	Effects of N-acetyl-L-cysteine on lifespan, locomotor activity and stress-resistance of 3 *Drosophila* species with different lifespans	30243020

Supplementary Figures

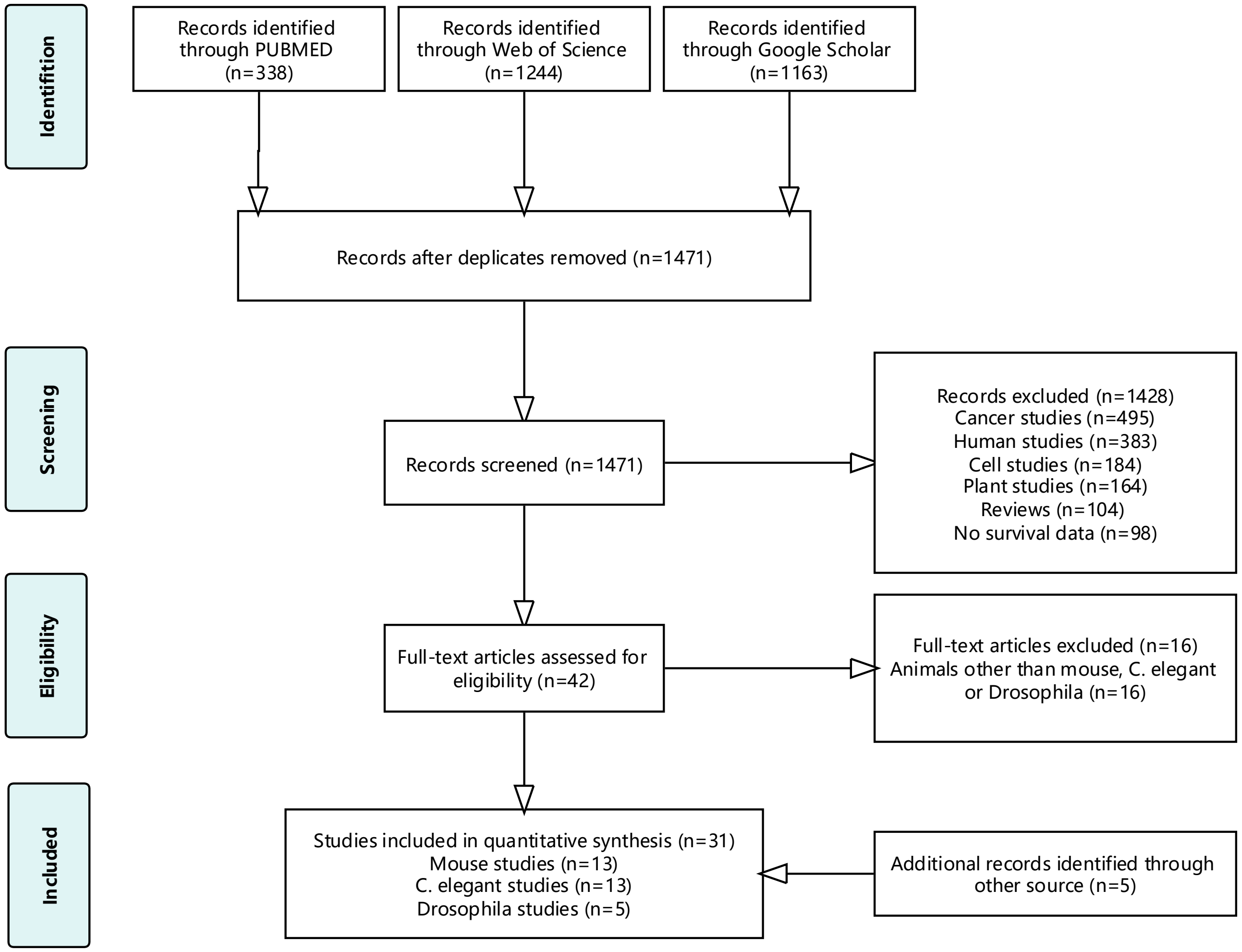

Figure S1. PRISMA flow diagram for the review.

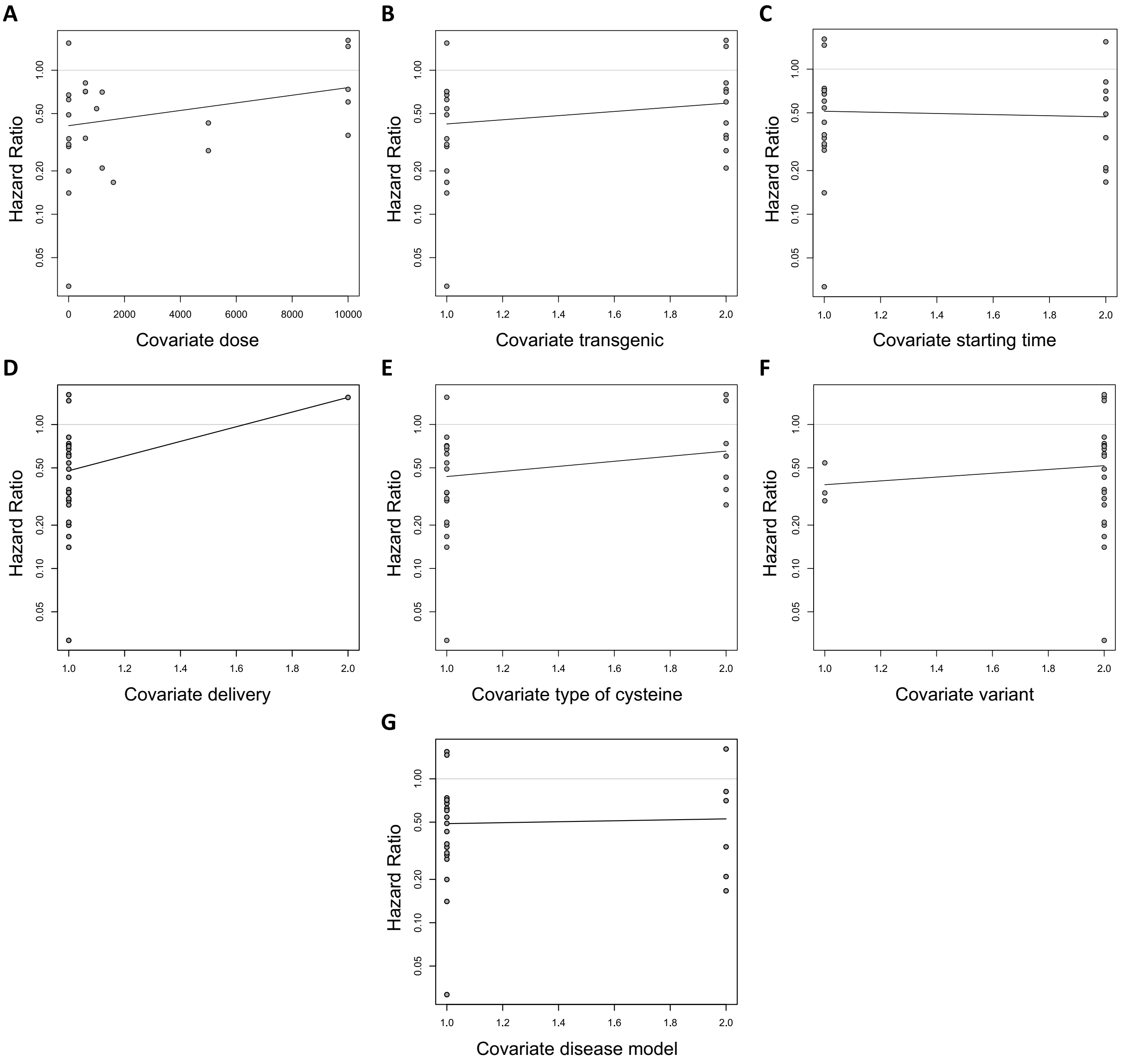

Figure S2. Random-effects meta-regression plots of the association between experimental conditions and *HR* for all mice experiments.

(A) dose; (B) transgenic; (C) starting time; (D) delivery; € type of cysteine; (F) variants; (G) disease model.

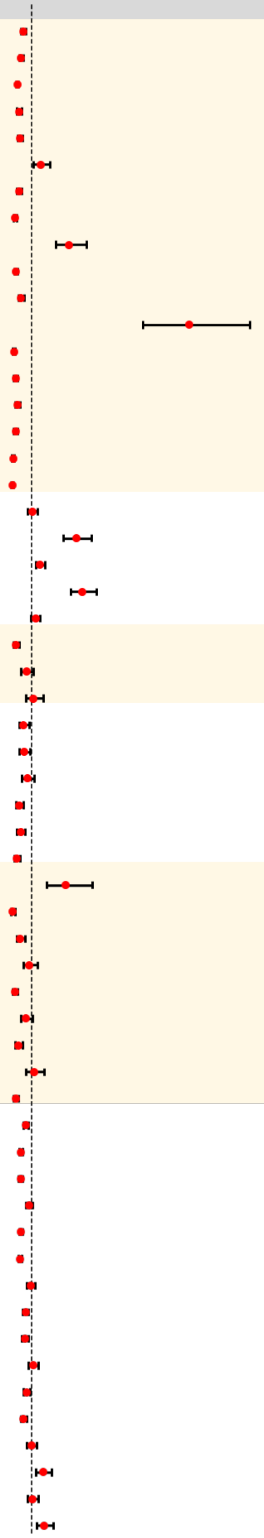

Study	Index		Hazard ratio (95% CI)	
Desjardins, 2017	1	N2 WT, 3mM	0.62 (0.52-0.76)	***
		N2 WT, 6mM	0.52 (0.43-0.63)	***
		N2 WT, 9mM	0.35 (0.28-0.43)	***
		N2 bus-8 mutant, 3mM	0.43 (0.33-0.56)	***
		N2 bus-8 mutant, 6mM	0.47 (0.36-0.61)	***
		N2 bus-8 mutant, 9mM	1.39 (1.07-1.82)	*
		N2 clk-1 mutant, 3mM	0.44 (0.34-0.57)	***
		N2 clk-1 mutant, 6mM	0.25 (0.19-0.34)	***
		N2 clk-1 mutant, 9mM	2.69 (2.09-3.47)	***
		N2 bus-8; clk-1 mutant, 3mM	0.28 (0.21-0.36)	***
		N2 bus-8; clk-1 mutant, 6mM	0.52 (0.41-0.66)	***
		N2 bus-8; clk-1 mutant, 9mM	8.09 (6.04-10.85)	***
		N2 isp-1 mutant, 3mM	0.20 (0.15-0.27)	***
		N2 isp-1 mutant, 6mM	0.27 (0.21-0.36)	***
		N2 isp-1 mutant, 9mM	0.36 (0.27-0.47)	***
		N2 sod-12345 mutant, 3mM	0.27 (0.20-0.37)	***
		N2 sod-12345 mutant, 6mM	0.17 (0.12-0.23)	***
		N2 sod-12345 mutant, 9mM	0.14 (0.09-0.20)	***
Gusarov, 2021	2	N2 Bristol WT, OP50 live, 1d, 5mM	1.03 (0.83-1.28)	***
		N2 Bristol WT, OP50 live, 1d, 15mM	3.02 (2.44-3.72)	***
		N2 Bristol WT, OP50 dead, 1d, 5mM	1.37 (1.18-1.60)	***
		N2 Bristol WT, OP50 dead, 1d, 15mM	3.29 (2.76-3.91)	***
		N2 Bristol WT, OP50 dead, 13d, 5mM	1.17 (0.99-1.39)	
Kim, 2016	3	N2 CGCb WT, H2O2	0.29 (0.19-0.45)	***
		N2 CGCb WT, Heat	0.75 (0.52-1.08)	
		N2 CGCb WT, UV	1.06 (0.74-1.52)	
Kim, 2017	4	N2 CGCb WT, 1mM, UV	0.63 (0.44-0.91)	**
		N2 CGCb WT, 2.5mM, UV	0.64 (0.45-0.92)	*
		N2 CGCb WT, 5mM, UV	0.79 (0.55-1.14)	
		N2 CGCb WT, 1mM	0.44 (0.30-0.64)	***
		N2 CGCb WT, 2.5mM	0.49 (0.34-0.71)	***
		N2 CGCb WT, 5mM	0.32 (0.21-0.47)	***
Kim, 2018	5	N2 CGCb WT	2.52 (1.70-3.75)	***
		N2 age-1 mutant	0.15 (0.09-0.24)	***
		N2 clk-1 mutant	0.48 (0.33-0.70)	***
		N2 eat-2 mutant	0.90 (0.62-1.29)	
		N2 CGCbWT	0.24 (0.15-0.37)	***
		N2 CGCbWT, DR	0.74 (0.51-1.06)	
		N2 CGCbWT, EV	0.38 (0.25-0.58)	***
		N2 skn-1 mutant	1.10 (0.77-1.59)	
		N2 CGCbWT, HG	0.28 (0.19-0.43)	***
Ogawa, 2016	6	N2 WT, SAC, 1µl	0.75 (0.64-0.88)	***
		N2 WT, SAC, 10µl	0.52 (0.44-0.61)	***
		N2 WT, SAC, 100µl	0.49 (0.42-0.58)	***
		N2 WT, SAMC, 1µl	0.88 (0.75-1.03)	
		N2 WT, SAMC, 10µl	0.51 (0.43-0.60)	***
		N2 WT, SAMC, 100µl	0.48 (0.41-0.57)	***
		N2 daf-16 mutant, SAC, 1µl	0.95 (0.78-1.17)	
		N2 daf-16 mutant, SAC, 10µl	0.72 (0.58-0.88)	***
		N2 daf-16 mutant, SAC, 100µl	0.70 (0.57-0.86)	***
		N2 daf-16 mutant, SAMC, 1µl	1.07 (0.87-1.30)	
		N2 daf-16 mutant, SAMC, 10µl	0.76 (0.62-0.92)	**
		N2 daf-16 mutant, SAMC, 100µl	0.63 (0.51-0.77)	***
		N2 skn-1 mutant, SAC, 10µl	0.98 (0.77-1.24)	
		N2 skn-1 mutant, SAC, 100µl	1.50 (1.18-1.91)	***
		N2 skn-1 mutant, SAMC, 10µl	1.03 (0.81-1.31)	
		N2 skn-1 mutant, SAMC, 100µl	1.56 (1.23-1.98)	***

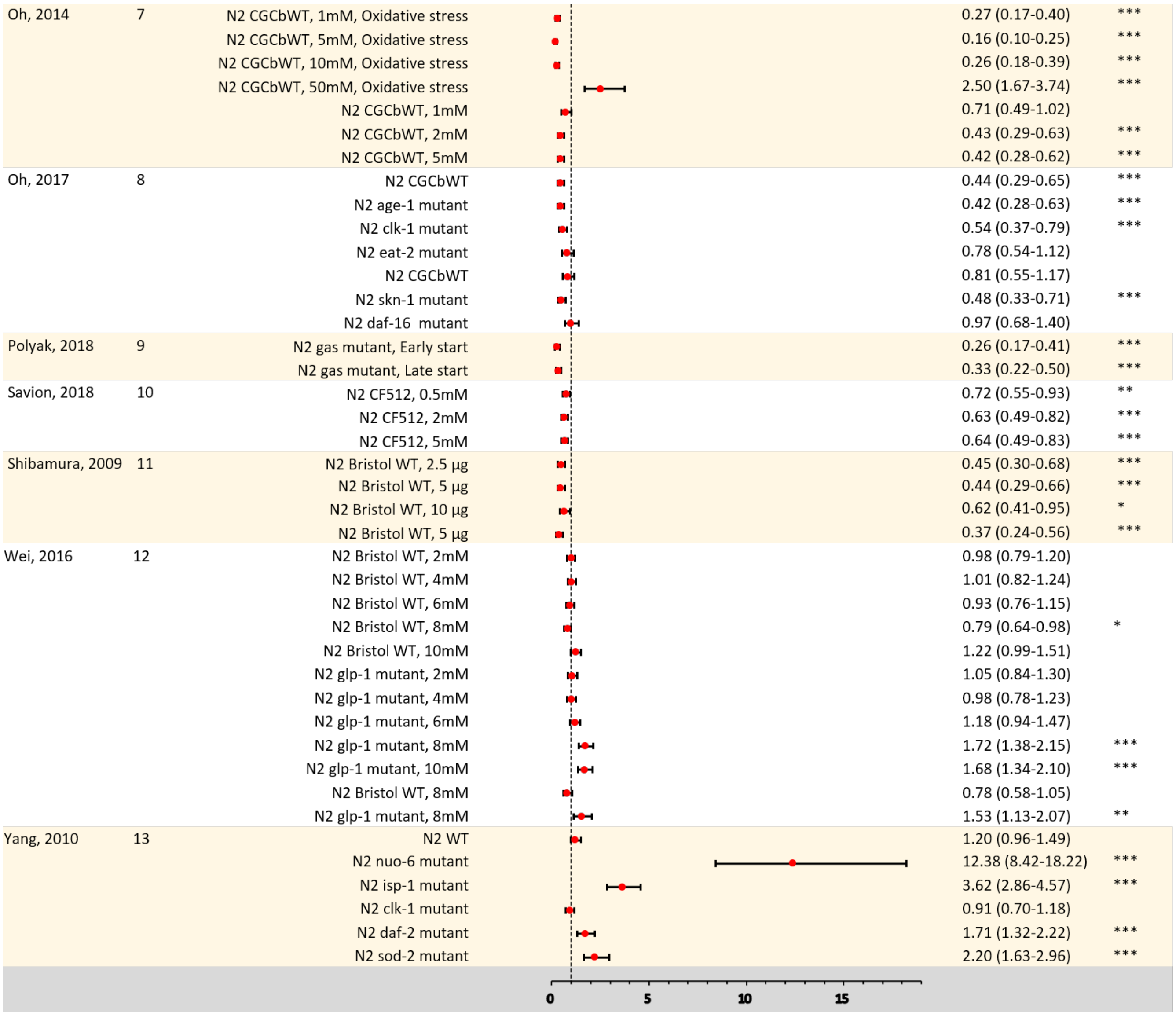

Figure S3. Forest plot of hazard ratios for all *C. elegans* experiments.

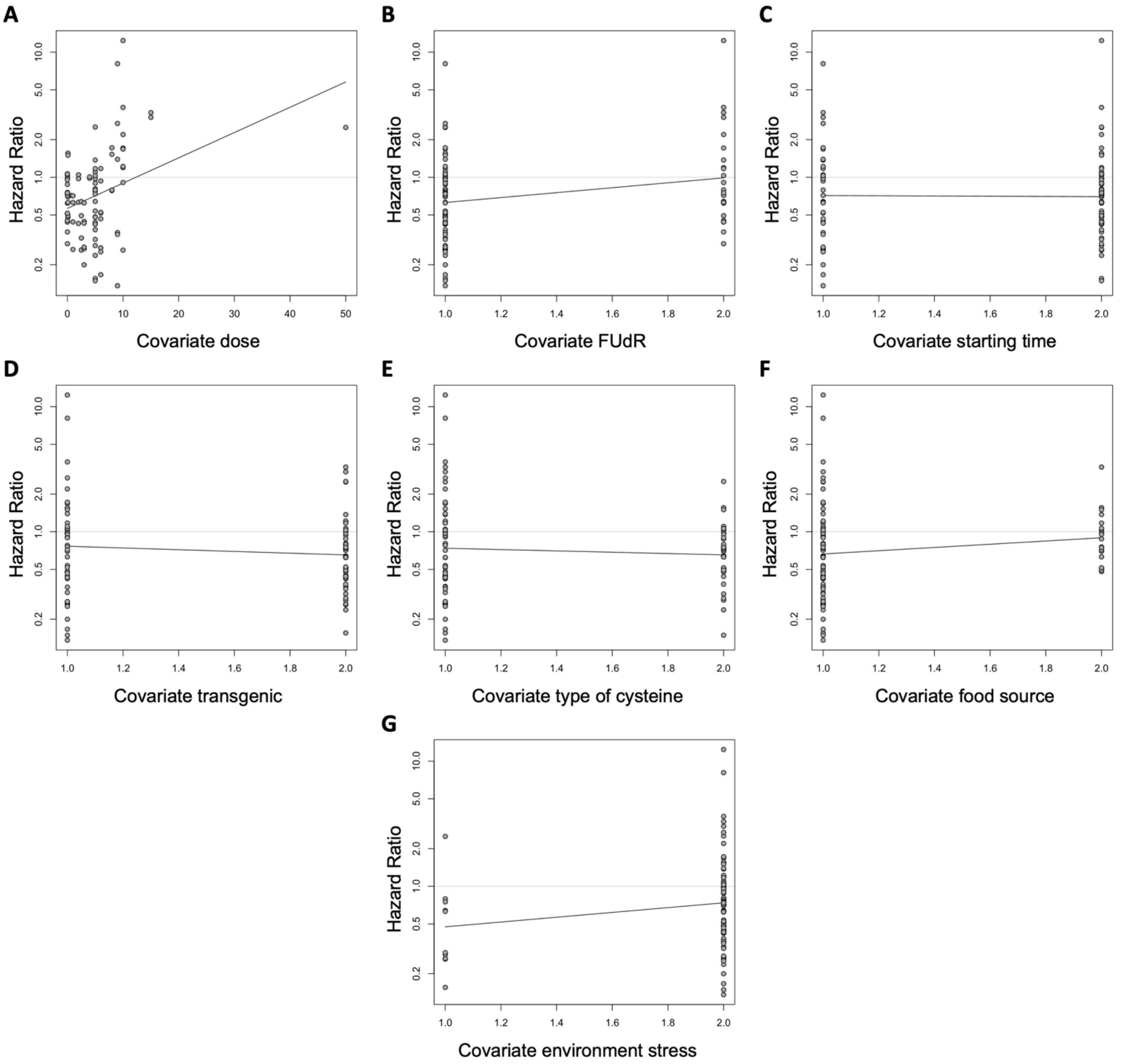

Figure S4. Random-effects meta-regression plots of the association between experimental conditions and *HR* for all *C. elegans* experiment

A) dose; (B) FUdR; (C) starting age; (D) transgenic; (E) type of cysteine; (F) food source; (G) environment stress.

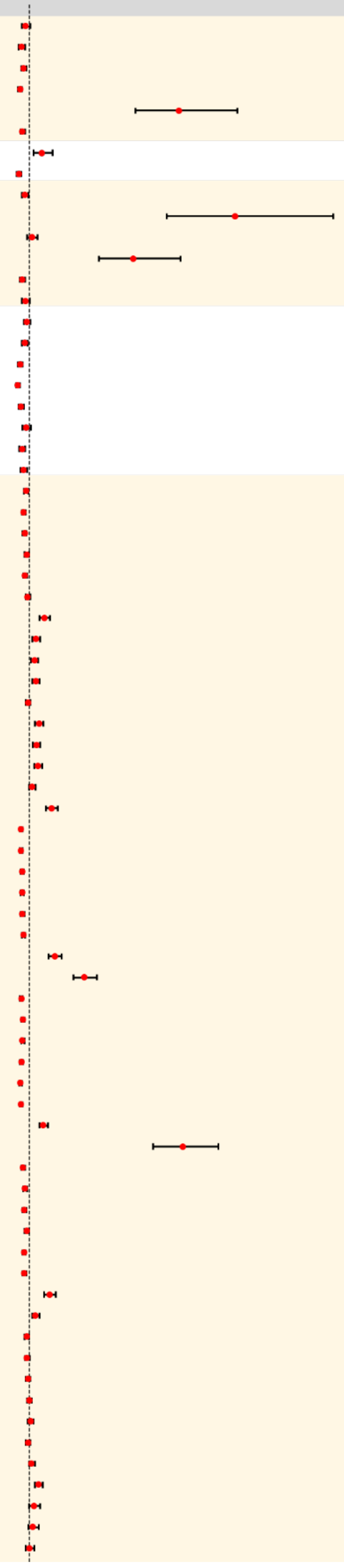

Study	Index		Hazard ratio (95% CI)	
Brack, 1997	1	D. melanogaster, 1 mg/ml	0.79 (0.0.60–1.05)	
		D. melanogaster, 10 mg/ml	0.58 (0.0.44–0.77)	***
		D. melanogaster, 0.1 mg/ml	0.68 (0.56-0.83)	***
		D. melanogaster, 1 mg/ml	0.50 (0.40-0.61)	***
		D. melanogaster, 10 mg/ml	9.03 (9.6.69–12.17)	***
		D. melanogaster, 20 mg/ml	0.63 (0.52-0.77)	***
Jouandin, 2022	2	D. melanogaster	1.67 (1.25-2.25)	***
		D. melanogaster, dCTNS depletion	0.43 (0.0.31–0.58)	***
Niraula, 2019	3	D. melanogaster, F, 1 mg/ml	0.76 (0.60-0.97)	*
		D. melanogaster, F, 10 mg/ml	12.07 (8.41-17.34)	***
		D. melanogaster, M, 1 mg/ml	1.13 (1.0.89–1.43)	
		D. melanogaster, M, 10 mg/ml	6.57 (4.73-9.13)	***
		D. melanogaster, F, 1 mg/ml, Starvation	0.61 (0.48-0.78)	***
		D. melanogaster, F, 1 mg/ml, Paraquat	0.78 (0.0.60–1.00)	
Russi, 2020	4	D. melanogaster, 0.2 mg/ml	0.86 (0.71-1.06)	
		D. melanogaster, 0.4 mg/ml	0.76 (0.61-0.93)	**
		D. melanogaster, 0.8 mg/ml	0.50 (0.39-0.63)	***
		D. melanogaster, 0.8 mg/ml	0.38 (0.30-0.49)	***
		D. melanogaster, 0.8 mg/ml, Hyperoxia	0.54 (0.41-0.70)	***
		D. melanogaster, 0.8 mg/ml, Hyperoxia	0.81 (0.62-1.07)	
		D. melanogaster, 0.8 mg/ml, Paraquat	0.60 (0.46-0.79)	***
		D. melanogaster, 0.8 mg/ml, Paraquat	0.68 (0.52-0.89)	**
Shaposhnikov, 2018	5	D. melanogaster, M, 10 nM	0.82 (0.71–0.94)	**
		D. melanogaster, M, 100 nM	0.69 (0.0.60–0.80)	***
		D. melanogaster, M, 1 µM	0.74 (0.0.64–0.85)	***
		D. melanogaster, M, 10 µM	0.85 (0.0.74–0.98)	*
		D. melanogaster, M, 100 µM	0.76 (0.0.66–0.88)	***
		D. melanogaster, M, 1 mM	0.91 (0.0.79–1.05)	
		D. melanogaster, M, 10 mM	1.80 (1.1.55–2.09)	***
		D. melanogaster, M, 100 mM	1.35 (1.1.17–1.57)	***
		D. melanogaster, F, 10 nM	1.27 (1.1.11–1.46)	***
		D. melanogaster, F, 100 nM	1.35 (1.1.17–1.55)	***
		D. melanogaster, F, 1 µM	0.92 (0.0.80–1.07)	
		D. melanogaster, F, 10 µM	1.51 (1.1.32–1.74)	***
		D. melanogaster, F, 100 µM	1.38 (1.1.20–1.59)	***
		D. melanogaster, F, 1 mM	1.46 (1.1.27–1.68)	***
		D. melanogaster, F, 10 mM	1.15 (1.1.00–1.32)	
		D. melanogaster, F, 100 mM	2.20 (2.1.89–2.55)	***
		D. virilis, M, 10 nM	0.56 (0.0.48–0.64)	***
		D. virilis, M, 100 nM	0.55 (0.0.48–0.64)	***
		D. virilis, M, 1 µM	0.62 (0.0.54–0.72)	***
		D. virilis, M, 10 µM	0.60 (0.0.52–0.70)	***
		D. virilis, M, 100 µM	0.63 (0.0.55–0.73)	***
		D. virilis, M, 1 mM	0.67 (0.0.58–0.77)	***
		D. virilis, M, 10 mM	2.37 (2.2.05–2.75)	***
		D. virilis, M, 100 mM	3.95 (3.3.36–4.65)	***
		D. virilis, F, 10 nM	0.57 (0.0.49–0.65)	***
		D. virilis, F, 100 nM	0.65 (0.0.56–0.74)	***
		D. virilis, F, 1 µM	0.63 (0.0.55–0.72)	***
		D. virilis, F, 10 µM	0.58 (0.0.51–0.67)	***
		D. virilis, F, 100 µM	0.53 (0.0.46–0.61)	***
		D. virilis, F, 1 mM	0.55 (0.0.48–0.64)	***
		D. virilis, F, 10 mM	1.75 (1.1.55–1.99)	***
		D. virilis, F, 100 mM	9.25 (9.7.66–11.17)	***
		D. kikkawai, M, 10 nM	0.66 (0.0.57–0.77)	***
		D. kikkawai, M, 100 nM	0.76 (0.0.66–0.88)	***
		D. kikkawai, M, 1 µM	0.74 (0.0.64–0.85)	***
		D. kikkawai, M, 10 µM	0.85 (0.0.74–0.99)	*
		D. kikkawai, M, 100 µM	0.72 (0.0.62–0.83)	***
		D. kikkawai, M, 1 mM	0.72 (0.0.63–0.83)	***
		D. kikkawai, M, 10 mM	2.08 (2.1.80–2.42)	***
		D. kikkawai, M, 100 mM	1.34 (1.1.16–1.54)	***
		D. kikkawai, F, 10 nM	0.86 (0.0.74–0.98)	*
		D. kikkawai, F, 100 nM	0.88 (0.0.77–1.01)	
		D. kikkawai, F, 1 µM	0.93 (0.0.81–1.07)	
		D. kikkawai, F, 10 µM	0.99 (0.0.86–1.14)	
		D. kikkawai, F, 100 µM	1.07 (1.0.93–1.23)	
		D. kikkawai, F, 1 mM	0.93 (0.0.81–1.07)	
		D. kikkawai, F, 10 mM	1.12 (1.0.98–1.29)	
		D. kikkawai, F, 100 mM	1.49 (1.1.29–1.71)	***
		D. melanogaster, M, 10 nM, Paraquat	1.25 (1.1.00–1.57)	
		D. melanogaster, M, 100 nM, Paraquat	1.19 (1.0.95–1.50)	
		D. melanogaster, M, 1 µM, Paraquat	1.00 (1.0.80–1.25)	

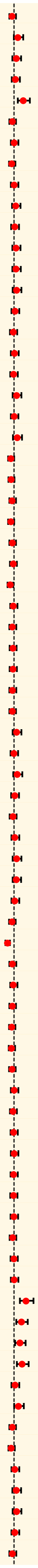

D. melanogaster, M, 10 µM, Paraquat	0.88 (0.0.70–1.11)	
D. melanogaster, M, 100 µM, Paraquat	1.23 (1.0.98–1.55)	
D. melanogaster, M, 1 mM, Paraquat	1.13 (1.0.90–1.42)	
D. melanogaster, M, 10 mM, Paraquat	1.08 (1.0.86–1.35)	
D. melanogaster, M, 100 mM, Paraquat	1.55 (1.1.23–1.95)	***
D. melanogaster, F, 10 nM, Paraquat	0.89 (0.0.71–1.11)	
D. melanogaster, F, 100 nM, Paraquat	1.01 (1.0.80–1.26)	
D. melanogaster, F, 1 µM, Paraquat	0.86 (0.0.68–1.07)	
D. melanogaster, F, 10 µM, Paraquat	1.01 (1.0.80–1.26)	
D. melanogaster, F, 100 µM, Paraquat	1.10 (1.0.88–1.38)	
D. melanogaster, F, 1 mM, Paraquat	1.03 (1.0.82–1.30)	
D. melanogaster, F, 10 mM, Paraquat	1.11 (1.0.89–1.39)	
D. melanogaster, F, 100 mM, Paraquat	1.12 (1.0.89–1.40)	
D. melanogaster, M, 10 nM, Starvation	1.15 (1.0.91–1.44)	
D. melanogaster, M, 100 nM, Starvation	1.05 (1.0.84–1.32)	
D. melanogaster, M, 1 µM, Starvation	0.94 (0.0.75–1.18)	
D. melanogaster, M, 10 µM, Starvation	1.01 (1.0.81–1.27)	
D. melanogaster, M, 100 µM, Starvation	0.97 (0.0.77–1.22)	
D. melanogaster, M, 1 mM, Starvation	1.15 (1.0.91–1.45)	
D. melanogaster, M, 10 mM, Starvation	1.01 (1.0.80–1.26)	
D. melanogaster, M, 100 mM, Starvation	1.18 (1.0.94–1.48)	
D. melanogaster, F, 10 nM, Starvation	0.80 (0.0.64–1.01)	
D. melanogaster, F, 100 nM, Starvation	0.81 (0.0.65–1.02)	
D. melanogaster, F, 1 µM, Starvation	0.88 (0.0.70–1.10)	
D. melanogaster, F, 10 µM, Starvation	0.80 (0.0.64–1.01)	
D. melanogaster, F, 100 µM, Starvation	0.88 (0.0.71–1.11)	
D. melanogaster, F, 1 mM, Starvation	0.94 (0.0.75–1.18)	
D. melanogaster, F, 10 mM, Starvation	0.76 (0.0.61–0.95)	*
D. melanogaster, F, 100 mM, Starvation	0.95 (0.0.76–1.19)	
D. melanogaster, M, 10 nM, Hyperoxia	0.90 (0.0.71–1.12)	
D. melanogaster, M, 100 nM, Hyperoxia	0.92 (0.0.73–1.15)	
D. melanogaster, M, 1 µM, Hyperoxia	0.92 (0.0.73–1.16)	
D. melanogaster, M, 10 µM, Hyperoxia	0.89 (0.0.71–1.12)	
D. melanogaster, M, 100 µM, Hyperoxia	0.89 (0.0.71–1.12)	
D. melanogaster, M, 1 mM, Hyperoxia	1.15 (1.0.91–1.45)	
D. melanogaster, M, 10 mM, Hyperoxia	0.98 (0.0.78–1.23)	
D. melanogaster, M, 100 mM, Hyperoxia	1.18 (1.0.94–1.49)	
D. melanogaster, F, 10 nM, Hyperoxia	1.03 (1.0.82–1.30)	
D. melanogaster, F, 100 nM, Hyperoxia	0.92 (0.0.73–1.15)	
D. melanogaster, F, 1 µM, Hyperoxia	1.05 (1.0.83–1.31)	
D. melanogaster, F, 10 µM, Hyperoxia	1.14 (1.0.91–1.44)	
D. melanogaster, F, 100 µM, Hyperoxia	1.14 (1.0.91–1.43)	
D. melanogaster, F, 1 mM, Hyperoxia	1.04 (1.0.83–1.31)	
D. melanogaster, F, 10 mM, Hyperoxia	0.87 (0.0.69–1.09)	
D. melanogaster, F, 100 mM, Hyperoxia	0.59 (0.0.46–0.75)	***
D. virilis, M, 10 nM, Paraquat	0.90 (0.0.71–1.12)	
D. virilis, M, 100 nM, Paraquat	0.95 (0.0.76–1.20)	
D. virilis, M, 1 µM, Paraquat	0.89 (0.0.71–1.12)	
D. virilis, M, 10 µM, Paraquat	0.86 (0.0.69–1.08)	
D. virilis, M, 100 µM, Paraquat	0.84 (0.0.66–1.05)	
D. virilis, M, 1 mM, Paraquat	0.88 (0.0.70–1.10)	
D. virilis, M, 10 mM, Paraquat	1.01 (1.0.80–1.26)	
D. virilis, M, 100 mM, Paraquat	0.93 (0.0.74–1.17)	
D. virilis, F, 10 nM, Paraquat	0.97 (0.0.77–1.22)	
D. virilis, F, 100 nM, Paraquat	1.00 (1.0.79–1.25)	
D. virilis, F, 1 µM, Paraquat	0.97 (0.0.77–1.22)	
D. virilis, F, 10 µM, Paraquat	0.97 (0.0.77–1.21)	
D. virilis, F, 100 µM, Paraquat	0.99 (0.0.79–1.24)	
D. virilis, F, 1 mM, Paraquat	0.90 (0.0.72–1.13)	
D. virilis, F, 10 mM, Paraquat	0.98 (0.0.78–1.23)	
D. virilis, F, 100 mM, Paraquat	1.00 (1.0.80–1.26)	
D. virilis, M, 10 nM, Starvation	1.72 (1.1.35–2.19)	***
D. virilis, M, 100 nM, Starvation	1.44 (1.1.14–1.82)	**
D. virilis, M, 1 µM, Starvation	1.34 (1.1.06–1.70)	*
D. virilis, M, 10 µM, Starvation	1.48 (1.1.17–1.88)	***
D. virilis, M, 100 µM, Starvation	1.05 (1.0.84–1.32)	
D. virilis, M, 1 mM, Starvation	1.25 (1.0.99–1.58)	
D. virilis, M, 10 mM, Starvation	0.89 (0.0.71–1.11)	
D. virilis, M, 100 mM, Starvation	0.82 (0.0.66–1.03)	
D. virilis, F, 10 nM, Starvation	1.05 (1.0.83–1.32)	
D. virilis, F, 100 nM, Starvation	1.13 (1.0.90–1.42)	
D. virilis, F, 1 µM, Starvation	1.15 (1.0.91–1.44)	
D. virilis, F, 10 µM, Starvation	1.04 (1.0.83–1.31)	
D. virilis, F, 100 µM, Starvation	0.90 (0.0.71–1.13)	

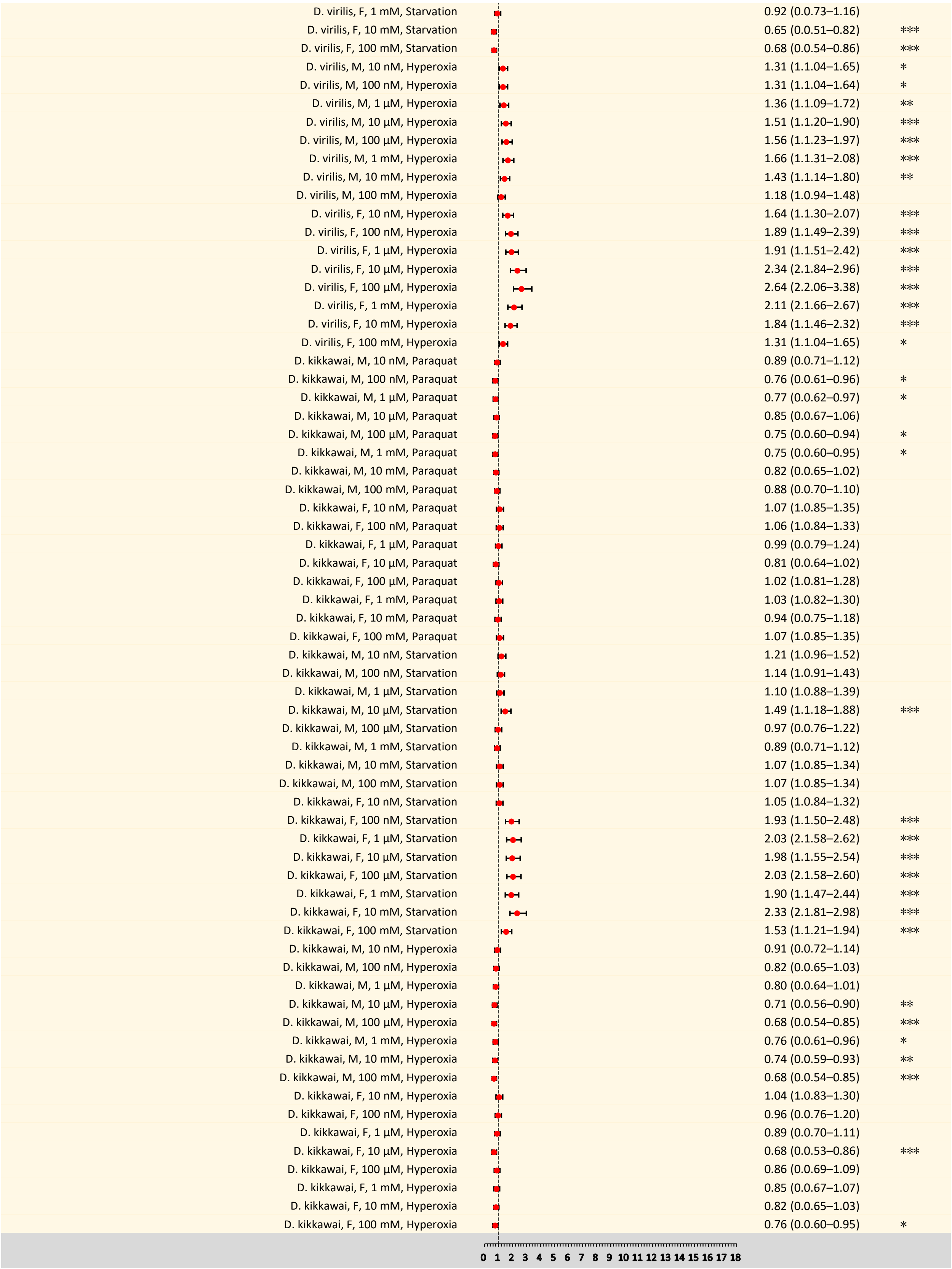

Figure S5. Forest plot of hazard ratios for all *Drosophila* experiments.

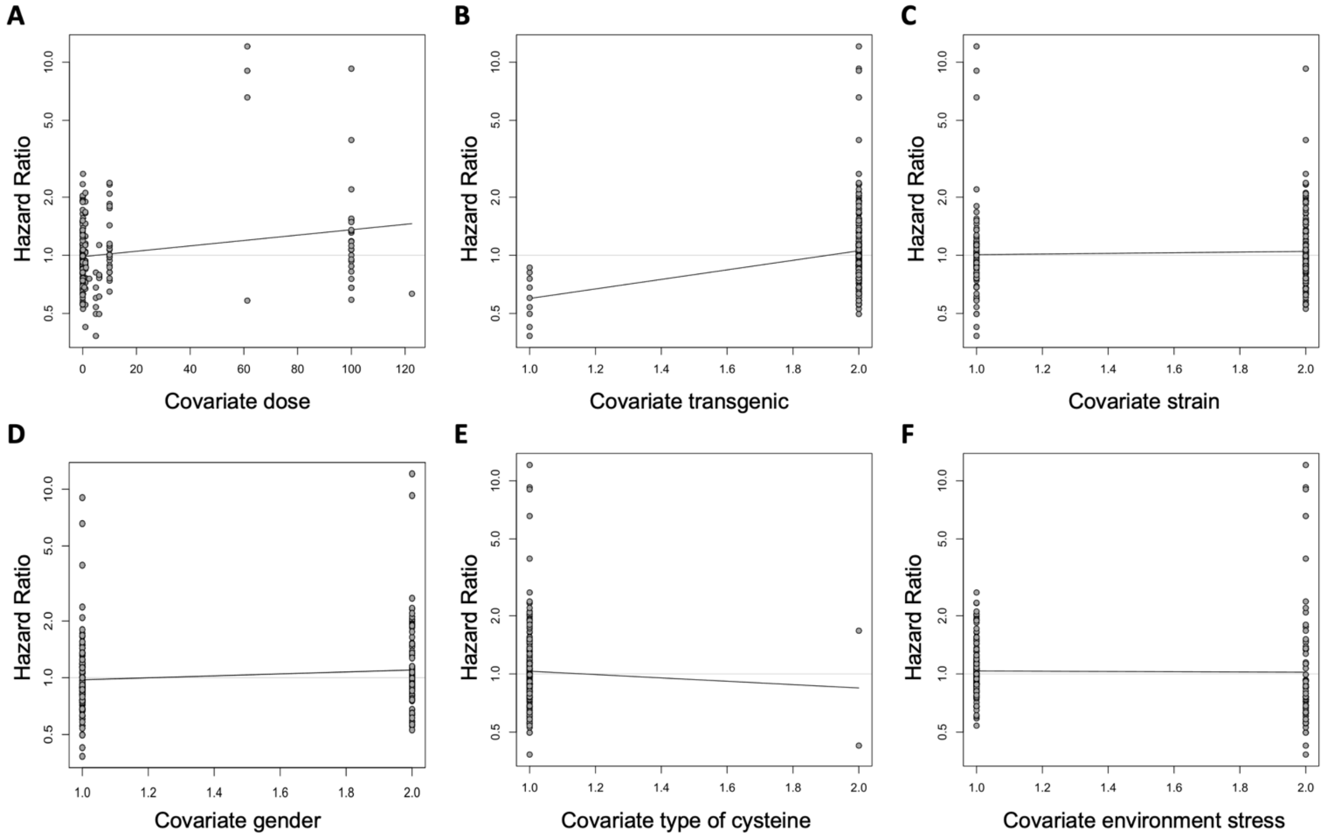

Figure S6. Random-effects meta-regression plots of the association between experimental conditions and HR for all *Drosophila* experiments.

(A) dose; (B) transgenic; (C) strains; (D) gender; (E) type of cysteine; (F) environment stress.

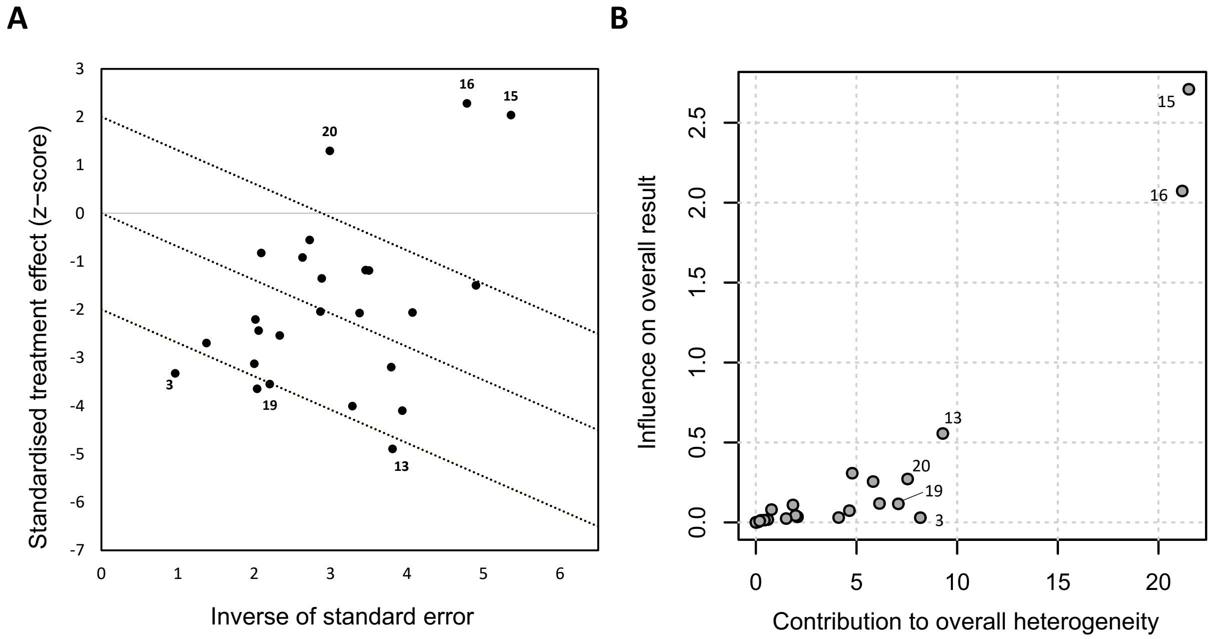

Figure S7. Galbraith plot (A) and Baujat plot (B) for all mice experiments.

(A) In the Galbraith diagram, the x-axis represents the inverse of the standard error (1/*se*), and the y-axis represents the effect size divided by its standard error (log(*HR*)/*se*). A steeper slope of the solid line indicates greater overall heterogeneity. The dotted line represents the 95% confidence interval. Points outside the 95% confidence interval indicate outliers. (B) In the Baujat plot, the x-axis represents the contribution of each study to the overall heterogeneity, while the y-axis represents the standardized squared difference of each study on the overall effect, and the studies closer to the upper right quadrant contribute the most to both factors.

Study	HR	95%-CI	P-value	Tau2	Tau	I2
Omitting Andreassen 2020	0.51	[0.38; 0.67]	< 0.01	0.3406	0.5836	79%
Omitting Chen 2019	0.51	[0.39; 0.67]	< 0.01	0.3364	0.5800	78%
Omitting Chen 2020	0.52	[0.40; 0.68]	< 0.01	0.3001	0.5479	77%
Omitting Flurkey 2010-1	0.49	[0.37; 0.65]	< 0.01	0.3524	0.5937	79%
Omitting Flurkey 2010-2	0.49	[0.37; 0.64]	< 0.01	0.3480	0.5899	79%
Omitting Flurkey 2010-3	0.51	[0.39; 0.67]	< 0.01	0.3225	0.5679	78%
Omitting Flurkey 2010-4	0.51	[0.38; 0.67]	< 0.01	0.3445	0.5869	79%
Omitting Frenke 2019-1	0.49	[0.37; 0.65]	< 0.01	0.3537	0.5948	79%
Omitting Frenke 2019-2	0.49	[0.37; 0.65]	< 0.01	0.3538	0.5948	79%
Omitting Harman 1957-1	0.49	[0.37; 0.65]	< 0.01	0.3589	0.5991	79%
Omitting Harman 1957-2	0.50	[0.38; 0.66]	< 0.01	0.3561	0.5968	79%
Omitting Harman 1957-3	0.51	[0.38; 0.67]	< 0.01	0.3478	0.5897	78%
Omitting Harman 1957-4	0.51	[0.39; 0.68]	< 0.01	0.3297	0.5742	77%
Omitting Harman 1961-1	0.49	[0.37; 0.65]	< 0.01	0.3532	0.5943	79%
Omitting Harman 1961-2	0.48	[0.37; 0.62]	< 0.01	0.2789	0.5281	73%
Omitting Harman 1961-3	0.47	[0.37; 0.61]	< 0.01	0.2670	0.5167	74%
Omitting Jin 2014	0.49	[0.37; 0.65]	< 0.01	0.3511	0.5926	79%
Omitting Kondratov 2009	0.50	[0.38; 0.67]	< 0.01	0.3438	0.5863	79%
Omitting Kumar 2022	0.52	[0.40; 0.68]	< 0.01	0.3068	0.5539	78%
Omitting kurano 2022-1	0.48	[0.37; 0.62]	< 0.01	0.3002	0.5479	78%
Omitting kurano 2022-2	0.50	[0.37; 0.66]	< 0.01	0.3560	0.5967	79%
Omitting kurano 2022-3	0.49	[0.37; 0.65]	< 0.01	0.3556	0.5963	79%
Omitting kurano 2022-4	0.52	[0.40; 0.68]	< 0.01	0.3160	0.5621	78%
Omitting Paul 2014	0.51	[0.39; 0.67]	< 0.01	0.3188	0.5647	78%
Omitting Relien 2006	0.49	[0.37; 0.66]	< 0.01	0.3583	0.5986	79%
**Random effects model**	**0.50**	**[0.38; 0.65]**	**< 0.01**	**0.3338**	**0.5777**	**78%**

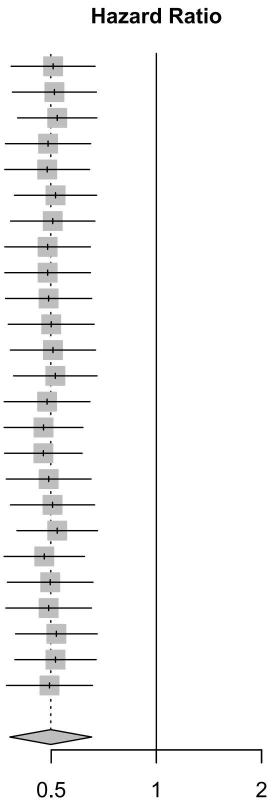

Figure S8. Sensitivity analysis using leave-one-out procedure for all mice experiments.

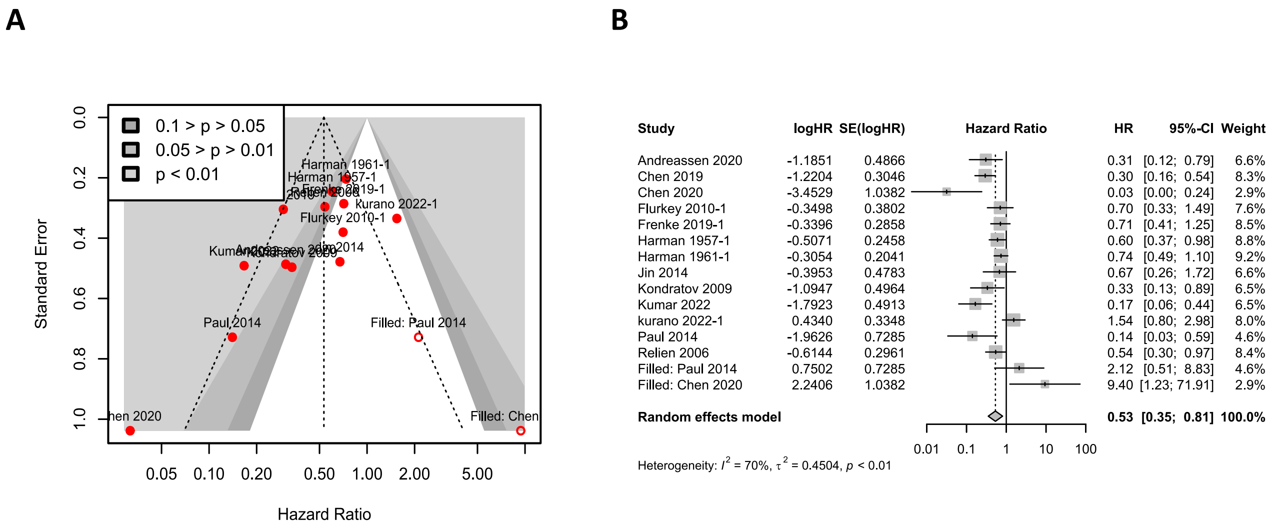

Figure S9. Trim and fill methods to identify and correct asymmetries in funnel plots.

(A) Filled funnel plots with pseudo 95% confidence limits. (B) Forest plot of the random effects model after trim and fill.

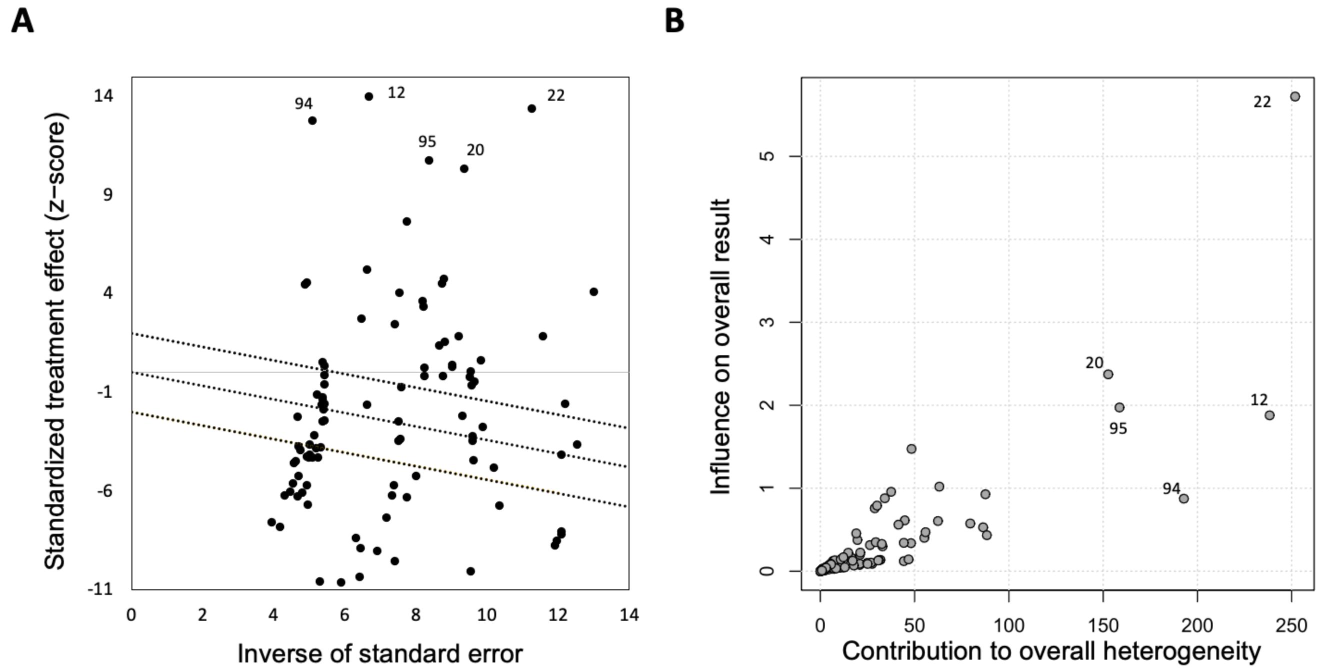

Figure S10. Galbraith plot (A) and Baujat plot (B) for all *C. elegans* experiments.

Hazard Ratio

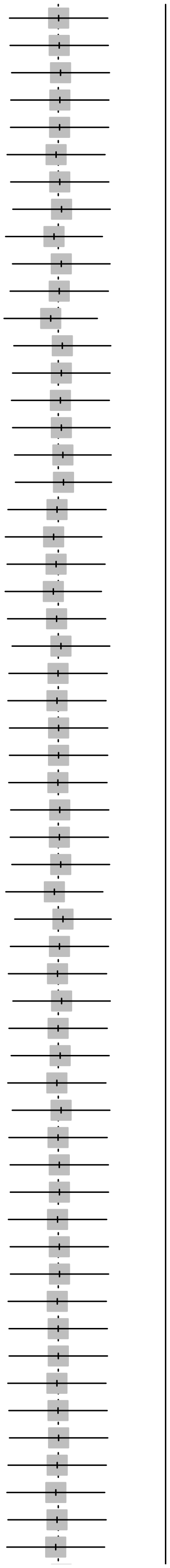

Study	HR	95%-CI	P-value	Tau2	Tau	I2
Omitting Desjardins 2017-1	0.71	[0.60; 0.83]	< 0.01	0.6193	0.7870	97%
Omitting Desjardins 2017-2	0.71	[0.60; 0.83]	< 0.01	0.6184	0.7864	97%
Omitting Desjardins 2017-3	0.71	[0.61; 0.83]	< 0.01	0.6138	0.7835	96%
Omitting Desjardins 2017-4	0.71	[0.60; 0.83]	< 0.01	0.6166	0.7853	97%
Omitting Desjardins 2017-5	0.71	[0.60; 0.83]	< 0.01	0.6175	0.7858	97%
Omitting Desjardins 2017-6	0.70	[0.60; 0.82]	< 0.01	0.6144	0.7839	97%
Omitting Desjardins 2017-7	0.71	[0.60; 0.83]	< 0.01	0.6170	0.7855	97%
Omitting Desjardins 2017-8	0.71	[0.61; 0.84]	< 0.01	0.6080	0.7797	96%
Omitting Desjardins 2017-9	0.70	[0.59; 0.81]	< 0.01	0.5999	0.7745	96%
Omitting Desjardins 2017-10	0.71	[0.61; 0.83]	< 0.01	0.6097	0.7808	96%
Omitting Desjardins 2017-11	0.71	[0.60; 0.83]	< 0.01	0.6183	0.7863	97%
Omitting Desjardins 2017-12	0.69	[0.59; 0.80]	< 0.01	0.5556	0.7454	96%
Omitting Desjardins 2017-13	0.71	[0.61; 0.84]	< 0.01	0.6021	0.7760	96%
Omitting Desjardins 2017-14	0.71	[0.61; 0.83]	< 0.01	0.6095	0.7807	96%
Omitting Desjardins 2017-15	0.71	[0.61; 0.83]	< 0.01	0.6144	0.7838	96%
Omitting Desjardins 2017-16	0.71	[0.61; 0.83]	< 0.01	0.6092	0.7805	96%
Omitting Desjardins 2017-17	0.72	[0.61; 0.84]	< 0.01	0.5971	0.7727	96%
Omitting Desjardins 2017-18	0.72	[0.61; 0.84]	< 0.01	0.5910	0.7688	96%
Omitting Gusarov 2021-1	0.70	[0.60; 0.82]	< 0.01	0.6179	0.7861	97%
Omitting Gusarov 2021-2	0.69	[0.59; 0.81]	< 0.01	0.5961	0.7721	96%
Omitting Gusarov 2021-3	0.70	[0.60; 0.82]	< 0.01	0.6146	0.7840	96%
Omitting Gusarov 2021-4	0.69	[0.59; 0.81]	< 0.01	0.5930	0.7700	96%
Omitting Gusarov 2021-5	0.70	[0.60; 0.82]	< 0.01	0.6167	0.7853	97%
Omitting Kim 2016-1	0.71	[0.61; 0.83]	< 0.01	0.6114	0.7819	97%
Omitting Kim 2016-2	0.70	[0.60; 0.83]	< 0.01	0.6192	0.7869	97%
Omitting Kim 2016-3	0.70	[0.60; 0.82]	< 0.01	0.6175	0.7858	97%
Omitting Kim 2017-1	0.71	[0.60; 0.83]	< 0.01	0.6191	0.7868	97%
Omitting Kim 2017-2	0.71	[0.60; 0.83]	< 0.01	0.6191	0.7868	97%
Omitting Kim 2017-3	0.70	[0.60; 0.83]	< 0.01	0.6191	0.7868	97%
Omitting Kim 2017-4	0.71	[0.60; 0.83]	< 0.01	0.6168	0.7854	97%
Omitting Kim 2017-5	0.71	[0.60; 0.83]	< 0.01	0.6178	0.7860	97%
Omitting Kim 2017-6	0.71	[0.61; 0.83]	< 0.01	0.6126	0.7827	97%
Omitting Kim 2018-1	0.70	[0.59; 0.82]	< 0.01	0.6029	0.7765	96%
Omitting Kim 2018-2	0.72	[0.61; 0.84]	< 0.01	0.5959	0.7719	96%
Omitting Kim 2018-3	0.71	[0.60; 0.83]	< 0.01	0.6176	0.7859	97%
Omitting Kim 2018-4	0.70	[0.60; 0.83]	< 0.01	0.6187	0.7866	97%
Omitting Kim 2018-5	0.71	[0.61; 0.84]	< 0.01	0.6073	0.7793	97%
Omitting Kim 2018-6	0.71	[0.60; 0.83]	< 0.01	0.6192	0.7869	97%
Omitting Kim 2018-7	0.71	[0.61; 0.83]	< 0.01	0.6152	0.7844	97%
Omitting Kim 2018-8	0.70	[0.60; 0.82]	< 0.01	0.6172	0.7856	97%
Omitting Kim 2018-9	0.71	[0.61; 0.83]	< 0.01	0.6107	0.7815	97%
Omitting Ogawa 2016-1	0.70	[0.60; 0.83]	< 0.01	0.6195	0.7871	97%
Omitting Ogawa 2016-2	0.71	[0.60; 0.83]	< 0.01	0.6184	0.7864	97%
Omitting Ogawa 2016-3	0.71	[0.60; 0.83]	< 0.01	0.6180	0.7861	96%
Omitting Ogawa 2016-4	0.70	[0.60; 0.83]	< 0.01	0.6190	0.7868	97%
Omitting Ogawa 2016-5	0.71	[0.60; 0.83]	< 0.01	0.6183	0.7863	97%
Omitting Ogawa 2016-6	0.71	[0.60; 0.83]	< 0.01	0.6178	0.7860	96%
Omitting Ogawa 2016-7	0.70	[0.60; 0.82]	< 0.01	0.6185	0.7864	97%
Omitting Ogawa 2016-8	0.71	[0.60; 0.83]	< 0.01	0.6195	0.7871	97%
Omitting Ogawa 2016-9	0.71	[0.60; 0.83]	< 0.01	0.6195	0.7871	97%
Omitting Ogawa 2016-10	0.70	[0.60; 0.82]	< 0.01	0.6176	0.7859	97%
Omitting Ogawa 2016-11	0.70	[0.60; 0.83]	< 0.01	0.6194	0.7870	97%
Omitting Ogawa 2016-12	0.71	[0.60; 0.83]	< 0.01	0.6193	0.7870	97%
Omitting Ogawa 2016-13	0.70	[0.60; 0.82]	< 0.01	0.6183	0.7863	97%
Omitting Ogawa 2016-14	0.70	[0.60; 0.82]	< 0.01	0.6133	0.7831	97%
Omitting Ogawa 2016-15	0.70	[0.60; 0.82]	< 0.01	0.6179	0.7861	97%
Omitting Ogawa 2016-16	0.70	[0.60; 0.82]	< 0.01	0.6126	0.7827	97%

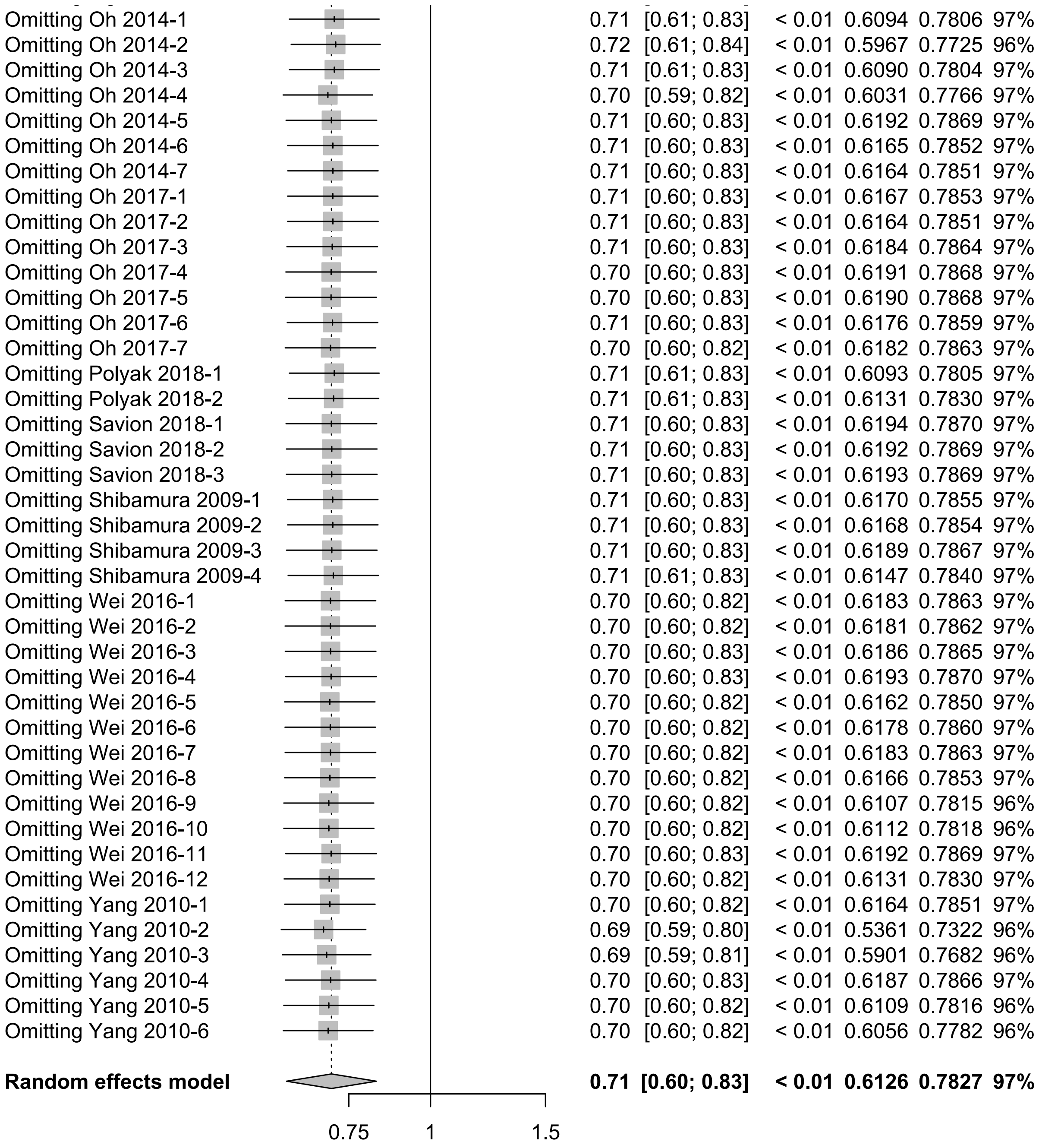

Figure S11. Sensitivity analysis using leave-one-out procedure for all *C. elegans* experiments.

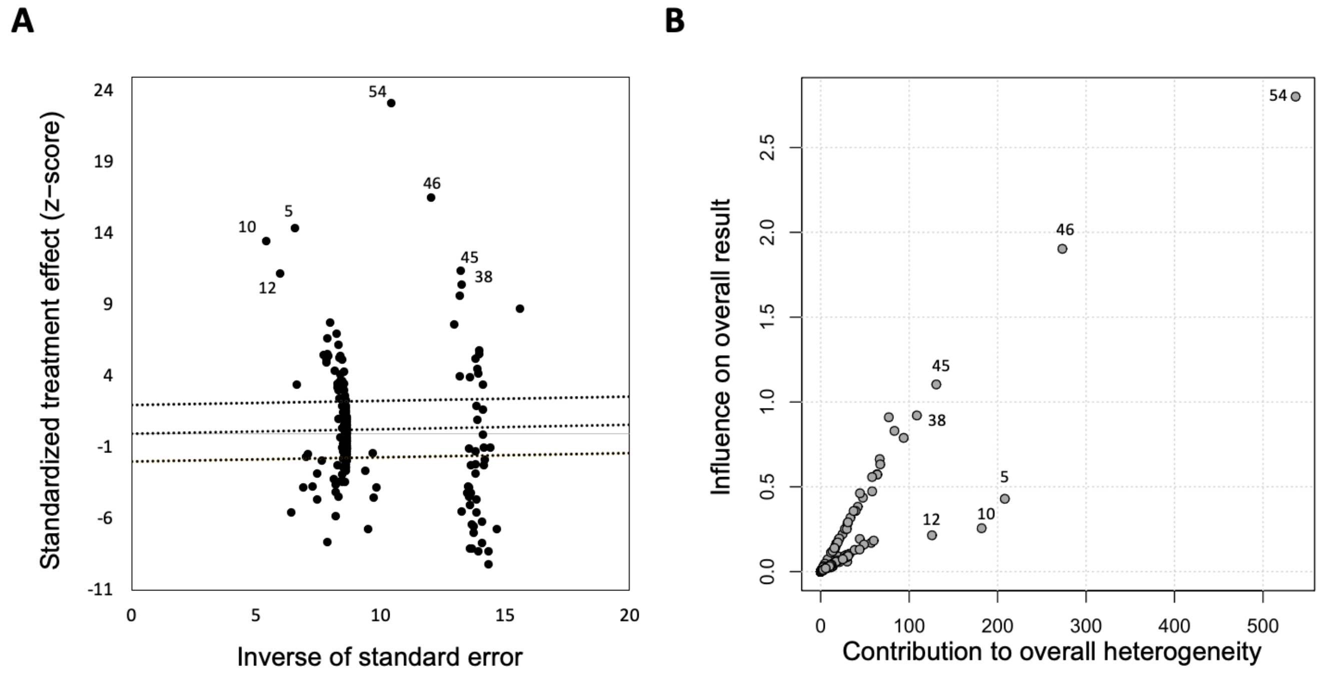

Figure S12. Galbraith plot (A) and Baujat plot (B) for all *Drosophila* experiments.

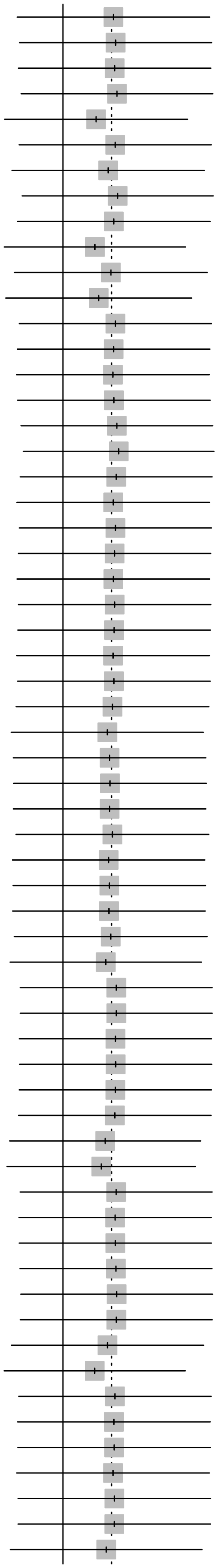

Study	Hazard Ratio	HR	95%-CI	P-value	Tau2	Tau	I2
Omitting Brack 1997-1		1.03	[0.97; 1.10]	0.30	0.1967	0.4435	95%
Omitting Brack 1997-2		1.03	[0.97; 1.10]	0.28	0.1955	0.4422	95%
Omitting Brack 1997-3		1.03	[0.97; 1.10]	0.29	0.1962	0.4430	95%
Omitting Brack 1997-4		1.04	[0.97; 1.10]	0.27	0.1944	0.4409	95%
Omitting Brack 1997-5		1.02	[0.96; 1.08]	0.48	0.1763	0.4199	94%
Omitting Brack 1997-6		1.03	[0.97; 1.10]	0.29	0.1959	0.4426	95%
Omitting Jouandin 2022-1		1.03	[0.97; 1.09]	0.36	0.1959	0.4427	95%
Omitting Jouandin 2022-2		1.04	[0.97; 1.10]	0.26	0.1936	0.4400	95%
Omitting Niraula 2019-1		1.03	[0.97; 1.10]	0.30	0.1966	0.4434	95%
Omitting Niraula 2019-2		1.02	[0.96; 1.08]	0.49	0.1731	0.4161	94%
Omitting Niraula 2019-3		1.03	[0.97; 1.10]	0.33	0.1970	0.4438	95%
Omitting Niraula 2019-4		1.02	[0.96; 1.09]	0.45	0.1826	0.4273	94%
Omitting Niraula 2019-5		1.03	[0.97; 1.10]	0.28	0.1957	0.4424	95%
Omitting Niraula 2019-6		1.03	[0.97; 1.10]	0.30	0.1967	0.4435	95%
Omitting Russi 2020-1		1.03	[0.97; 1.10]	0.31	0.1969	0.4437	95%
Omitting Russi 2020-2		1.03	[0.97; 1.10]	0.30	0.1966	0.4434	95%
Omitting Russi 2020-3		1.03	[0.97; 1.10]	0.27	0.1945	0.4410	95%
Omitting Russi 2020-4		1.04	[0.98; 1.10]	0.25	0.1924	0.4386	95%
Omitting Russi 2020-5		1.03	[0.97; 1.10]	0.28	0.1951	0.4417	95%
Omitting Russi 2020-6		1.03	[0.97; 1.10]	0.31	0.1968	0.4436	95%
Omitting Russi 2020-7		1.03	[0.97; 1.10]	0.28	0.1957	0.4424	95%
Omitting Russi 2020-8		1.03	[0.97; 1.10]	0.29	0.1962	0.4430	95%
Omitting Shaposhnikov 2018-1		1.03	[0.97; 1.10]	0.30	0.1968	0.4436	95%
Omitting Shaposhnikov 2018-2		1.03	[0.97; 1.10]	0.29	0.1963	0.4430	95%
Omitting Shaposhnikov 2018-3		1.03	[0.97; 1.10]	0.30	0.1965	0.4433	95%
Omitting Shaposhnikov 2018-4		1.03	[0.97; 1.10]	0.31	0.1969	0.4437	95%
Omitting Shaposhnikov 2018-5		1.03	[0.97; 1.10]	0.30	0.1966	0.4434	95%
Omitting Shaposhnikov 2018-6		1.03	[0.97; 1.10]	0.31	0.1970	0.4439	95%
Omitting Shaposhnikov 2018-7		1.03	[0.97; 1.09]	0.36	0.1955	0.4421	95%
Omitting Shaposhnikov 2018-8		1.03	[0.97; 1.10]	0.34	0.1967	0.4435	95%
Omitting Shaposhnikov 2018-9		1.03	[0.97; 1.10]	0.34	0.1968	0.4437	95%
Omitting Shaposhnikov 2018-10		1.03	[0.97; 1.10]	0.34	0.1967	0.4435	95%
Omitting Shaposhnikov 2018-11		1.03	[0.97; 1.10]	0.31	0.1970	0.4439	95%
Omitting Shaposhnikov 2018-12		1.03	[0.97; 1.09]	0.35	0.1963	0.4431	95%
Omitting Shaposhnikov 2018-13		1.03	[0.97; 1.10]	0.35	0.1966	0.4434	95%
Omitting Shaposhnikov 2018-14		1.03	[0.97; 1.09]	0.35	0.1964	0.4432	95%
Omitting Shaposhnikov 2018-15		1.03	[0.97; 1.10]	0.33	0.1970	0.4439	95%
Omitting Shaposhnikov 2018-16		1.03	[0.97; 1.09]	0.38	0.1941	0.4405	94%
Omitting Shaposhnikov 2018-17		1.03	[0.97; 1.10]	0.28	0.1951	0.4417	95%
Omitting Shaposhnikov 2018-18		1.03	[0.97; 1.10]	0.28	0.1951	0.4417	95%
Omitting Shaposhnikov 2018-19		1.03	[0.97; 1.10]	0.28	0.1958	0.4424	95%
Omitting Shaposhnikov 2018-20		1.03	[0.97; 1.10]	0.28	0.1956	0.4423	95%
Omitting Shaposhnikov 2018-21		1.03	[0.97; 1.10]	0.29	0.1958	0.4425	95%
Omitting Shaposhnikov 2018-22		1.03	[0.97; 1.10]	0.29	0.1961	0.4429	95%
Omitting Shaposhnikov 2018-23		1.03	[0.97; 1.09]	0.39	0.1934	0.4398	94%
Omitting Shaposhnikov 2018-24		1.02	[0.96; 1.09]	0.43	0.1877	0.4332	94%
Omitting Shaposhnikov 2018-25		1.03	[0.97; 1.10]	0.28	0.1952	0.4418	95%
Omitting Shaposhnikov 2018-26		1.03	[0.97; 1.10]	0.29	0.1960	0.4427	95%
Omitting Shaposhnikov 2018-27		1.03	[0.97; 1.10]	0.29	0.1958	0.4425	95%
Omitting Shaposhnikov 2018-28		1.03	[0.97; 1.10]	0.28	0.1954	0.4420	95%
Omitting Shaposhnikov 2018-29		1.03	[0.97; 1.10]	0.27	0.1947	0.4413	94%
Omitting Shaposhnikov 2018-30		1.03	[0.97; 1.10]	0.28	0.1951	0.4417	95%
Omitting Shaposhnikov 2018-31		1.03	[0.97; 1.09]	0.36	0.1956	0.4422	95%
Omitting Shaposhnikov 2018-32		1.02	[0.96; 1.08]	0.49	0.1725	0.4154	94%
Omitting Shaposhnikov 2018-33		1.03	[0.97; 1.10]	0.29	0.1961	0.4428	95%
Omitting Shaposhnikov 2018-34		1.03	[0.97; 1.10]	0.30	0.1966	0.4434	95%
Omitting Shaposhnikov 2018-35		1.03	[0.97; 1.10]	0.30	0.1965	0.4433	95%
Omitting Shaposhnikov 2018-36		1.03	[0.97; 1.10]	0.31	0.1969	0.4437	95%
Omitting Shaposhnikov 2018-37		1.03	[0.97; 1.10]	0.30	0.1964	0.4432	95%
Omitting Shaposhnikov 2018-38		1.03	[0.97; 1.10]	0.30	0.1964	0.4432	95%
Omitting Shaposhnikov 2018-39		1.03	[0.97; 1.09]	0.38	0.1945	0.4410	94%

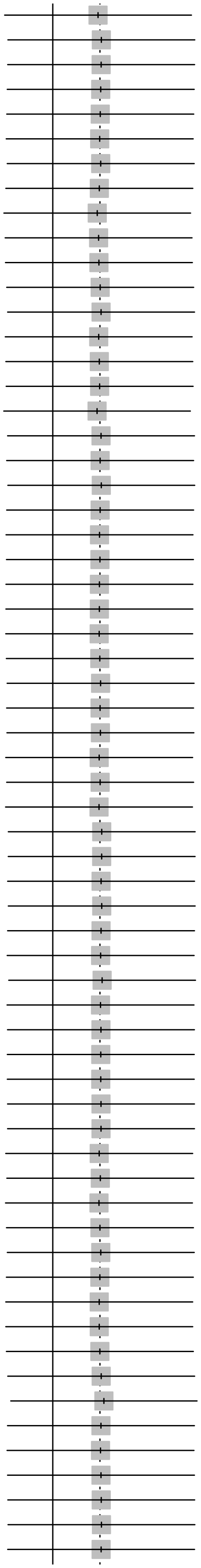

Omitting Shaposhnikov 2018-40	1.03	[0.97; 1.10]	0.34	0.1967	0.4435	95%
Omitting Shaposhnikov 2018-41	1.03	[0.97; 1.10]	0.31	0.1969	0.4437	95%
Omitting Shaposhnikov 2018-42	1.03	[0.97; 1.10]	0.31	0.1970	0.4438	95%
Omitting Shaposhnikov 2018-43	1.03	[0.97; 1.10]	0.31	0.1970	0.4439	95%
Omitting Shaposhnikov 2018-44	1.03	[0.97; 1.10]	0.32	0.1971	0.4439	95%
Omitting Shaposhnikov 2018-45	1.03	[0.97; 1.10]	0.33	0.1971	0.4439	95%
Omitting Shaposhnikov 2018-46	1.03	[0.97; 1.10]	0.31	0.1970	0.4439	95%
Omitting Shaposhnikov 2018-47	1.03	[0.97; 1.10]	0.33	0.1970	0.4439	95%
Omitting Shaposhnikov 2018-48	1.03	[0.97; 1.09]	0.35	0.1964	0.4431	95%
Omitting Shaposhnikov 2018-49	1.03	[0.97; 1.10]	0.34	0.1969	0.4437	95%
Omitting Shaposhnikov 2018-50	1.03	[0.97; 1.10]	0.33	0.1969	0.4438	95%
Omitting Shaposhnikov 2018-51	1.03	[0.97; 1.10]	0.32	0.1970	0.4439	95%
Omitting Shaposhnikov 2018-52	1.03	[0.97; 1.10]	0.31	0.1969	0.4438	95%
Omitting Shaposhnikov 2018-53	1.03	[0.97; 1.10]	0.34	0.1969	0.4437	95%
Omitting Shaposhnikov 2018-54	1.03	[0.97; 1.10]	0.33	0.1970	0.4438	95%
Omitting Shaposhnikov 2018-55	1.03	[0.97; 1.10]	0.33	0.1970	0.4439	95%
Omitting Shaposhnikov 2018-56	1.03	[0.97; 1.09]	0.35	0.1962	0.4430	95%
Omitting Shaposhnikov 2018-57	1.03	[0.97; 1.10]	0.31	0.1969	0.4438	95%
Omitting Shaposhnikov 2018-58	1.03	[0.97; 1.10]	0.32	0.1970	0.4439	95%
Omitting Shaposhnikov 2018-59	1.03	[0.97; 1.10]	0.31	0.1969	0.4437	95%
Omitting Shaposhnikov 2018-60	1.03	[0.97; 1.10]	0.32	0.1970	0.4439	95%
Omitting Shaposhnikov 2018-61	1.03	[0.97; 1.10]	0.33	0.1970	0.4439	95%
Omitting Shaposhnikov 2018-62	1.03	[0.97; 1.10]	0.32	0.1970	0.4439	95%
Omitting Shaposhnikov 2018-63	1.03	[0.97; 1.10]	0.33	0.1970	0.4439	95%
Omitting Shaposhnikov 2018-64	1.03	[0.97; 1.10]	0.33	0.1970	0.4439	95%
Omitting Shaposhnikov 2018-65	1.03	[0.97; 1.10]	0.33	0.1970	0.4438	95%
Omitting Shaposhnikov 2018-66	1.03	[0.97; 1.10]	0.32	0.1970	0.4439	95%
Omitting Shaposhnikov 2018-67	1.03	[0.97; 1.10]	0.32	0.1970	0.4439	95%
Omitting Shaposhnikov 2018-68	1.03	[0.97; 1.10]	0.32	0.1970	0.4439	95%
Omitting Shaposhnikov 2018-69	1.03	[0.97; 1.10]	0.32	0.1970	0.4439	95%
Omitting Shaposhnikov 2018-70	1.03	[0.97; 1.10]	0.33	0.1970	0.4438	95%
Omitting Shaposhnikov 2018-71	1.03	[0.97; 1.10]	0.32	0.1970	0.4439	95%
Omitting Shaposhnikov 2018-72	1.03	[0.97; 1.10]	0.33	0.1970	0.4438	95%
Omitting Shaposhnikov 2018-73	1.03	[0.97; 1.10]	0.30	0.1967	0.4436	95%
Omitting Shaposhnikov 2018-74	1.03	[0.97; 1.10]	0.31	0.1968	0.4436	95%
Omitting Shaposhnikov 2018-75	1.03	[0.97; 1.10]	0.31	0.1969	0.4438	95%
Omitting Shaposhnikov 2018-76	1.03	[0.97; 1.10]	0.30	0.1967	0.4436	95%
Omitting Shaposhnikov 2018-77	1.03	[0.97; 1.10]	0.31	0.1969	0.4438	95%
Omitting Shaposhnikov 2018-78	1.03	[0.97; 1.10]	0.32	0.1970	0.4438	95%
Omitting Shaposhnikov 2018-79	1.03	[0.97; 1.10]	0.30	0.1966	0.4434	95%
Omitting Shaposhnikov 2018-80	1.03	[0.97; 1.10]	0.32	0.1970	0.4439	95%
Omitting Shaposhnikov 2018-81	1.03	[0.97; 1.10]	0.31	0.1969	0.4438	95%
Omitting Shaposhnikov 2018-82	1.03	[0.97; 1.10]	0.31	0.1970	0.4438	95%
Omitting Shaposhnikov 2018-83	1.03	[0.97; 1.10]	0.31	0.1970	0.4438	95%
Omitting Shaposhnikov 2018-84	1.03	[0.97; 1.10]	0.31	0.1969	0.4438	95%
Omitting Shaposhnikov 2018-85	1.03	[0.97; 1.10]	0.31	0.1969	0.4438	95%
Omitting Shaposhnikov 2018-86	1.03	[0.97; 1.10]	0.33	0.1970	0.4438	95%
Omitting Shaposhnikov 2018-87	1.03	[0.97; 1.10]	0.32	0.1970	0.4439	95%
Omitting Shaposhnikov 2018-88	1.03	[0.97; 1.10]	0.33	0.1969	0.4438	95%
Omitting Shaposhnikov 2018-89	1.03	[0.97; 1.10]	0.32	0.1970	0.4439	95%
Omitting Shaposhnikov 2018-90	1.03	[0.97; 1.10]	0.31	0.1970	0.4438	95%
Omitting Shaposhnikov 2018-91	1.03	[0.97; 1.10]	0.32	0.1970	0.4439	95%
Omitting Shaposhnikov 2018-92	1.03	[0.97; 1.10]	0.33	0.1970	0.4438	95%
Omitting Shaposhnikov 2018-93	1.03	[0.97; 1.10]	0.33	0.1970	0.4438	95%
Omitting Shaposhnikov 2018-94	1.03	[0.97; 1.10]	0.32	0.1970	0.4439	95%
Omitting Shaposhnikov 2018-95	1.03	[0.97; 1.10]	0.31	0.1969	0.4437	95%
Omitting Shaposhnikov 2018-96	1.03	[0.97; 1.10]	0.28	0.1955	0.4422	95%
Omitting Shaposhnikov 2018-97	1.03	[0.97; 1.10]	0.31	0.1969	0.4438	95%
Omitting Shaposhnikov 2018-98	1.03	[0.97; 1.10]	0.32	0.1970	0.4439	95%
Omitting Shaposhnikov 2018-99	1.03	[0.97; 1.10]	0.31	0.1969	0.4438	95%
Omitting Shaposhnikov 2018-100	1.03	[0.97; 1.10]	0.31	0.1969	0.4437	95%
Omitting Shaposhnikov 2018-101	1.03	[0.97; 1.10]	0.31	0.1968	0.4437	95%
Omitting Shaposhnikov 2018-102	1.03	[0.97; 1.10]	0.31	0.1969	0.4437	95%

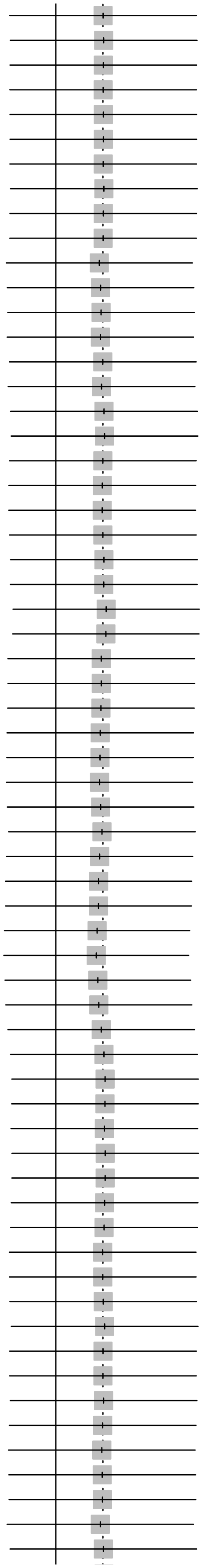

Omitting Shaposhnikov 2018-103	1.03	[0.97; 1.10]	0.32	0.1970	0.4439	95%
Omitting Shaposhnikov 2018-104	1.03	[0.97; 1.10]	0.32	0.1970	0.4438	95%
Omitting Shaposhnikov 2018-105	1.03	[0.97; 1.10]	0.32	0.1970	0.4439	95%
Omitting Shaposhnikov 2018-106	1.03	[0.97; 1.10]	0.32	0.1970	0.4439	95%
Omitting Shaposhnikov 2018-107	1.03	[0.97; 1.10]	0.32	0.1970	0.4439	95%
Omitting Shaposhnikov 2018-108	1.03	[0.97; 1.10]	0.32	0.1970	0.4439	95%
Omitting Shaposhnikov 2018-109	1.03	[0.97; 1.10]	0.32	0.1970	0.4439	95%
Omitting Shaposhnikov 2018-110	1.03	[0.97; 1.10]	0.31	0.1970	0.4438	95%
Omitting Shaposhnikov 2018-111	1.03	[0.97; 1.10]	0.32	0.1970	0.4439	95%
Omitting Shaposhnikov 2018-112	1.03	[0.97; 1.10]	0.32	0.1970	0.4439	95%
Omitting Shaposhnikov 2018-113	1.03	[0.97; 1.09]	0.36	0.1958	0.4425	95%
Omitting Shaposhnikov 2018-114	1.03	[0.97; 1.10]	0.35	0.1965	0.4433	95%
Omitting Shaposhnikov 2018-115	1.03	[0.97; 1.10]	0.34	0.1967	0.4435	95%
Omitting Shaposhnikov 2018-116	1.03	[0.97; 1.09]	0.35	0.1964	0.4432	95%
Omitting Shaposhnikov 2018-117	1.03	[0.97; 1.10]	0.32	0.1970	0.4439	95%
Omitting Shaposhnikov 2018-118	1.03	[0.97; 1.10]	0.34	0.1968	0.4437	95%
Omitting Shaposhnikov 2018-119	1.03	[0.97; 1.10]	0.31	0.1969	0.4438	95%
Omitting Shaposhnikov 2018-120	1.03	[0.97; 1.10]	0.31	0.1968	0.4436	95%
Omitting Shaposhnikov 2018-121	1.03	[0.97; 1.10]	0.32	0.1970	0.4439	95%
Omitting Shaposhnikov 2018-122	1.03	[0.97; 1.10]	0.33	0.1970	0.4438	95%
Omitting Shaposhnikov 2018-123	1.03	[0.97; 1.10]	0.33	0.1970	0.4438	95%
Omitting Shaposhnikov 2018-124	1.03	[0.97; 1.10]	0.32	0.1970	0.4439	95%
Omitting Shaposhnikov 2018-125	1.03	[0.97; 1.10]	0.31	0.1969	0.4438	95%
Omitting Shaposhnikov 2018-126	1.03	[0.97; 1.10]	0.31	0.1970	0.4438	95%
Omitting Shaposhnikov 2018-127	1.03	[0.97; 1.10]	0.29	0.1960	0.4427	95%
Omitting Shaposhnikov 2018-128	1.03	[0.97; 1.10]	0.29	0.1962	0.4430	95%
Omitting Shaposhnikov 2018-129	1.03	[0.97; 1.10]	0.34	0.1968	0.4436	95%
Omitting Shaposhnikov 2018-130	1.03	[0.97; 1.10]	0.34	0.1968	0.4436	95%
Omitting Shaposhnikov 2018-131	1.03	[0.97; 1.10]	0.34	0.1967	0.4435	95%
Omitting Shaposhnikov 2018-132	1.03	[0.97; 1.09]	0.35	0.1963	0.4431	95%
Omitting Shaposhnikov 2018-133	1.03	[0.97; 1.09]	0.35	0.1962	0.4430	95%
Omitting Shaposhnikov 2018-134	1.03	[0.97; 1.09]	0.36	0.1959	0.4427	95%
Omitting Shaposhnikov 2018-135	1.03	[0.97; 1.10]	0.35	0.1965	0.4433	95%
Omitting Shaposhnikov 2018-136	1.03	[0.97; 1.10]	0.33	0.1969	0.4438	95%
Omitting Shaposhnikov 2018-137	1.03	[0.97; 1.09]	0.36	0.1960	0.4427	95%
Omitting Shaposhnikov 2018-138	1.03	[0.97; 1.09]	0.37	0.1953	0.4419	95%
Omitting Shaposhnikov 2018-139	1.03	[0.97; 1.09]	0.37	0.1952	0.4418	95%
Omitting Shaposhnikov 2018-140	1.03	[0.97; 1.09]	0.38	0.1938	0.4402	95%
Omitting Shaposhnikov 2018-141	1.03	[0.97; 1.09]	0.39	0.1928	0.4391	95%
Omitting Shaposhnikov 2018-142	1.03	[0.97; 1.09]	0.37	0.1946	0.4411	95%
Omitting Shaposhnikov 2018-143	1.03	[0.97; 1.09]	0.37	0.1954	0.4420	95%
Omitting Shaposhnikov 2018-144	1.03	[0.97; 1.10]	0.34	0.1968	0.4436	95%
Omitting Shaposhnikov 2018-145	1.03	[0.97; 1.10]	0.31	0.1969	0.4438	95%
Omitting Shaposhnikov 2018-146	1.03	[0.97; 1.10]	0.30	0.1966	0.4434	95%
Omitting Shaposhnikov 2018-147	1.03	[0.97; 1.10]	0.30	0.1966	0.4434	95%
Omitting Shaposhnikov 2018-148	1.03	[0.97; 1.10]	0.31	0.1969	0.4437	95%
Omitting Shaposhnikov 2018-149	1.03	[0.97; 1.10]	0.30	0.1966	0.4433	95%
Omitting Shaposhnikov 2018-150	1.03	[0.97; 1.10]	0.30	0.1966	0.4434	95%
Omitting Shaposhnikov 2018-151	1.03	[0.97; 1.10]	0.31	0.1968	0.4436	95%
Omitting Shaposhnikov 2018-152	1.03	[0.97; 1.10]	0.31	0.1969	0.4438	95%
Omitting Shaposhnikov 2018-153	1.03	[0.97; 1.10]	0.33	0.1970	0.4439	95%
Omitting Shaposhnikov 2018-154	1.03	[0.97; 1.10]	0.32	0.1970	0.4439	95%
Omitting Shaposhnikov 2018-155	1.03	[0.97; 1.10]	0.32	0.1970	0.4439	95%
Omitting Shaposhnikov 2018-156	1.03	[0.97; 1.10]	0.30	0.1968	0.4436	95%
Omitting Shaposhnikov 2018-157	1.03	[0.97; 1.10]	0.32	0.1970	0.4439	95%
Omitting Shaposhnikov 2018-158	1.03	[0.97; 1.10]	0.32	0.1970	0.4439	95%
Omitting Shaposhnikov 2018-159	1.03	[0.97; 1.10]	0.32	0.1970	0.4438	95%
Omitting Shaposhnikov 2018-160	1.03	[0.97; 1.10]	0.33	0.1970	0.4439	95%
Omitting Shaposhnikov 2018-161	1.03	[0.97; 1.10]	0.33	0.1969	0.4438	95%
Omitting Shaposhnikov 2018-162	1.03	[0.97; 1.10]	0.33	0.1970	0.4438	95%
Omitting Shaposhnikov 2018-163	1.03	[0.97; 1.10]	0.33	0.1970	0.4439	95%
Omitting Shaposhnikov 2018-164	1.03	[0.97; 1.09]	0.35	0.1964	0.4432	95%
Omitting Shaposhnikov 2018-165	1.03	[0.97; 1.10]	0.32	0.1970	0.4439	95%

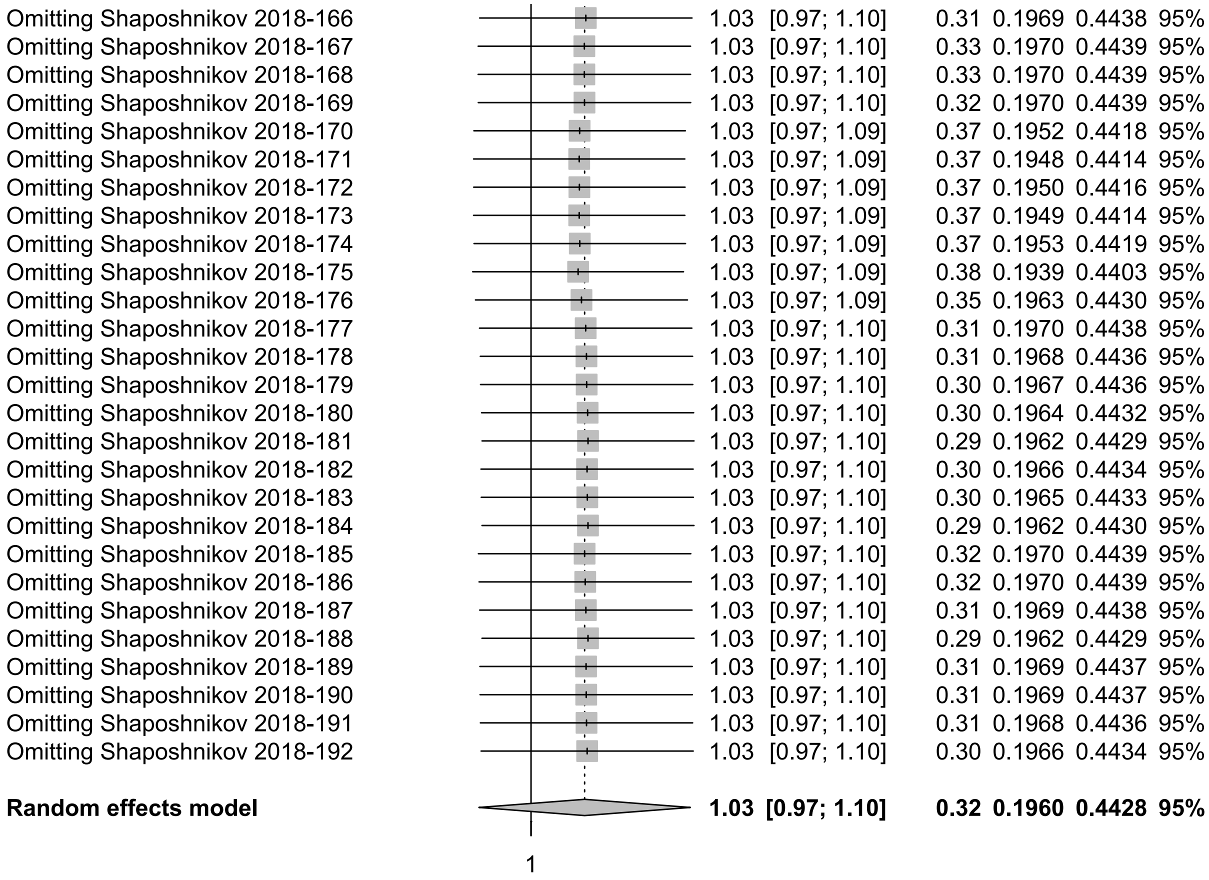

Figure S13. Sensitivity analysis using leave-one-out procedure for all *Drosophila* experiments.

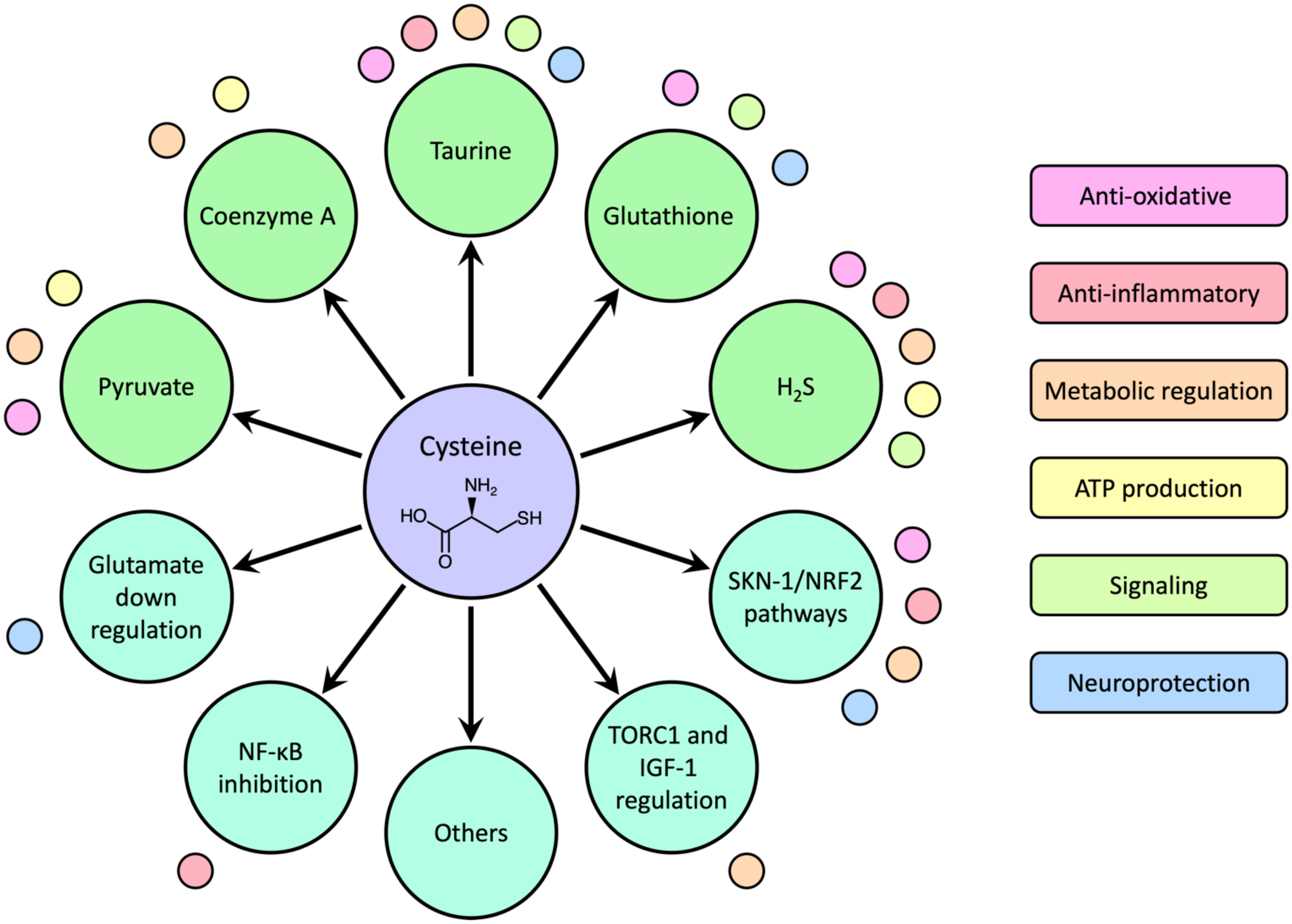

Figure S14. Mechanisms of action of cysteine.

Cysteine is the metabolic precursor of H_2S, glutathione, taurine, coenzyme A, and pyruvate. These compounds participate in nutrient sensing and metabolic regulation, regulate cell signaling pathways, and exhibit anti-oxidative and/or anti-inflammatory activities. Cysteine and its derivatives modulate various aging-related cellular pathways, including the detoxifying and antioxidant SKN-1/NRF2 pathways, the metabolism-regulating TORC1 and IGF-1 pathways, the pro-inflammatory NF-κB pathway, and neurotransmitter systems. Additionally, as a proteinogenic amino acid, cysteine can form disulfide bonds in proteins. Its derivatives have mucolytic activity because they can disrupt disulfide bonds in mucus glycoproteins. Furthermore, dietary cysteine reduces food intake, potentially prolonging lifespan through dietary restriction.
